# Supplementary material for: Recombinant production platform for Group A Streptococcus glycoconjugate vaccines
Source: NPJ Vaccines. 2025 Jan 22;10:16. doi: 10.1038/s41541-025-01068-2 (PMC11754613; doi:10.1038/s41541-025-01068-2)

Supplementary PDF File with Figures and Data

Supplementary Figure 1
A) *E. coli* cells W3110 containing the *waaL* gene transformed with either Shi1 or Shi2a gene clusters and the RhaPS cluster (ΔB) were grown in different media and analysed for GAC-antibody detection in dot blots. Technical duplicates are shown for the two construct combinations. B) *E. coli* cells transformed with the Shi2a, the RhaPS cluster (ΔB) and IdeS plasmids were tested for glycoconjugate production in different growth conditions. Total cell lysates were run over SDS-PAGE and analysed via His and GAC-antibodies (red and green, respectively). Un = uninduced, -*pglB* = cells lack the chromosomal gene *pglB*. Arrows indicate glycosylated protein bands.

Supplementary Figure 2
Representative images of NanA-RhaPS and IdeS-RhaPS NTA purification. A) Chromatogram of *E. coli* produced NanA-RhaPS (top) and IdeS-RhaPS (bottom) purification *via* an imidazole gradient (orange line). B) Fractions from chromatography shown in the red box in panel (A) were analysed via SDS-PAGE. Two protein species were found in the main fractions, the carrier protein (NanA or IdeS) and the relevant glycoconjugate species.

Supplementary Figure 3
A) NTA column purified IdeS-RhaPS was purified over two rounds of size exclusion chromatography to isolate the higher molecular weight glycosylated IdeS-2x RhaPS and IdeS-RhaPS species. B) Fractions from the second run were analysed via SDS-PAGE and reveal that red-boxed fractions contain primarily the IdeS-2x RhaPS and IdeS-RhaPS glycoconjugate.

Supplementary Figure 4
Monosaccharide composition analysis of IdeS-RhaPS glycoconjugate: The purified glycoconjugate was subjected to methanolysis and trimethylsilylation and the obtained methyl glycosides were analysed by GC-MS; *scyllo*-Inositol (SI) was used as an internal standard. A) ion chromatograms (*m/z* 318 for SI and *m/z* 204 for Rha). Rha: rhamnose, SI: *scyllo*-Inositol. B) MS fragmentation pattern of rhamnose at RT 11.77 min detecting the characteristic ions at *m/z* 204 and *m/z* 217.

Supplementary Figure 5
A)^{1}H NMR spectrum and B) ^{1}H,^{13}C-HSQC NMR spectrum (B) of NanA-RhaPS glycoconjugate.

Supplementary Figure 6
Mass spectrometric analysis reveals that the N-terminal sequons are modified in IdeS and NanA with Shi1-RhaPS. Under the conditions tested, up to 41 rhamnose sugars are identified for IdeS-RhaPS and 36 rhamnoses for NanA. The list of identified glycopeptides and potential glycan modification are shown in Supplementary Data 4, 5 and 6.

Supplementary Figure 7
A) *E. coli* (*rfaS* deletion) cells transformed with a functional RhaPS cluster were grown overnight and subjected to SDS-PAGE and western blotting with three different rabbit

sera. Examples are shown for rabbit 1 and rabbit 3 pre-immune and post NanA-RhaPS immunisation. Sera were diluted 1:3333 and detected with a fluorescent anti-rabbit IgG antibody (1:5,000). B) Anti-NanA-RhaPS IgG antibodies were measured by ELISA using individual rabbit pre-immune and NanA-RhaPS immunised sera from day 35. Data displayed are mean ± SEM from three individual rabbit sera. Unpaired t test analyses (*p<0.05) were performed using GraphPad Prism. C) NanA-RhaPS sera tested against *E. coli* whole cell lysate producing recombinant RhaPS or negative control cells lacking the RhaPS gene cluster.

Supplementary Figure 8
Vaccination with NanA RhaPS results in a significant increase in anti-RhaPS titres. A) Representative ELISA data showing recognition of lipid linked RhaPS (LL-RhaPS) by antiserum from a single rabbit (#1) pre- and post- vaccination with NanA RhaPS. Data are presented as mean ± SD OD_{450} values from three independent assays. B-C) Anti-RhaPS titres were determined pre- and post- NanA RhaPS vaccination using plates coated with LL-RhaPS and a commercially available anti-group A carbohydrate antibody. B) Standard curve generated using anti-group A carbohydrate antibody with an arbitrary tire of 1:12,000. C) Anti-RhaPS titres from three rabbits pre- and post-vaccination with NanA RhaPS. Data are presented as mean ± SD from three independent assays. **** P<0.0001 one-way ANOVA with Tukey's multiple comparison test.

Supplementary Data 1: Proteome database used for Byonic search
Supplementary Data 2: HCD MSMS spectra of identified glycopeptides from NanA and IdeS protein.
Supplementary Data 3: Proteome database used for MSFragger search
Supplementary Data 4: List of identified glycopeptides for NanA and IdeS protein and their relative abundance
Supplementary Data 5: List of C-terminal glycopeptide identified for NanA protein
Supplementary Data 6: List of C-terminal glycopeptide identified for IdeS protein
Supplementary Data 7: CASPER report and NMR chemical shift data

Supplementary Figure 1)

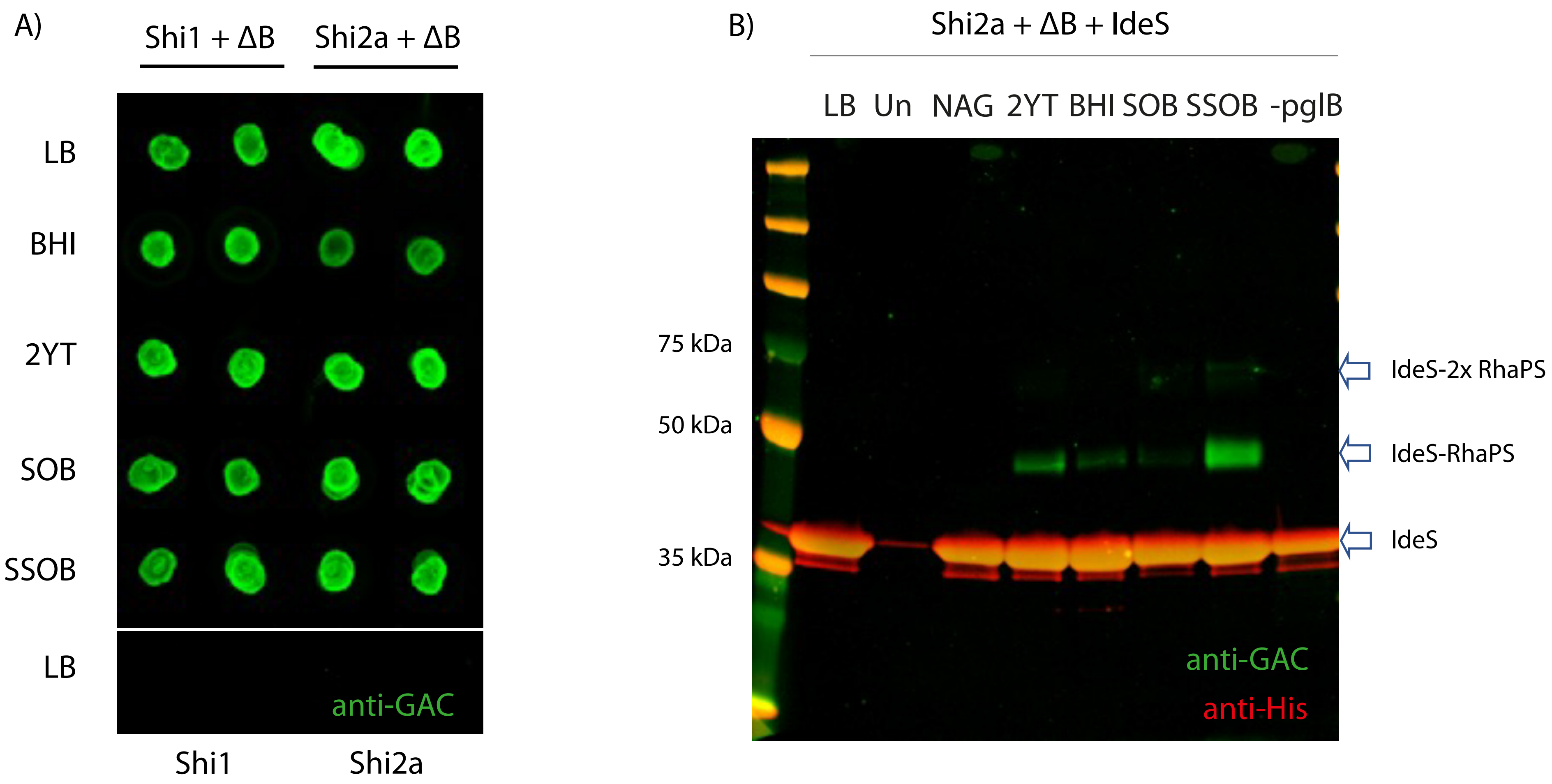

Supplementary Figure 2)

A)

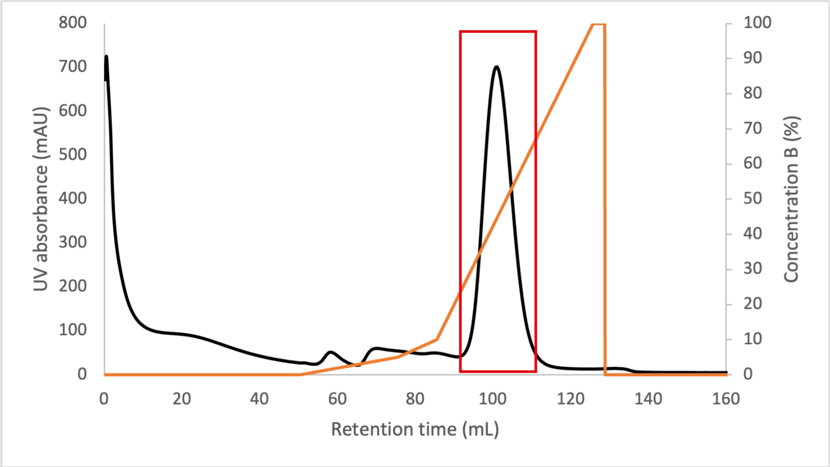

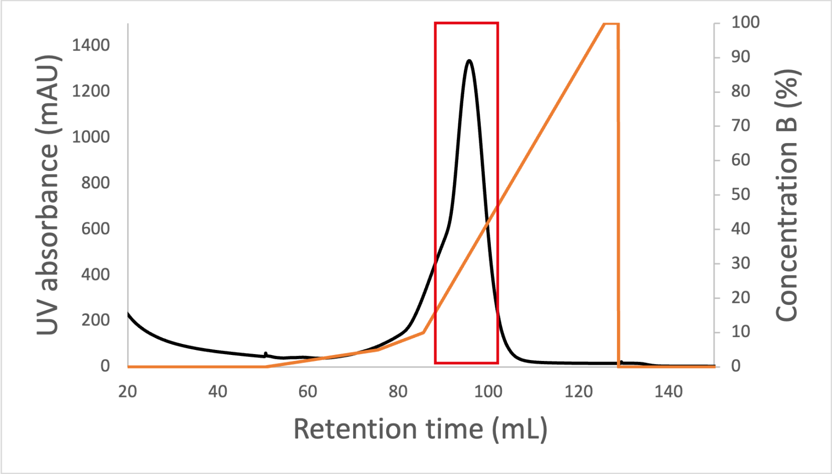

B)

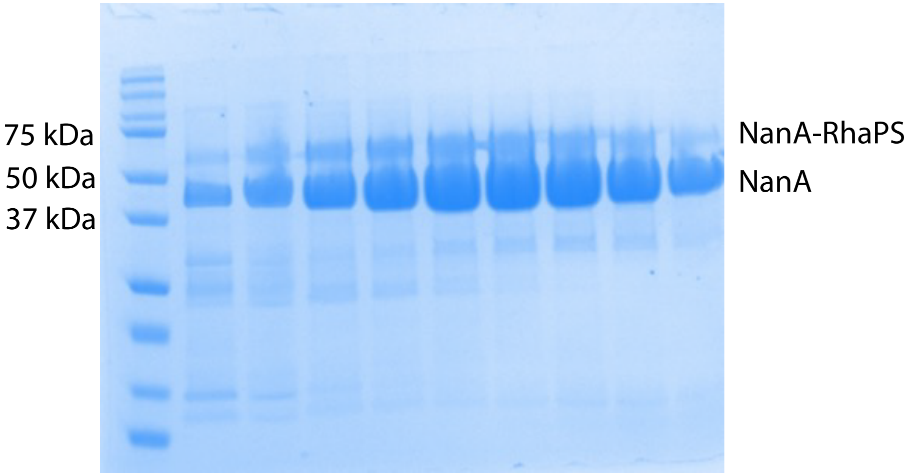

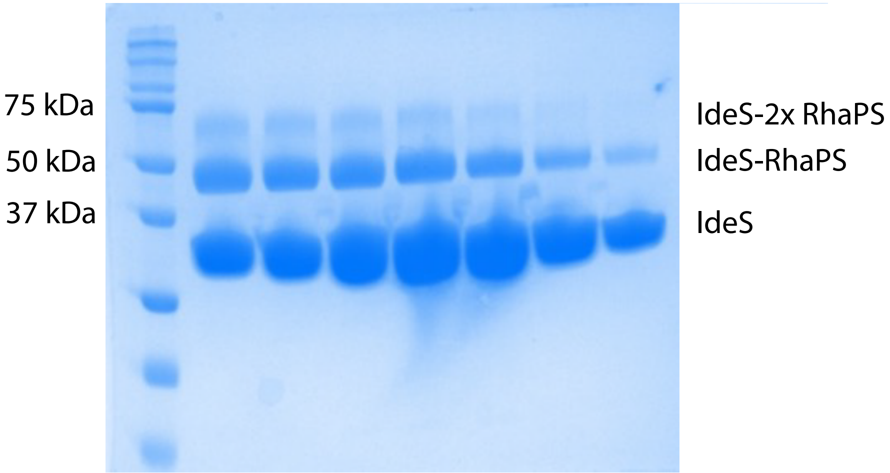

Supplementary Figure 3)

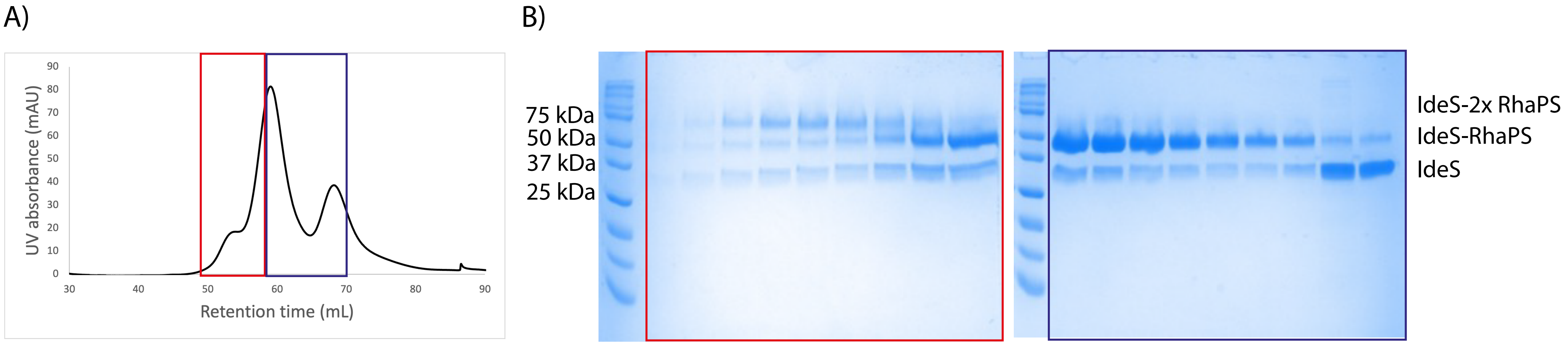

Supplementary Figure 4)

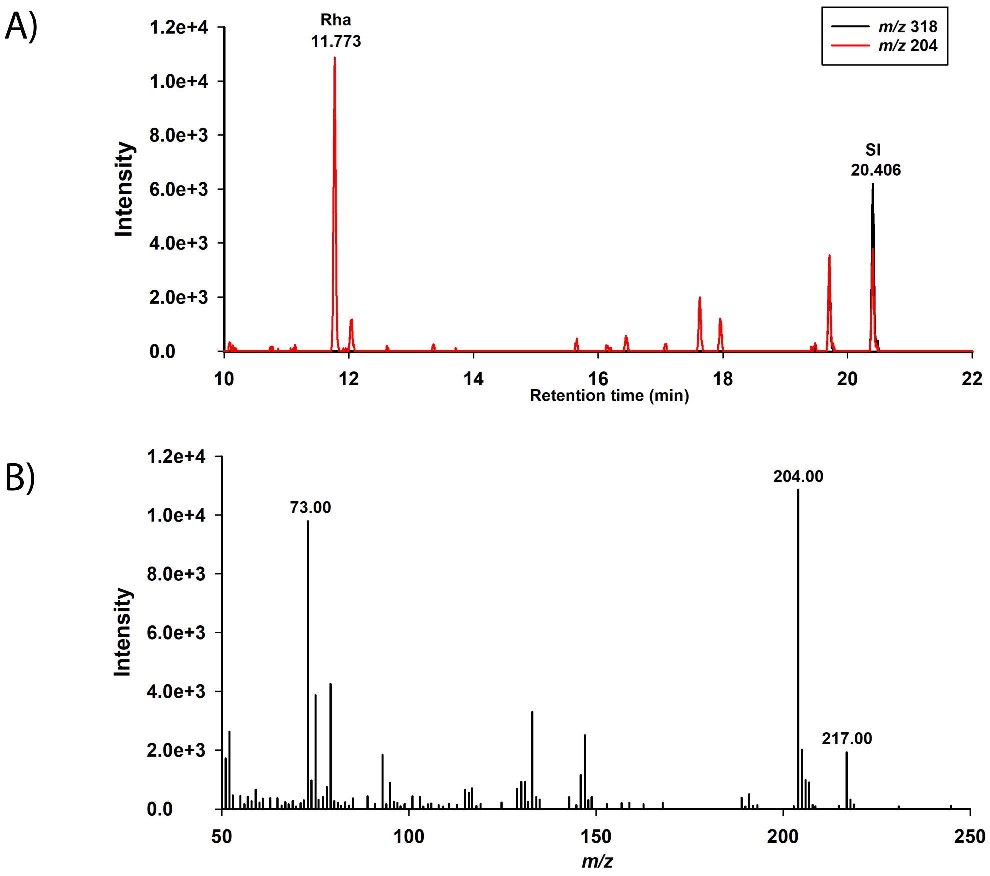

Supplementary Figure 5)

A)

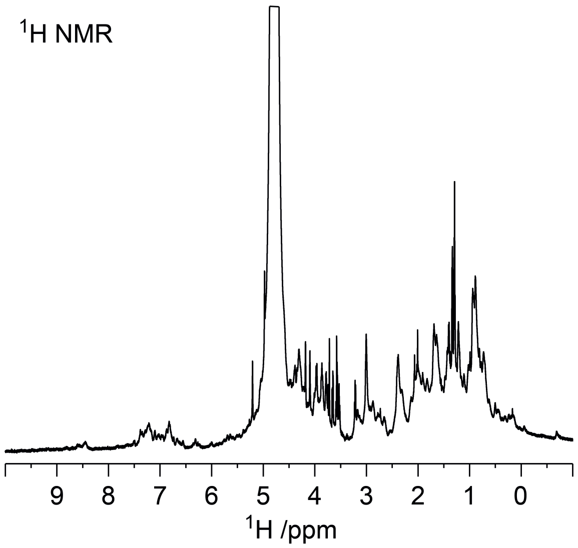

B)

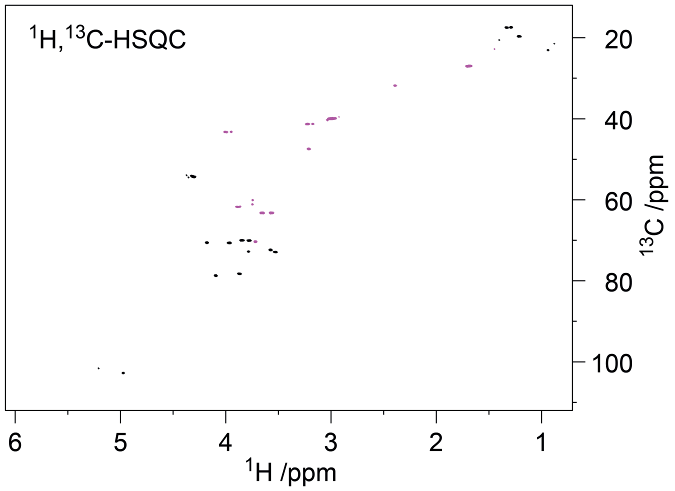

Supplementary Figure 6)

A)

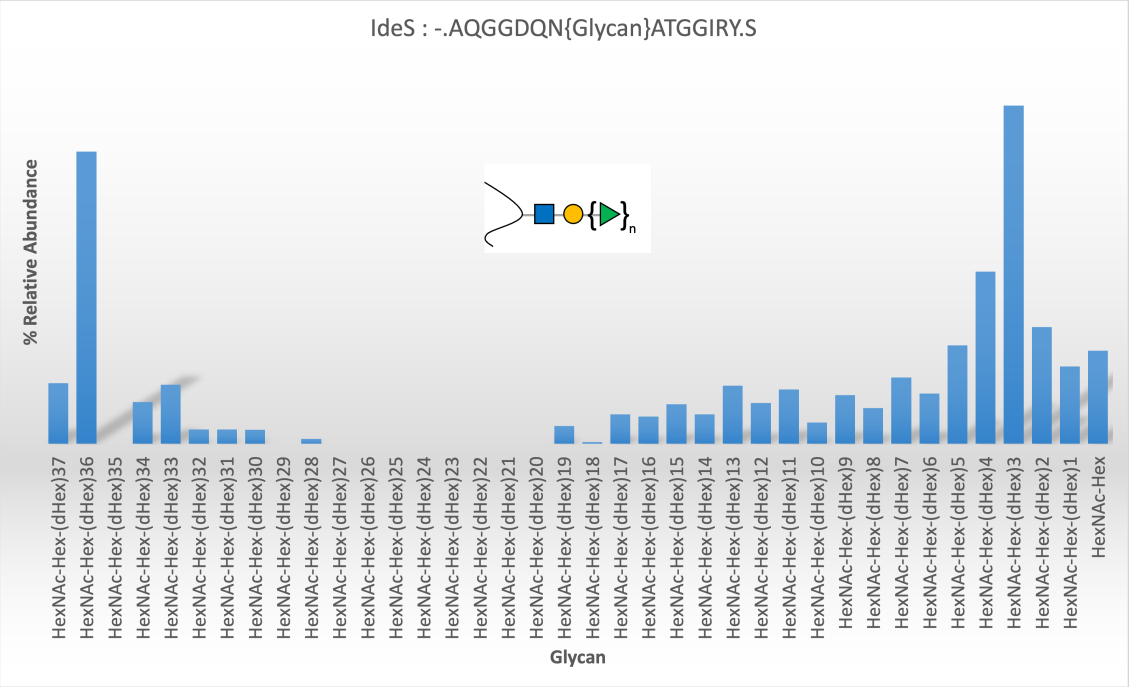

B)

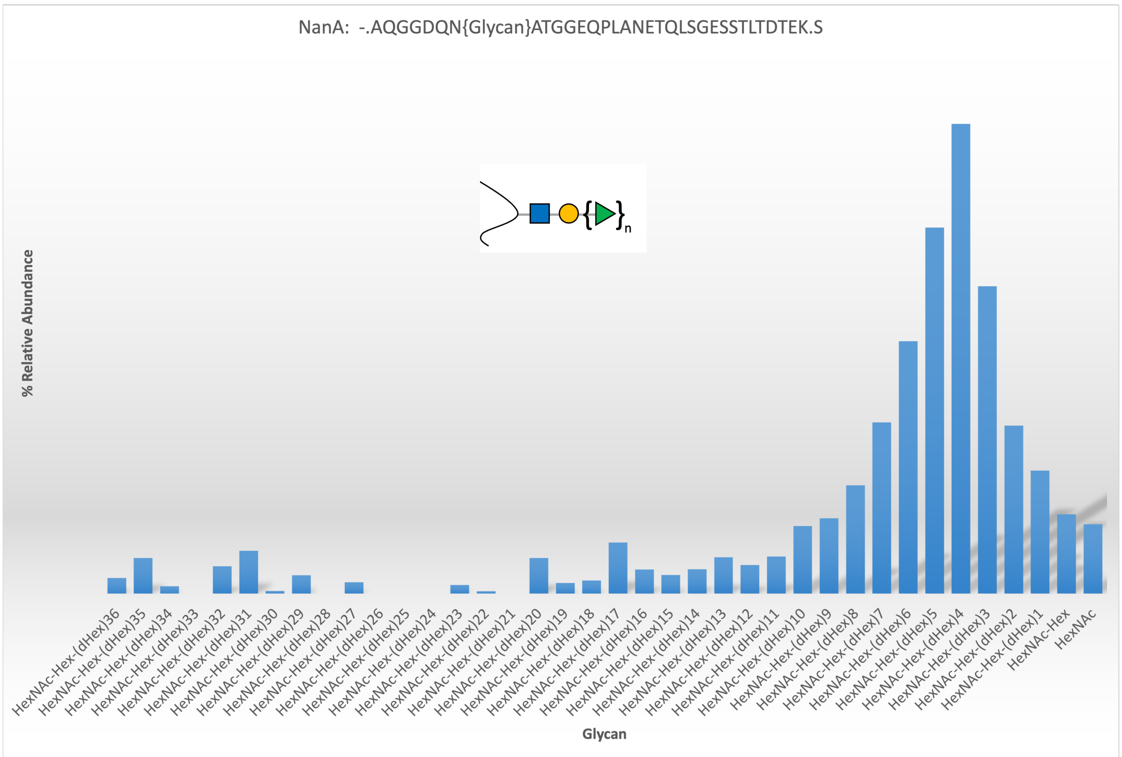

Supplementary Figure 7)

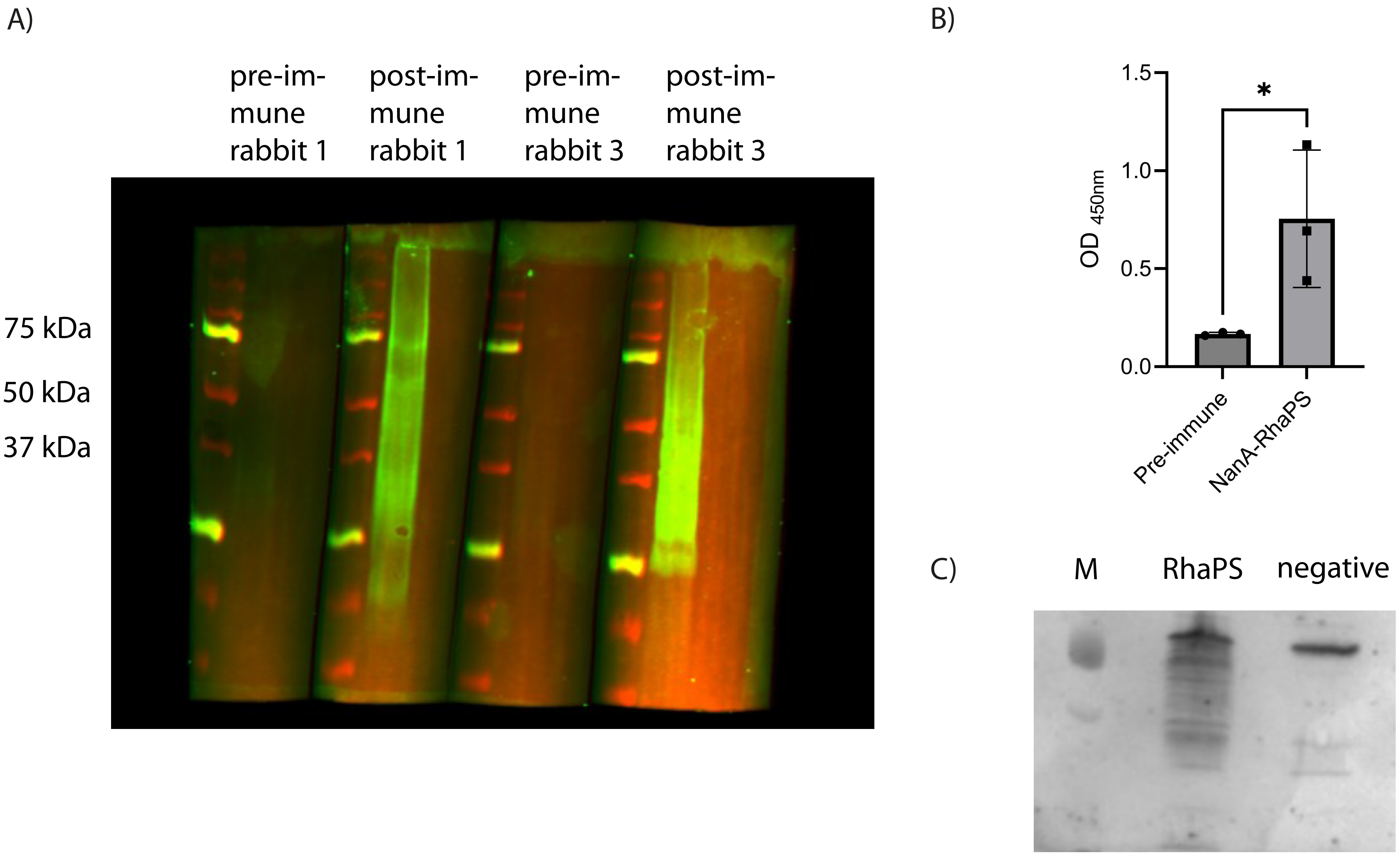

Supplementary Figure 8)

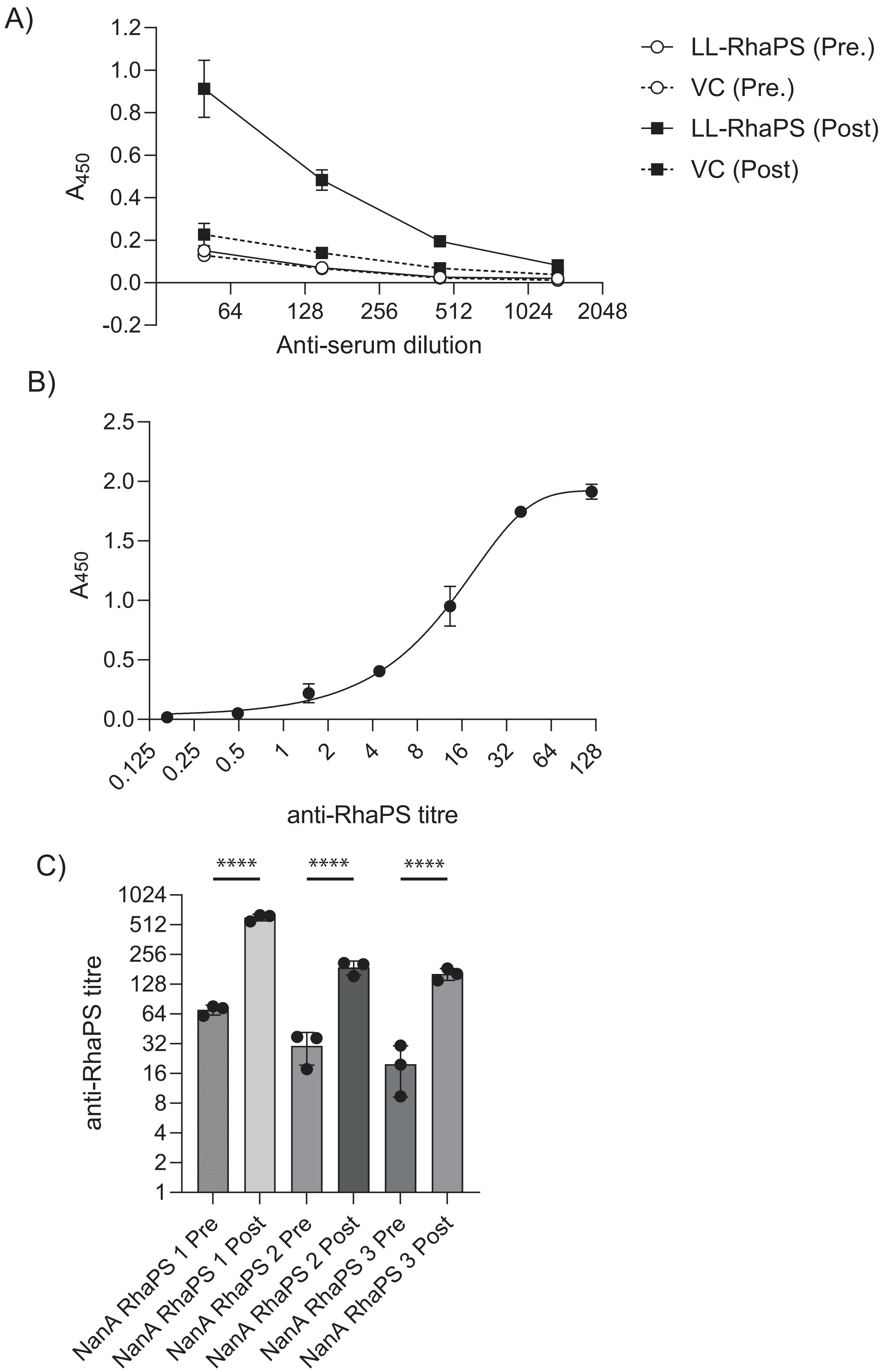

Supplementary Figures - uncropped images from main Figure 2

A)

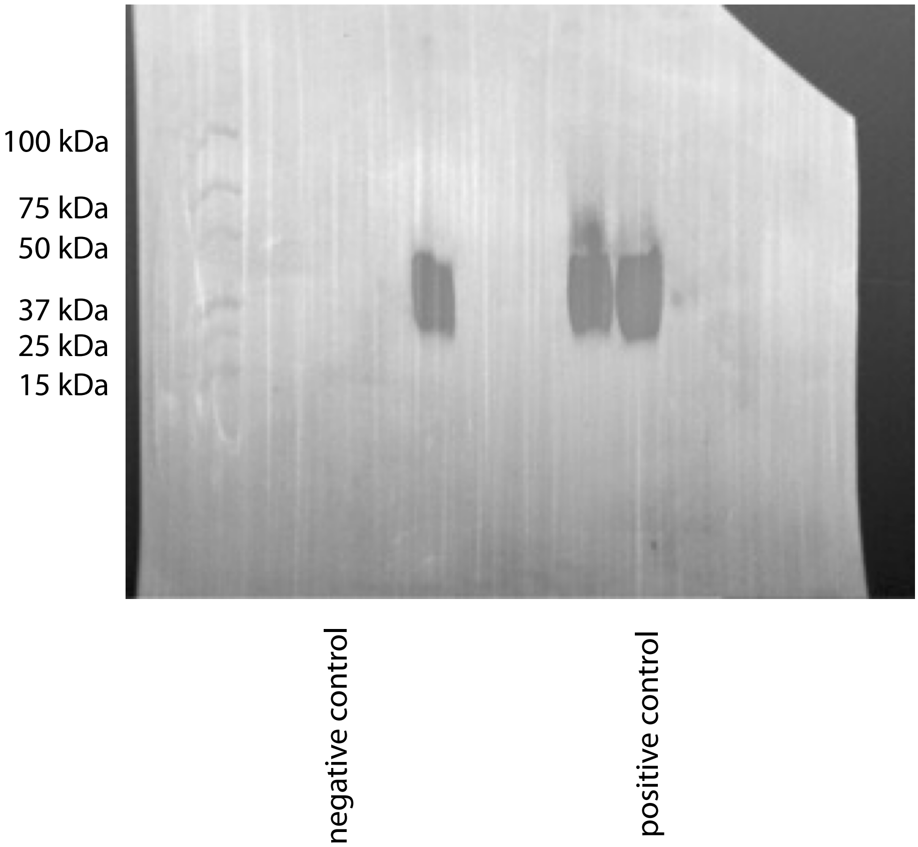

B)

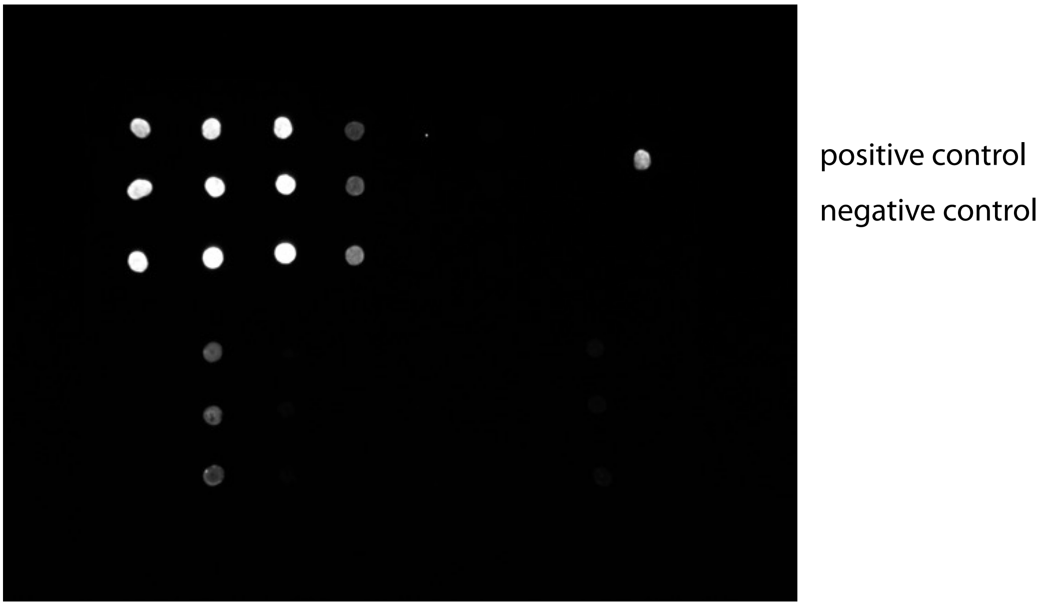

Supplementary Figures - uncropped images from main Figure 3

A)

100 kDa
75 kDa
50 kDa
37 kDa
25 kDa

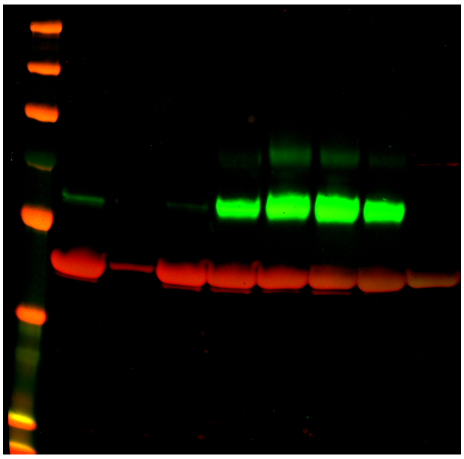

B)

100 kDa
75 kDa
50 kDa
37 kDa
25 kDa

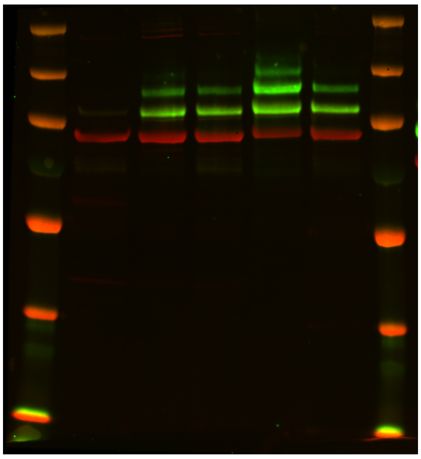

Supplementary Figures - uncropped images from main Figure 4

A)

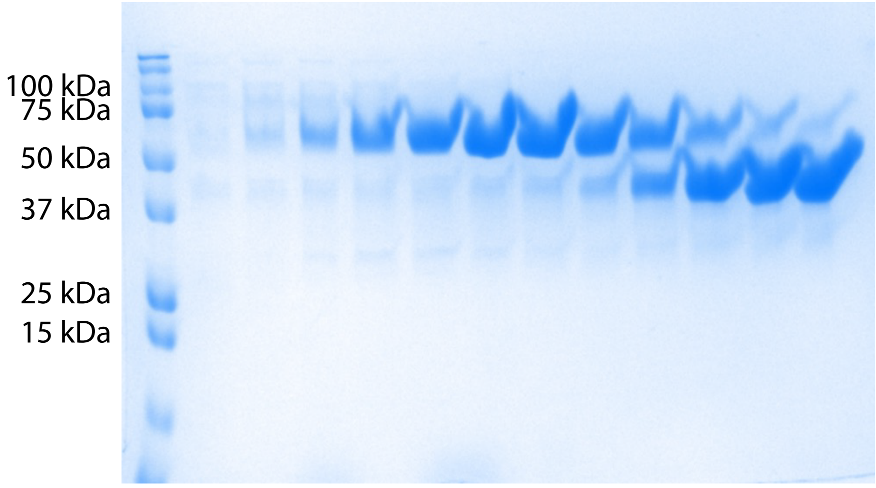

Supplement: Supplementary file 1 — Supplementary information [file 41541_2025_1068_MOESM1_ESM.pdf]
