# Supplementary material for: Recombinant production platform for Group A Streptococcus glycoconjugate vaccines
Source: NPJ Vaccines. 2025 Jan 22;10:16. doi: 10.1038/s41541-025-01068-2 (PMC11754613; doi:10.1038/s41541-025-01068-2)
Supplement: Supplementary file 2 — CASPER and NMR report [file 41541_2025_1068_MOESM2_ESM.pdf]

CASPER report

GP-rhamnan

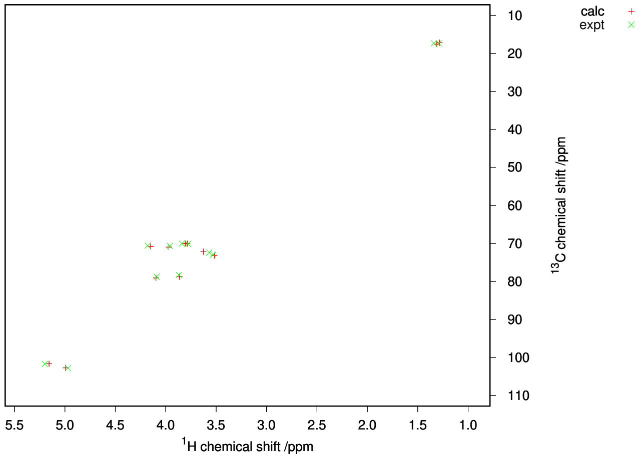

Predicted ^{13}C and ^{1}H NMR chemical shifts

Structure

→2) α-L-Rha^{ii} (1→3) α-L-Rha^{i} (1→

→3) α-L-Rha^{i} (1→	1	2	3	4	5	6
Expected Calc. Error: 3.06	102.76	70.81	78.75	72.19	70.05	17.20
	4.99	4.15	3.86	3.63	3.79	1.28
→2) α-L-Rha^{ii} (1→	1	2	3	4	5	6
Expected Calc. Error: 2.53	101.66	79.09	70.97	73.18	70.02	17.57
	5.16	4.10	3.97	3.52	3.81	1.31

Assignment of ^{13}C, ^{1}H resonances

Experimental	Predicted	Expt-Pred	Assignment
102.82 - 4.97	102.76 - 4.99	0.03	α-L-Rha^{i} - 1
101.70 - 5.20	101.66 - 5.16	0.04	α-L-Rha^{ii} - 1
78.76 - 4.09	79.09 - 4.10	0.07	α-L-Rha^{ii} - 2
78.31 - 3.87	78.75 - 3.86	0.09	α-L-Rha^{i} - 3
72.94 - 3.53	73.18 - 3.52	0.05	α-L-Rha^{ii} - 4
72.40 - 3.57	72.19 - 3.63	0.07	α-L-Rha^{i} - 4
70.67 - 3.96	70.97 - 3.97	0.06	α-L-Rha^{ii} - 3
70.61 - 4.18	70.81 - 4.15	0.05	α-L-Rha^{i} - 2
70.10 - 3.78	70.05 - 3.79	0.01	α-L-Rha^{i} - 5
70.04 - 3.84	70.02 - 3.81	0.03	α-L-Rha^{ii} - 5
17.35 - 1.34	17.57 - 1.31	0.05	α-L-Rha^{ii} - 6
17.38 - 1.29	17.20 - 1.28	0.04	α-L-Rha^{i} - 6
Error=0.59 (0.05/signal) , RMS error=0.05.			

Assignment of long range ^{13}C, ^{1}H correlations

Experimental	Predicted	Expt-Pred	Assignment
102.82 - 4.09	102.76 - 4.10	0.01	α-L-Rha^{i} - 1, α-L-Rha^{ii} - 2
101.70 - 3.87	101.66 - 3.86	0.01	α-L-Rha^{ii} - 1, α-L-Rha^{i} - 3
78.76 - 4.97	79.09 - 4.99	0.07	α-L-Rha^{ii} - 2, α-L-Rha^{i} - 1
78.31 - 5.20	78.75 - 5.16	0.10	α-L-Rha^{i} - 3, α-L-Rha^{ii} - 1
78.31 - 4.97	78.75 - 4.99	0.09	α-L-Rha^{i} - 3, α-L-Rha^{i} - 1
70.67 - 5.20	70.97 - 5.16	0.07	α-L-Rha^{ii} - 3, α-L-Rha^{ii} - 1
70.10 - 4.97	70.05 - 4.99	0.03	α-L-Rha^{i} - 5, α-L-Rha^{i} - 1
70.04 - 5.20	70.02 - 5.16	0.04	α-L-Rha^{ii} - 5, α-L-Rha^{ii} - 1
Error=0.42 (0.05/signal) , RMS error=0.04.			

Assigned experimental ^{13}C and ^{1}H NMR chemical shifts

Structure

→2) α-L-Rha^{ii} (1→3) α-L-Rha^{i} (1→

	1	2	3	4	5	6
→3) α-L-Rha^{i} (1→	1	2	3	4	5	6
^{13}C Error: 1.14. ^{1}H Error: 0.13.	102.82	70.61	78.31	72.40	70.10	17.38
	4.97	4.18	3.87	3.57	3.78	1.29
→2) α-L-Rha^{ii} (1→	1	2	3	4	5	6
^{13}C Error: 1.15. ^{1}H Error: 0.13.	101.70	78.76	70.67	72.94	70.04	17.35
	5.20	4.09	3.96	3.53	3.84	1.34

NMR chemical shift data of GP-rhamnan

HSQC		HMBC	
1H [ppm]	13C [ppm]	1H [ppm]	13C [ppm]
1.34	17.35	5.20	70.04
1.29	17.38	5.20	70.67
3.84	70.04	5.20	78.31
3.78	70.10	4.97	70.10
4.18	70.61	4.97	78.31
3.96	70.67	4.97	78.76
3.57	72.40	4.09	102.82
3.53	72.94	3.87	101.70
3.87	78.31		
4.09	78.76		
5.20	101.70		
4.97	102.82		
		1.34	70.04
		1.34	72.94
		1.29	70.10
		1.29	72.40
