# Supplementary material for: Recombinant production platform for Group A Streptococcus glycoconjugate vaccines
Source: NPJ Vaccines. 2025 Jan 22;10:16. doi: 10.1038/s41541-025-01068-2 (PMC11754613; doi:10.1038/s41541-025-01068-2)
Supplement: Supplementary file 3 — SupplFile.1 [file 41541_2025_1068_MOESM3_ESM.pdf]

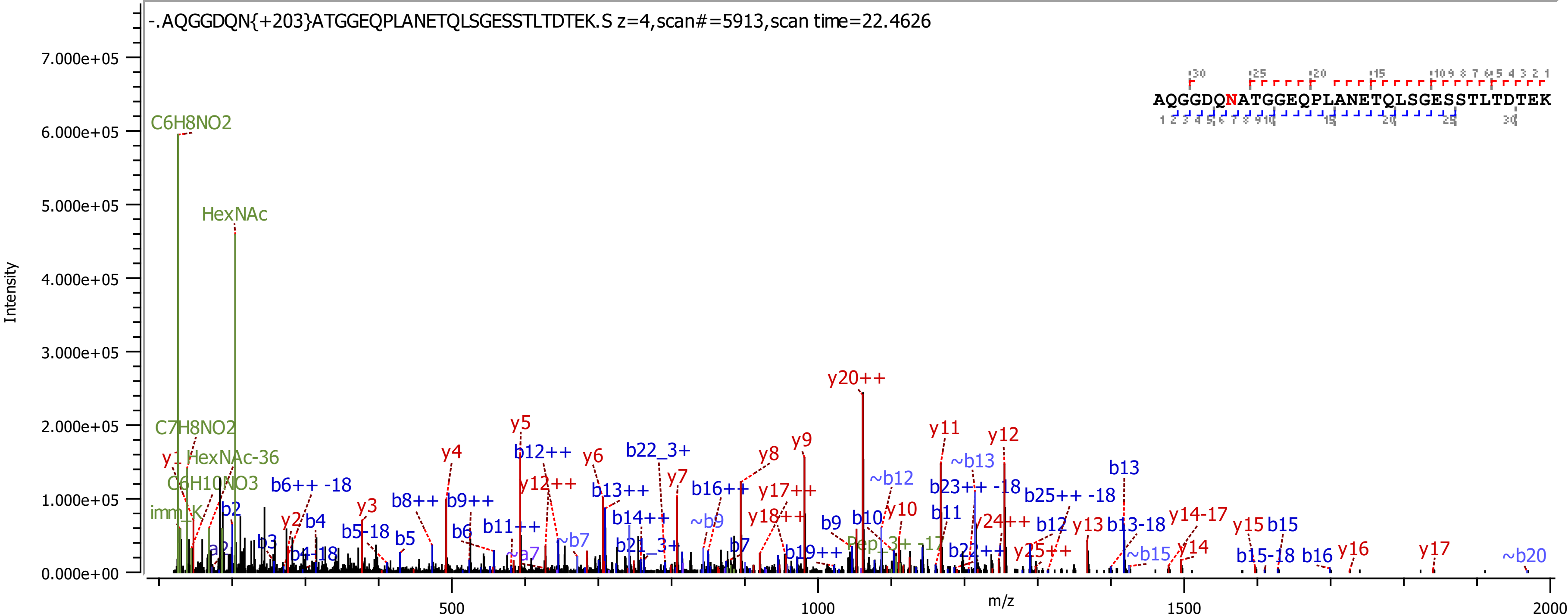
-.AQGGDQN{+203}ATGGEQPLANETQLSGESSTLTDTEK.S z=4,scan#=5913,scan time=22.4626
AQGGDQNATGGEQPLANETQLSGESSTLTDTEK
Intensity
m/z
7.000e+05
6.000e+05
5.000e+05
4.000e+05
3.000e+05
2.000e+05
1.000e+05
0.000e+00
500
1000
1500
2000
C6H8NO2
HexNAc
C7H8NO2
y1
HexNAc-36
C6H10NO3
imm_K
b2
b6++ -18
y20++
y5
y4
b12++
y6
b22_3+
y8
y9
y11
y12
~b13
b13
y12++
y7
b16++
y17++
~b12
b23++ -18
b25++ -18
b13++
b8++
b9++
y3
y2
b4
b14++
y18++
b9
b10
y10
b11
y14-17
b5-18
b6
b11++
~b9
y24++
b12
y13
b13-18
y15
b15
b3
b5
~b7
b7
b21_3+
Pep_3+ -17
y25++
b4-18
~a7
b19++
b22++
~b15
y14
b15-18
b16
y16
y17
~b20

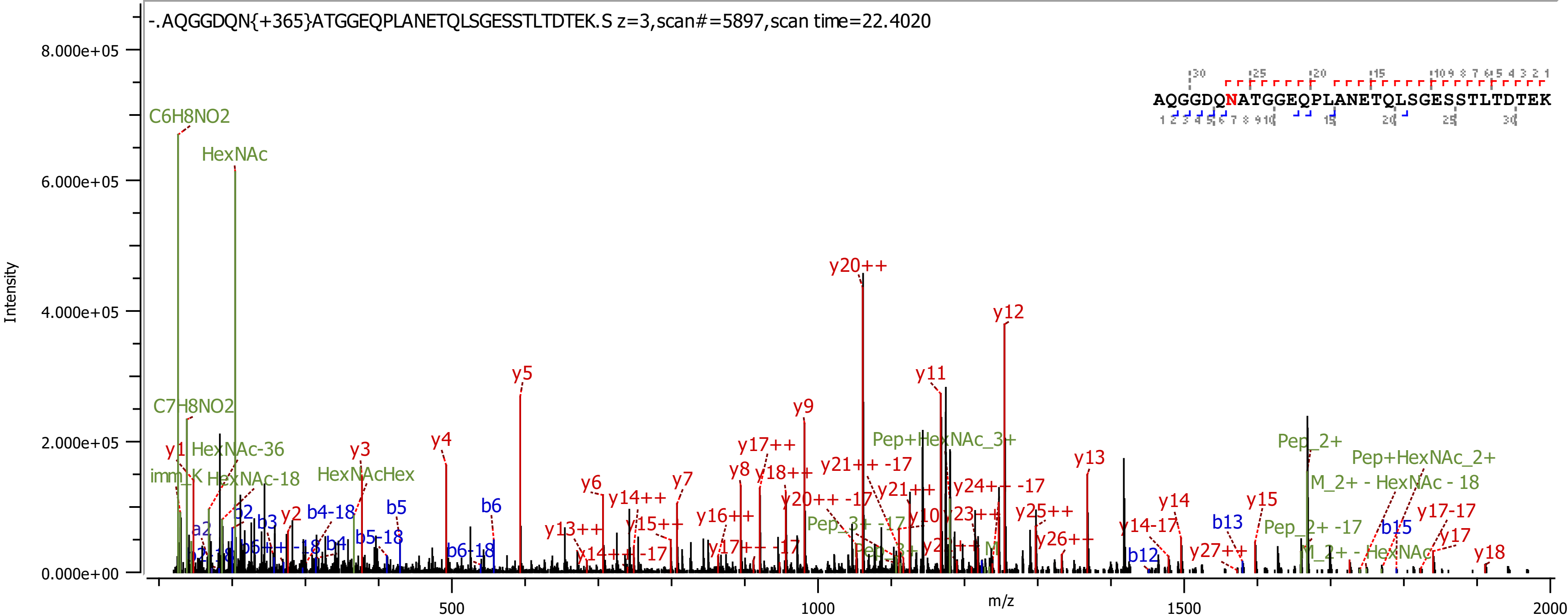

-.AQGGDQN{+365}ATGGEQPLANETQLSGESSTLTDTEK.S z=3,scan#=5897,scan time=22.4020
AQGGDQNATGGEQPLANETQLSGESSTLTDTEK
Intensity
m/z
C6H8NO2
HexNAc
C7H8NO2
y1
HexNAc-36
imm_K
HexNAc-18
HexNAcHex
y3
y4
y5
y6
y7
y8
y9
y11
y12
y13
y14
y15
y17
y18
y20++
b2
b3
b4
b5
b6
b12
b13
b15
Pep+HexNAc_3+
Pep_2+
Pep+HexNAc_2+
M_2+ - HexNAc - 18
M_2+ - HexNAc
0.000e+00
2.000e+05
4.000e+05
6.000e+05
8.000e+05
500
1000
1500
2000

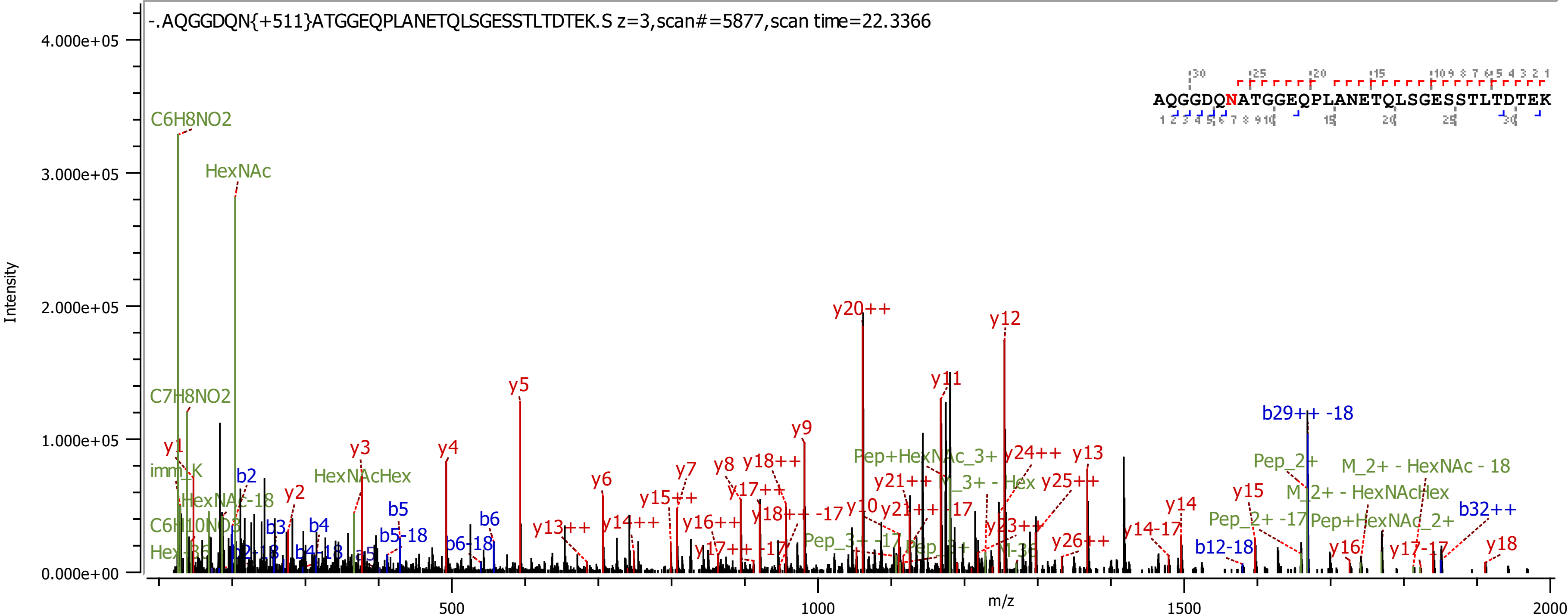
-.AQGGDQN{+511}ATGGEQPLANETQLSGESSTLTDTEK.S z=3,scan#=5877,scan time=22.3366
AQGGDQNATGGEQPLANETQLSGESSTLTDTEK
Intensity
m/z
C6H8NO2
HexNAc
C7H8NO2
imm_K
HexNAc-18
C6H10NO3
HexNAcHex
y20++
y12
y11
y5
y9
y13
b29++ -18
Pep+HexNAc_3+
y24++
y25++
Pep_2+
M_2+ - HexNAc - 18
M_2+ - HexNAcHex
Pep+HexNAc_2+
b32++
y18
y15
y14
y14-17
b12-18
y16
y17-17
y26++
y23++
y1
y2
y3
y4
y6
y7
y8
b2
b3
b4
b5
b5-18
b6
b6-18
a5
y13++
y14++
y15++
y16++
y17++
y18++
y18++ -17
y17++ -17
y10
y21++
y21++ -17
Pep_3+ -17
Pep_2+ -17
0.000e+00
1.000e+05
2.000e+05
3.000e+05
4.000e+05
500
1000
1500
2000

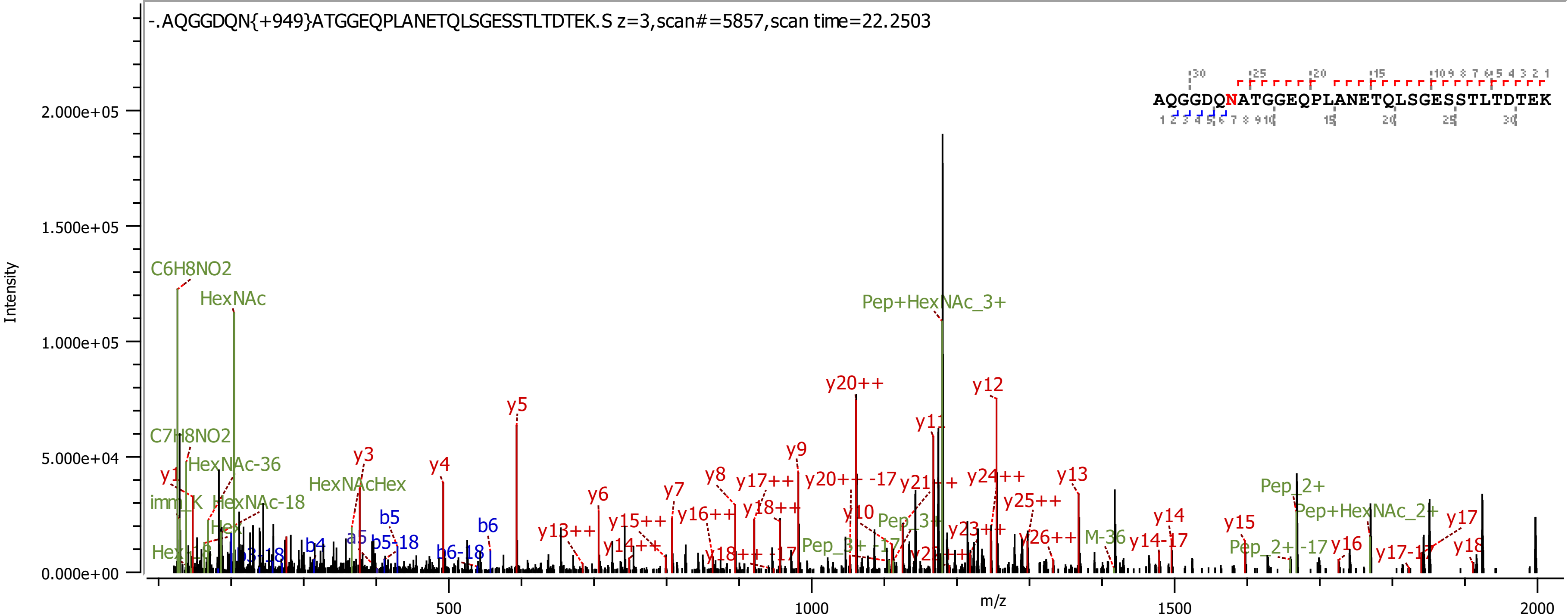
-.AQGGDQN{+949}ATGGEQPLANETQLSGESSTLTDTEK.S z=3,scan#=5857,scan time=22.2503
AQGGDQNATGGEQPLANETQLSGESSTLTDTEK
Intensity
m/z
2.000e+05
1.500e+05
1.000e+05
5.000e+04
0.000e+00
500
1000
1500
2000
C6H8NO2
HexNAc
C7H8NO2
HexNAc-36
HexNAc-18
HexNAcHex
Pep+HexNAc_3+
Pep_2+
Pep+HexNAc_2+
M-36
y20++
y12
y5
y11
y9
y3
y4
y13
y17
y18
y15
y14
y16
b5
b6
b4
a5
b5-18
b6-18

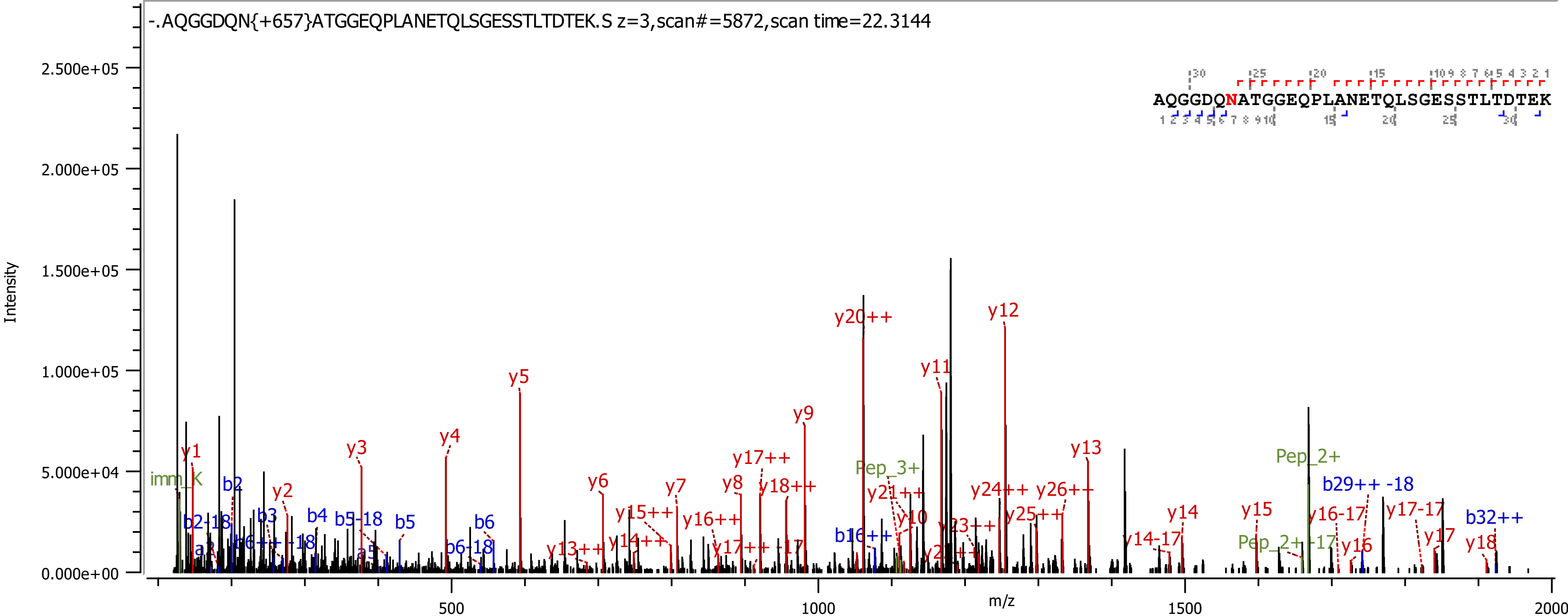
-.AQGGDQN{+657}ATGGEQPLANETQLSGESSTLTDTEK.S z=3,scan#=5872,scan time=22.3144
Intensity
2.500e+05
2.000e+05
1.500e+05
1.000e+05
5.000e+04
0.000e+00
m/z
500
1000
1500
2000
AQGGDQNATGGEQPLANETQLSGESSTLTDTEK
imm_K
y1
b2
b2-18
y2
b3
y3
b4
b5-18
b5
y4
y5
b6
b6-18
y6
y7
y8
y9
y13++
y14++
y15++
y16++
y17++
y17++ -17
y18++
y20++
Pep_3+
y21++
y10
b16++
y11
y12
y23++
y24++
y25++
y26++
y13
y14-17
y14
y15
Pep_2+
Pep_2+ -17
b29++ -18
y16-17
y16
y17-17
y17
b32++
y18

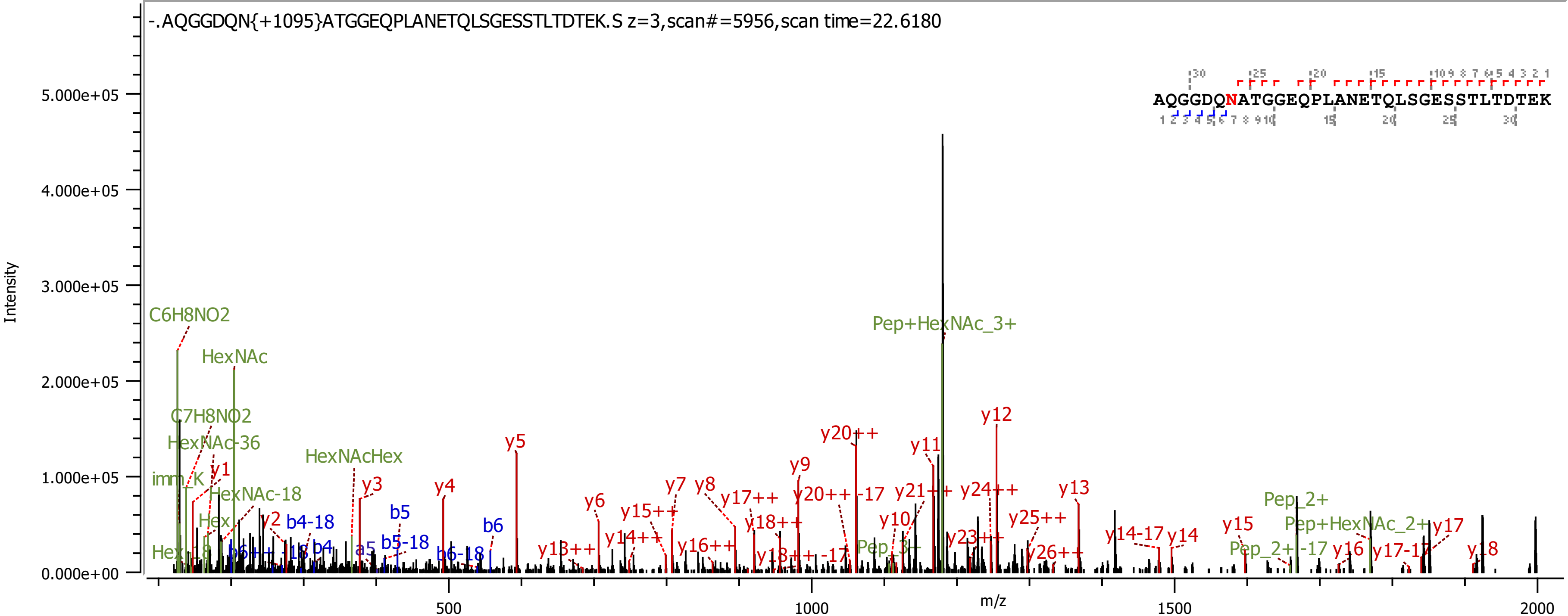

-.AQGGDQN{+1095}ATGGEQPLANETQLSGESSTLTDTEK.S z=3,scan#=5956,scan time=22.6180
AQGGDQNATGGEQPLANETQLSGESSTLTDTEK
Intensity
m/z
5.000e+05
4.000e+05
3.000e+05
2.000e+05
1.000e+05
0.000e+00
500
1000
1500
2000
C6H8NO2
HexNAc
C7H8NO2
HexNAc-36
imm_K
HexNAc-18
HexNAcHex
Hex
Pep+HexNAc_3+
Pep_2+
Pep+HexNAc_2+
Pep_2+-17
Pep_3+
y1
y2
y3
y4
y5
y6
y7
y8
y9
y10
y11
y12
y13
y14
y14-17
y15
y16
y17
y17-17
y18
y13++
y14++
y15++
y16++
y17++
y18++
y18++-17
y20++
y20++-17
y21++
y23++
y24++
y25++
y26++
b4
b4-18
b5
b5-18
b6
b6-18
a5

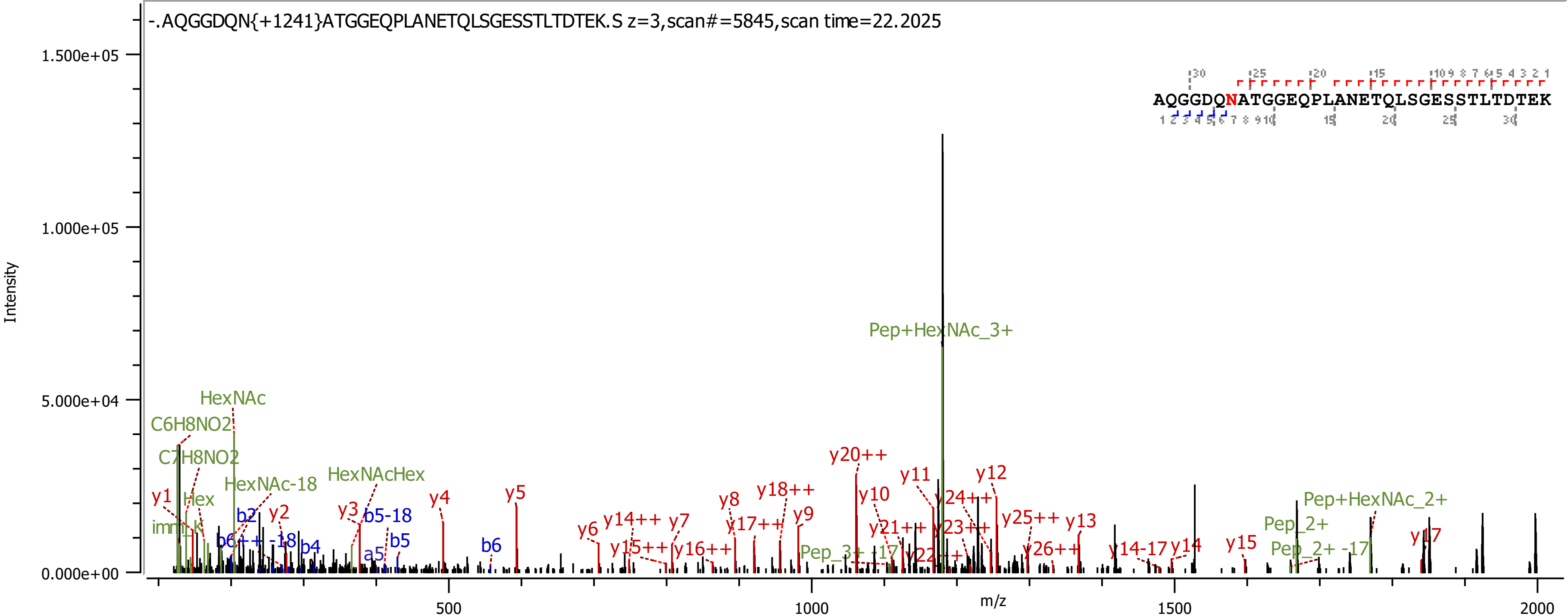
-.AQGGDQN{+1241}ATGGEQPLANETQLSGESSTLTDTEK.S z=3,scan#=5845,scan time=22.2025
AQGGDQNATGGEQPLANETQLSGESSTLTDTEK
Intensity
m/z
1.500e+05
1.000e+05
5.000e+04
0.000e+00
500
1000
1500
2000
Pep+HexNAc_3+
HexNAc
C6H8NO2
C7H8NO2
HexNAc-18
HexNAcHex
Hex
imm_K
y1
y2
y3
y4
y5
y6
y7
y8
y9
y10
y11
y12
y13
y14
y15
y17
y14-17
y14++
y15++
y16++
y17++
y18++
y20++
y21++
y22++
y23++
y24++
y25++
y26++
b2
b4
b5
b6
b5-18
b6++ -18
a5
Pep_3+ -17
Pep_2+
Pep_2+ -17
Pep+HexNAc_2+

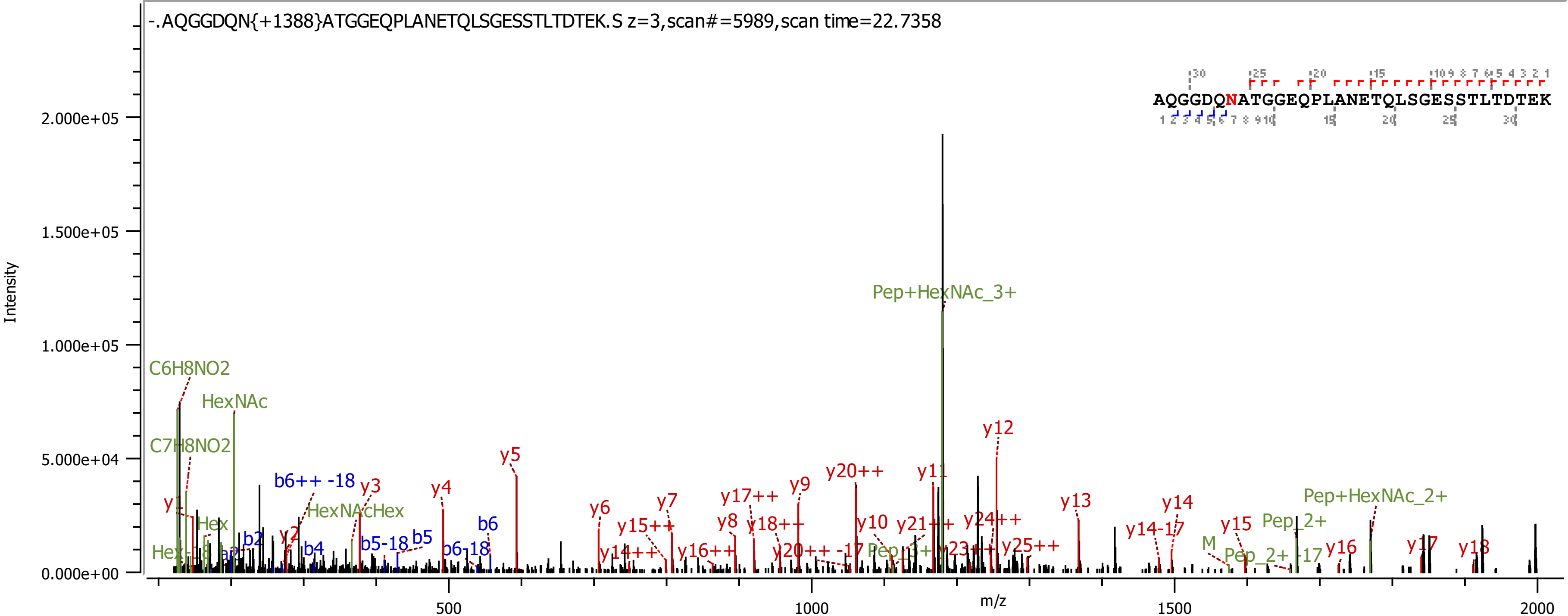

-.AQGGDQN{+1388}ATGGEQPLANETQLSGESSTLTDTEK.S z=3,scan#=5989,scan time=22.7358
AQGGDQNATGGEQPLANETQLSGESSTLTDTEK
Intensity
m/z
2.000e+05
1.500e+05
1.000e+05
5.000e+04
0.000e+00
500
1000
1500
2000
Pep+HexNAc_3+
C6H8NO2
HexNAc
C7H8NO2
Hex
HexNAcHex
b6++ -18
b2
b4
b5-18
b5
b6
b6-18
y2
y3
y4
y5
y6
y7
y8
y9
y10
y11
y12
y13
y14
y14-17
y15
y16
y17
y18
y14++
y15++
y16++
y17++
y18++
y20++
y20++ -17
y21++
y23++
y24++
y25++
Pep_3+
Pep_2+
Pep_2+ -17
Pep+HexNAc_2+
M

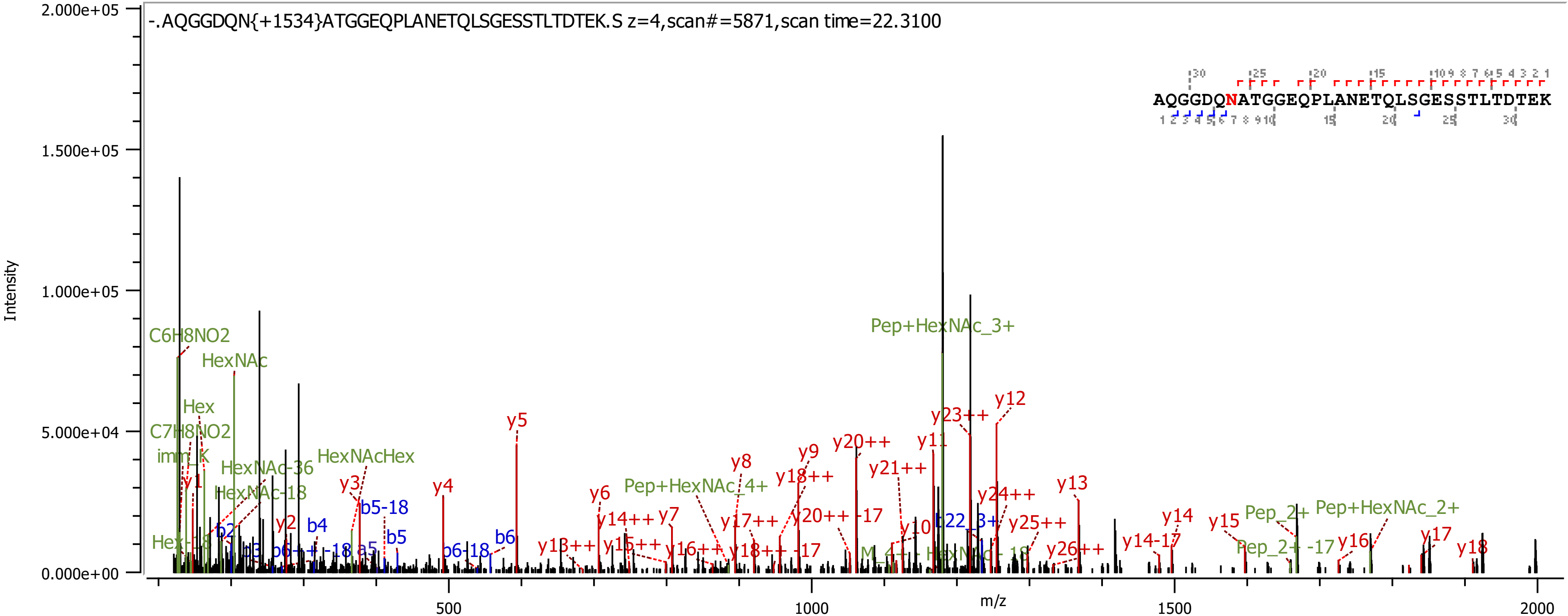
-.AQGGDQN{+1534}ATGGEQPLANETQLSGESSTLTDTEK.S z=4,scan#=5871,scan time=22.3100
AQGGDQNATGGEQPLANETQLSGESSTLTDTEK
Intensity
m/z
2.000e+05
1.500e+05
1.000e+05
5.000e+04
0.000e+00
500
1000
1500
2000
C6H8NO2
HexNAc
Hex
C7H8NO2
HexNAc-36
HexNAc-18
HexNAcHex
Pep+HexNAc_3+
Pep+HexNAc_4+
Pep+HexNAc_2+
Pep_2+
Pep_2+ -17
y1
y2
y3
y4
y5
y6
y7
y8
y9
y10
y11
y12
y13
y14
y14-17
y15
y16
y17
y18
y14++
y15++
y16++
y17++
y18++
y18++ -17
y20++
y20++ -17
y21++
y23++
y24++
y25++
y26++
b2
b3
b4
b5
b5-18
b6
b6-18
a5
b22_3+

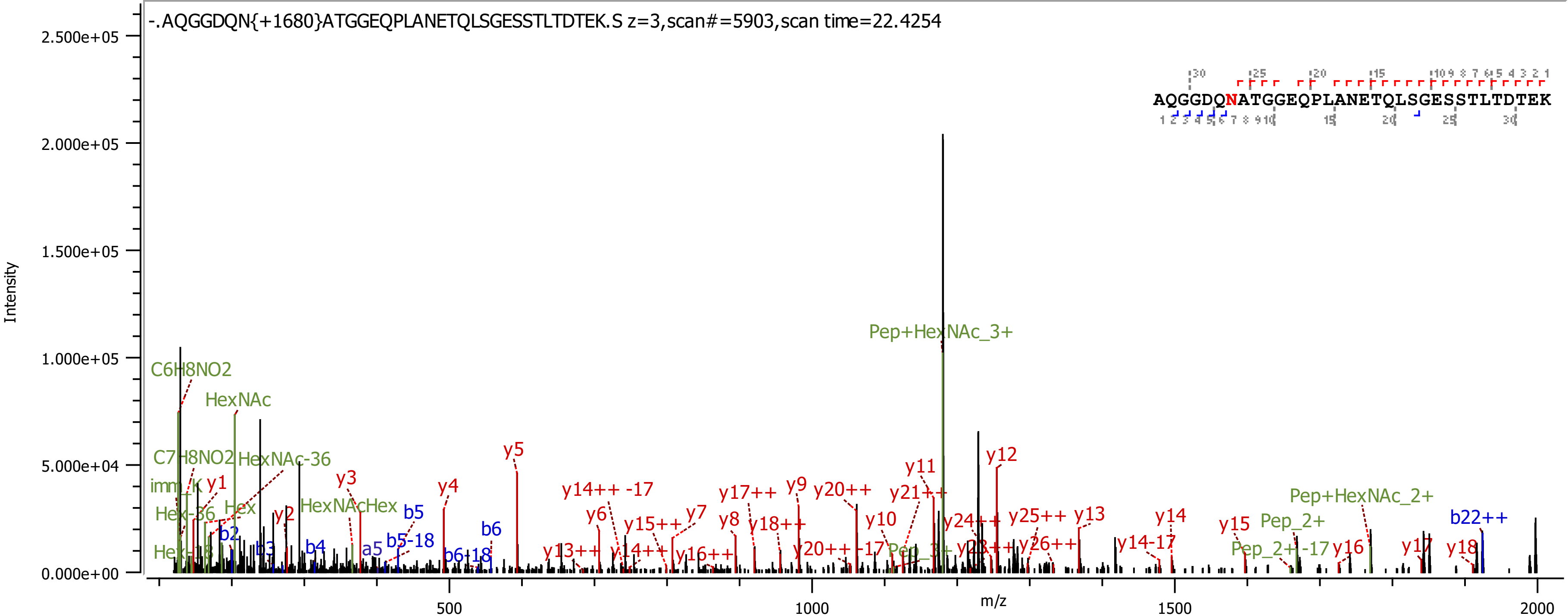

-.AQGGDQN{+1680}ATGGEQPLANETQLSGESSTLTDTEK.S z=3,scan#=5903,scan time=22.4254
AQGGDQNATGGEQPLANETQLSGESSTLTDTEK
Intensity
m/z
2.500e+05
2.000e+05
1.500e+05
1.000e+05
5.000e+04
0.000e+00
500
1000
1500
2000
C6H8NO2
HexNAc
C7H8NO2
HexNAc-36
imm_K
Hex-36
Hex
Hex-18
HexNAcHex
Pep+HexNAc_3+
Pep+HexNAc_2+
Pep_2+
Pep_2+-17
y1
y2
y3
y4
y5
y6
y7
y8
y9
y10
y11
y12
y13
y14
y15
y16
y17
y18
y14++ -17
y13++
y14++
y15++
y16++
y17++
y18++
y20++
y20++-17
y21++
y23++
y24++
y25++
y26++
y14-17
b2
b3
b4
b5
b5-18
a5
b6
b6-18
b22++

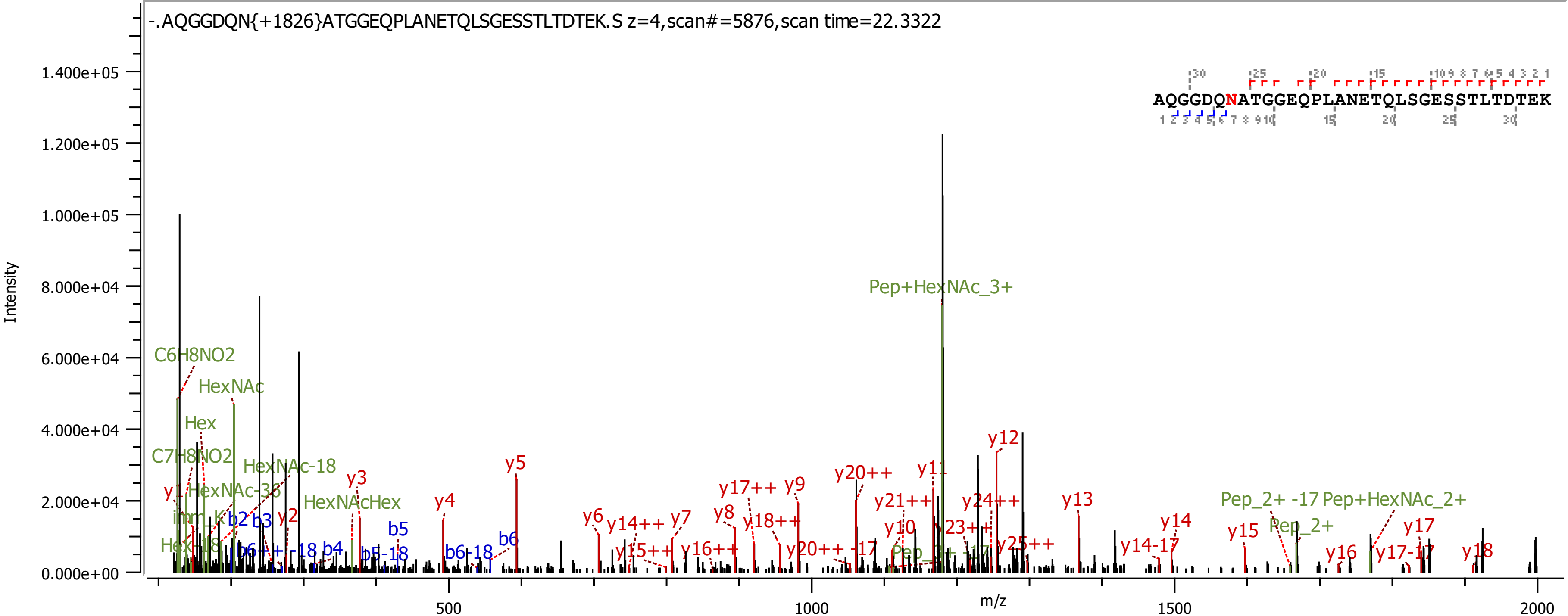

-.AQGGDQN{+1826}ATGGEQPLANETQLSGESSTLTDTEK.S z=4,scan#=5876,scan time=22.3322
AQGGDQNATGGEQPLANETQLSGESSTLTDTEK
Intensity
m/z
1.400e+05
1.200e+05
1.000e+05
8.000e+04
6.000e+04
4.000e+04
2.000e+04
0.000e+00
500
1000
1500
2000
Pep+HexNAc_3+
C6H8NO2
HexNAc
Hex
C7H8NO2
HexNAc-18
HexNAc-36
HexNAcHex
Pep_2+ -17
Pep+HexNAc_2+
Pep_2+
y3
y5
y4
y2
b2
b3
b4
b5
b6
b5-18
b6-18
y6
y7
y8
y9
y10
y11
y12
y13
y14
y14-17
y15
y16
y17
y17-17
y18
y14++
y15++
y16++
y17++
y18++
y20++
y20++ -17
y21++
y23++
y24++
y25++

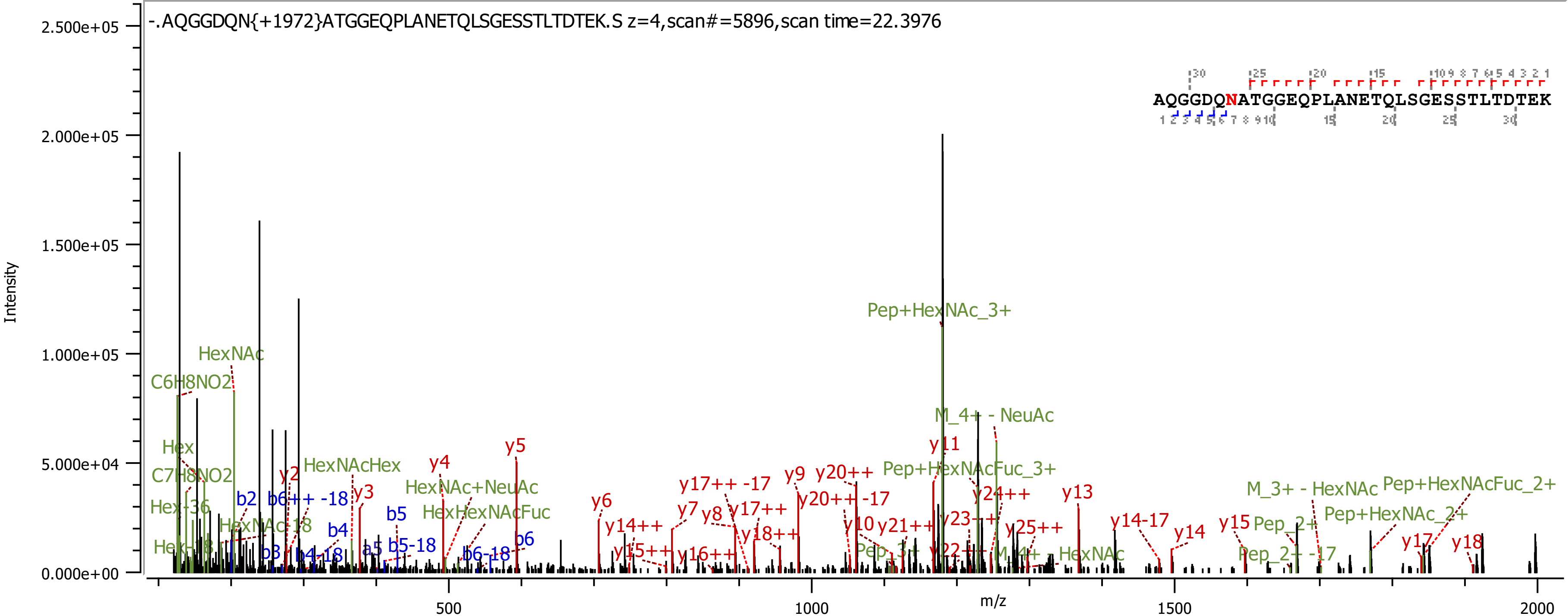
-.AQGGDQN{+1972}ATGGEQPLANETQLSGESSTLTDTEK.S z=4,scan#=5896,scan time=22.3976
AQGGDQNATGGEQPLANETQLSGESSTLTDTEK
Intensity
m/z
2.500e+05
2.000e+05
1.500e+05
1.000e+05
5.000e+04
0.000e+00
500
1000
1500
2000
HexNAc
C6H8NO2
Hex
C7H8NO2
Hex-36
Hex-18
HexNAc-18
HexNAcHex
HexNAc+NeuAc
HexHexNAcFuc
b2
b6++ -18
b3
b4
b4-18
b5
b5-18
a5
b6
b6-18
y2
y3
y4
y5
y6
y7
y8
y9
y10
y11
y13
y14
y14-17
y15
y17
y18
y14++
y15++
y16++
y17++
y17++ -17
y18++
y20++
y20++ -17
y21++
y22++
y23++
y24++
y25++
Pep+HexNAc_3+
M_4+ - NeuAc
Pep+HexNAcFuc_3+
Pep_3+
M_4+ - HexNAc
M_3+ - HexNAc
Pep+HexNAcFuc_2+
Pep+HexNAc_2+
Pep_2+
Pep_2+ -17

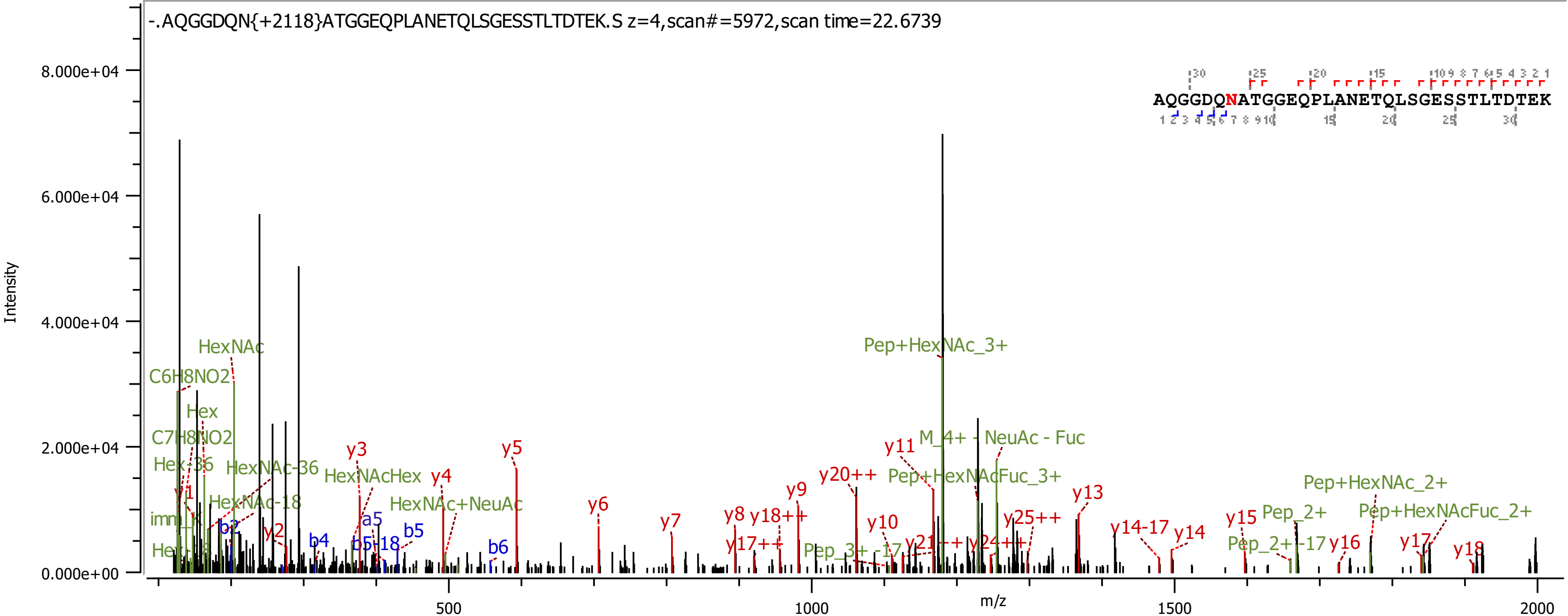

-.AQGGDQN{+2118}ATGGEQPLANETQLSGESSTLTDTEK.S z=4,scan#=5972,scan time=22.6739
AQGGDQNATGGEQPLANETQLSGESSTLTDTEK
Intensity
m/z
8.000e+04
6.000e+04
4.000e+04
2.000e+04
0.000e+00
500
1000
1500
2000
HexNAc
C6H8NO2
Hex
C7H8NO2
Hex-36
HexNAc-36
HexNAc-18
HexNAcHex
HexNAc+NeuAc
imm_K
y1
y2
y3
y4
y5
y6
y7
y8
y9
y10
y11
y13
y14
y14-17
y15
y16
y17
y18
y17++
y18++
y20++
y21++
y24++
y25++
b2
b4
b5
b5-18
b6
a5
Pep+HexNAc_3+
Pep+HexNAcFuc_3+
M_4+ - NeuAc - Fuc
Pep_3+ -17
Pep_2+
Pep_2+ -17
Pep+HexNAc_2+
Pep+HexNAcFuc_2+

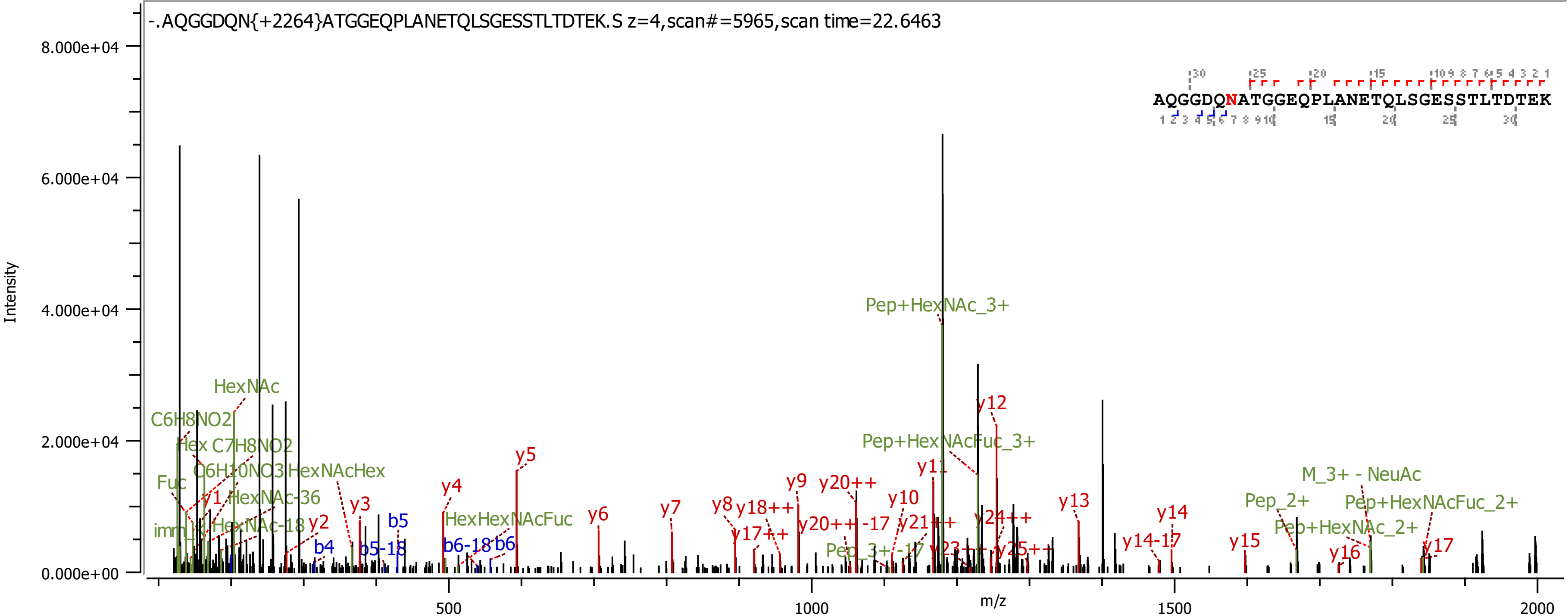
-.AQGGDQN{+2264}ATGGEQPLANETQLSGESSTLTDTEK.S z=4,scan#=5965,scan time=22.6463
AQGGDQNATGGEQPLANETQLSGESSTLTDTEK
Intensity
m/z
Pep+HexNAc_3+
Pep+HexNAcFuc_3+
M_3+ - NeuAc
Pep_2+
Pep+HexNAcFuc_2+
Pep+HexNAc_2+
HexNAc
C6H8NO2
Hex C7H8NO2
C6H10NO3 HexNAcHex
Fuc
y1 HexNAc-36
HexNAc-18
HexHexNAcFuc
y2 y3 y4 y5 y6 y7 y8 y9 y10 y11 y12 y13 y14 y15 y16 y17
y14-17
y17++ y18++ y20++ y20++-17 y21++ y23++ y24++ y25++
b4 b5 b5-18 b6-18 b6
Pep_3+-17

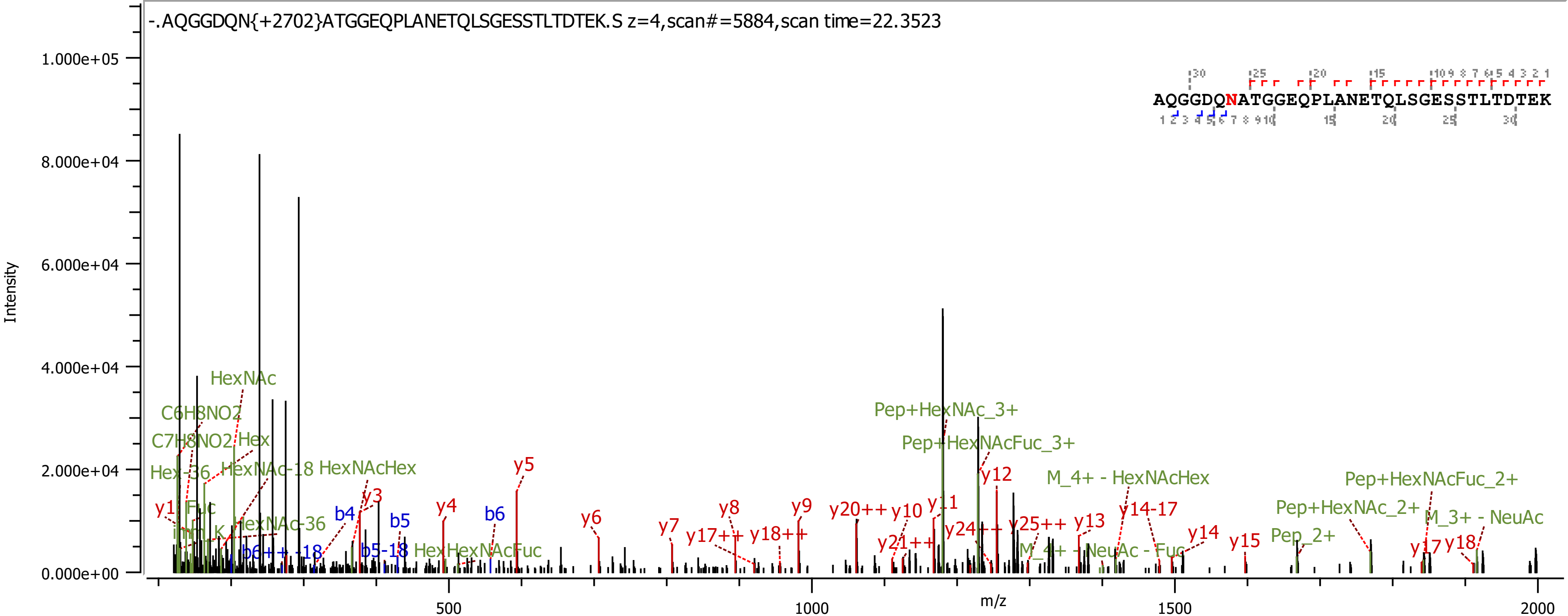
-.AQGGDQN{+2702}ATGGEQPLANETQLSGESSTLTDTEK.S z=4,scan#=5884,scan time=22.3523
AQGGDQNATGGEQPLANETQLSGESSTLTDTEK
Intensity
m/z
0.000e+00
2.000e+04
4.000e+04
6.000e+04
8.000e+04
1.000e+05
500
1000
1500
2000
HexNAc
C6H8NO2
C7H8NO2
Hex
Hex-36
HexNAc-18
HexNAcHex
HexNAc-36
Fuc
y1
y3
b4
b5
b6
y4
y5
y6
y7
y8
y9
b5-18
b6++ -18
HexHexNAcFuc
y17++
y18++
y20++
y10
y11
y21++
y24++
y25++
y12
y13
y14-17
y14
y15
y17
y18
Pep+HexNAc_3+
Pep+HexNAcFuc_3+
M_4+ - HexNAcHex
M_4+ - NeuAc - Fuc
Pep_2+
Pep+HexNAc_2+
Pep+HexNAcFuc_2+
M_3+ - NeuAc

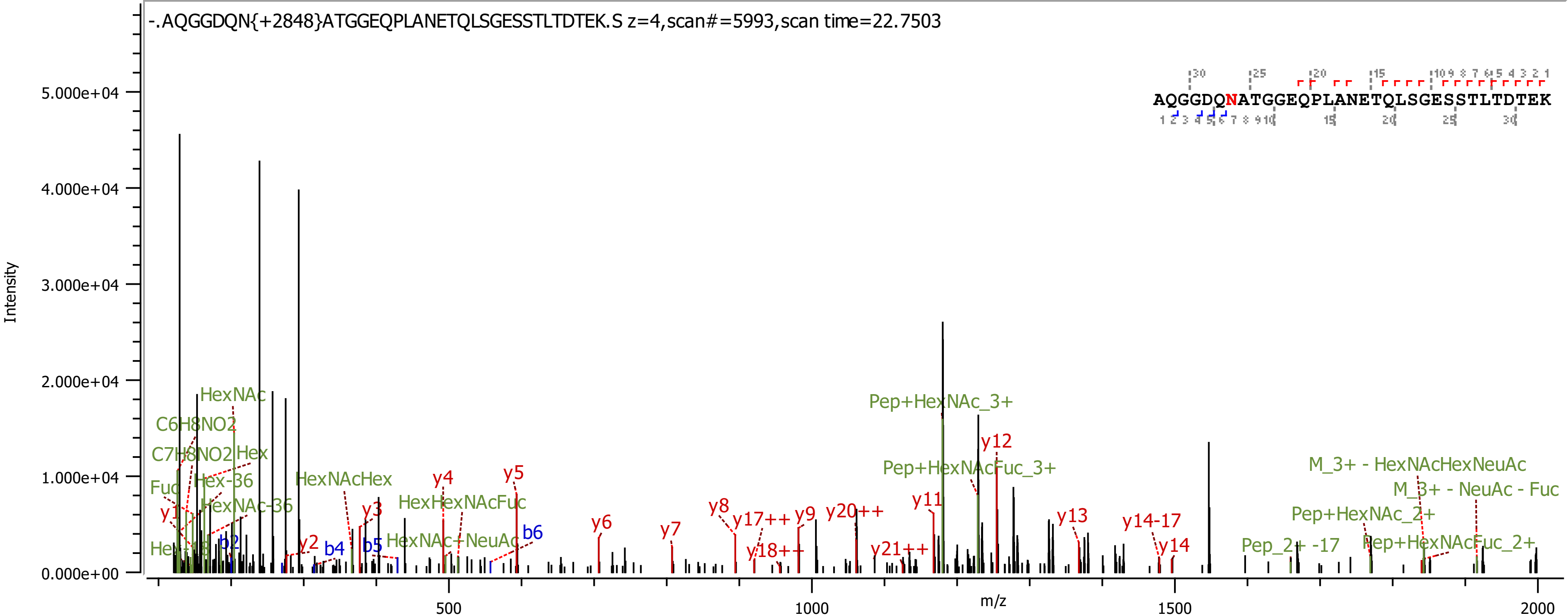
-.AQGGDQN{+2848}ATGGEQPLANETQLSGESSTLTDTEK.S z=4,scan#=5993,scan time=22.7503
AQGGDQNATGGEQPLANETQLSGESSTLTDTEK
Intensity
m/z
HexNAc
C6H8NO2
C7H8NO2
Hex
Fuc
Hex-36
HexNAc-36
HexNAcHex
HexHexNAcFuc
HexNAc+NeuAc
Pep+HexNAc_3+
Pep+HexNAcFuc_3+
M_3+ - HexNAcHexNeuAc
M_3+ - NeuAc - Fuc
Pep+HexNAc_2+
Pep_2+ -17
Pep+HexNAcFuc_2+
y1
y2
y3
y4
y5
y6
y7
y8
y9
y11
y12
y13
y14
y14-17
y17++
y18++
y20++
y21++
b2
b4
b5
b6

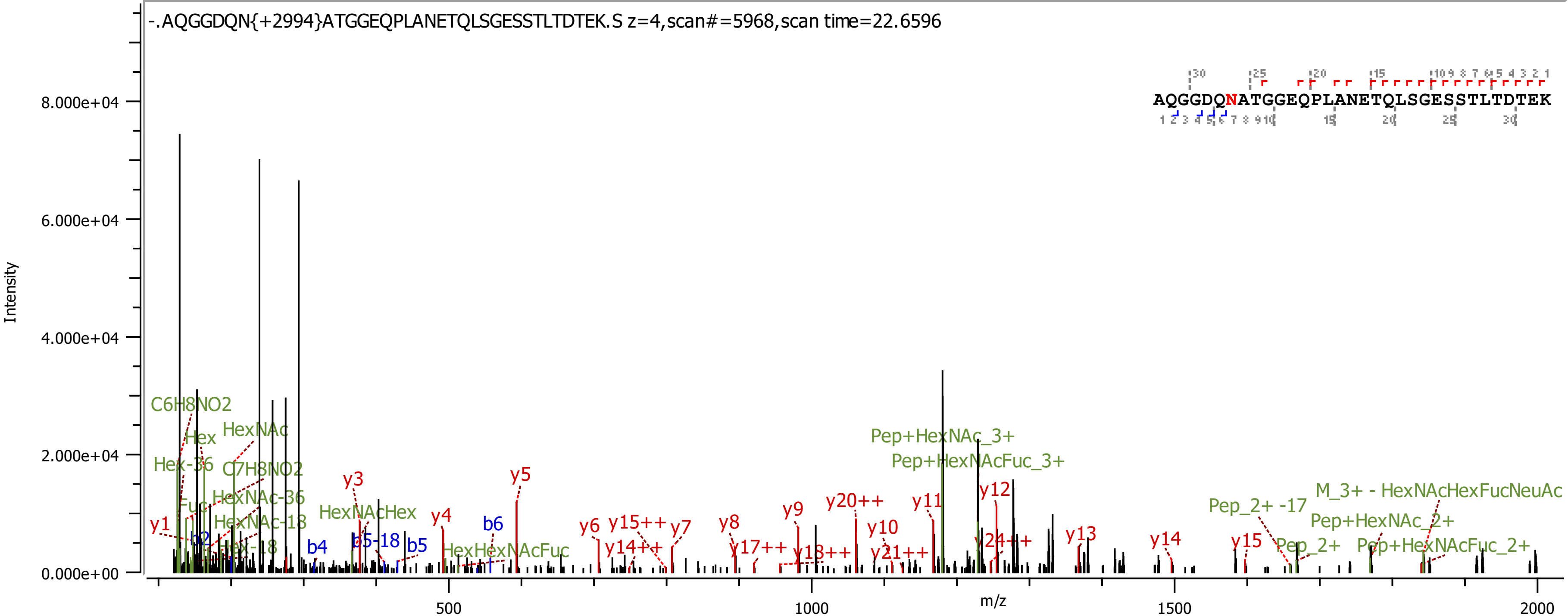

-.AQGGDQN{+2994}ATGGEQPLANETQLSGESSTLTDTEK.S z=4,scan#=5968,scan time=22.6596
AQGGDQNATGGEQPLANETQLSGESSTLTDTEK
Intensity
8.000e+04
6.000e+04
4.000e+04
2.000e+04
0.000e+00
m/z
500
1000
1500
2000
C6H8NO2
Hex
HexNAc
Hex-36
C7H8NO2
HexNAc-36
Fuc
HexNAc-18
Hex-18
y1
b2
b4
b5-18
b5
b6
y3
HexNAcHex
y4
y5
HexHexNAcFuc
y6
y15++
y14++
y7
y8
y17++
y9
y18++
y20++
y10
y21++
y11
Pep+HexNAc_3+
Pep+HexNAcFuc_3+
y12
y24++
y13
y14
y15
Pep_2+ -17
Pep_2+
M_3+ - HexNAcHexFucNeuAc
Pep+HexNAc_2+
Pep+HexNAcFuc_2+

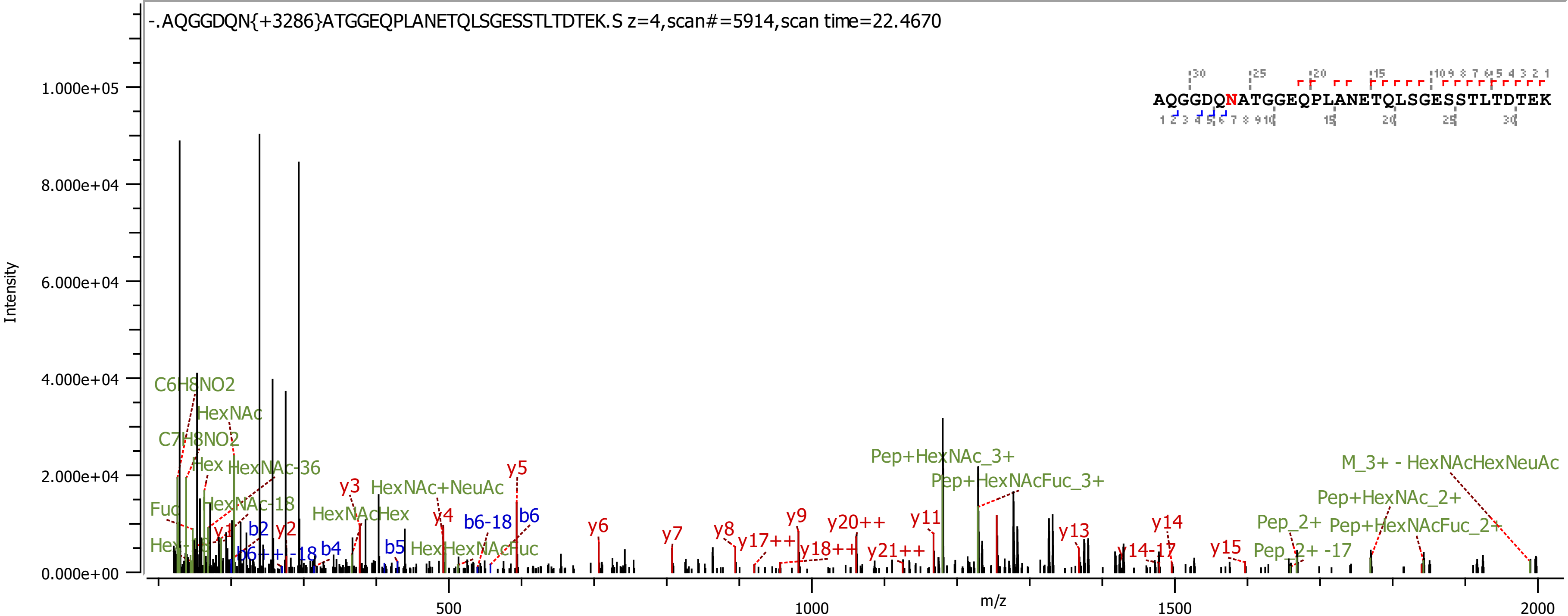
-.AQGGDQN{+3286}ATGGEQPLANETQLSGESSTLTDTEK.S z=4,scan#=5914,scan time=22.4670
AQGGDQNATGGEQPLANETQLSGESSTLTDTEK
Intensity
m/z
C6H8NO2
HexNAc
C7H8NO2
Hex
HexNAc-36
Fuc
HexNAc-18
Hex-18
HexNAc+NeuAc
HexNAcHex
HexHexNAcFuc
y1
y2
y3
y4
y5
y6
y7
y8
y9
y11
y13
y14
y14-17
y15
y17++
y18++
y20++
y21++
b2
b4
b5
b6
b6-18
b6++-18
Pep+HexNAc_3+
Pep+HexNAcFuc_3+
M_3+ - HexNAcHexNeuAc
Pep+HexNAc_2+
Pep_2+
Pep+HexNAcFuc_2+
Pep_2+ -17
0.000e+00
2.000e+04
4.000e+04
6.000e+04
8.000e+04
1.000e+05
500
1000
1500
2000

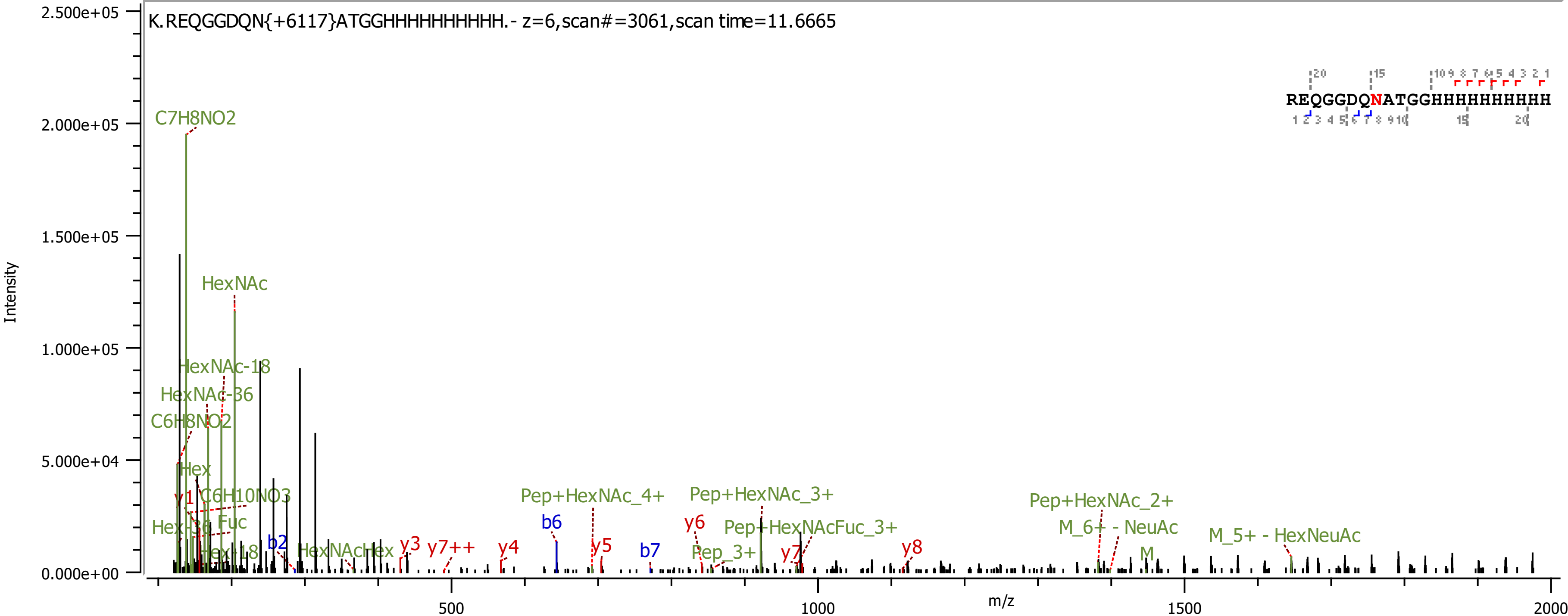
K.REQGGDQN{+6117}ATGGHHHHHHHHHH.- z=6,scan#=3061,scan time=11.6665
REQGGDQNATGGHHHHHHHHHH
Intensity
m/z
C7H8NO2
HexNAc
HexNAc-18
HexNAc-36
C6H8NO2
Hex
y1
C6H10NO3
Hex-36
Fuc
Hex-18
b2
HexNAcHex
y3
y7++
y4
b6
Pep+HexNAc_4+
y5
b7
y6
Pep+HexNAc_3+
Pep+HexNAcFuc_3+
Pep_3+
y7
y8
Pep+HexNAc_2+
M_6+ - NeuAc
M
M_5+ - HexNeuAc
0.000e+00
5.000e+04
1.000e+05
1.500e+05
2.000e+05
2.500e+05
500
1000
1500
2000

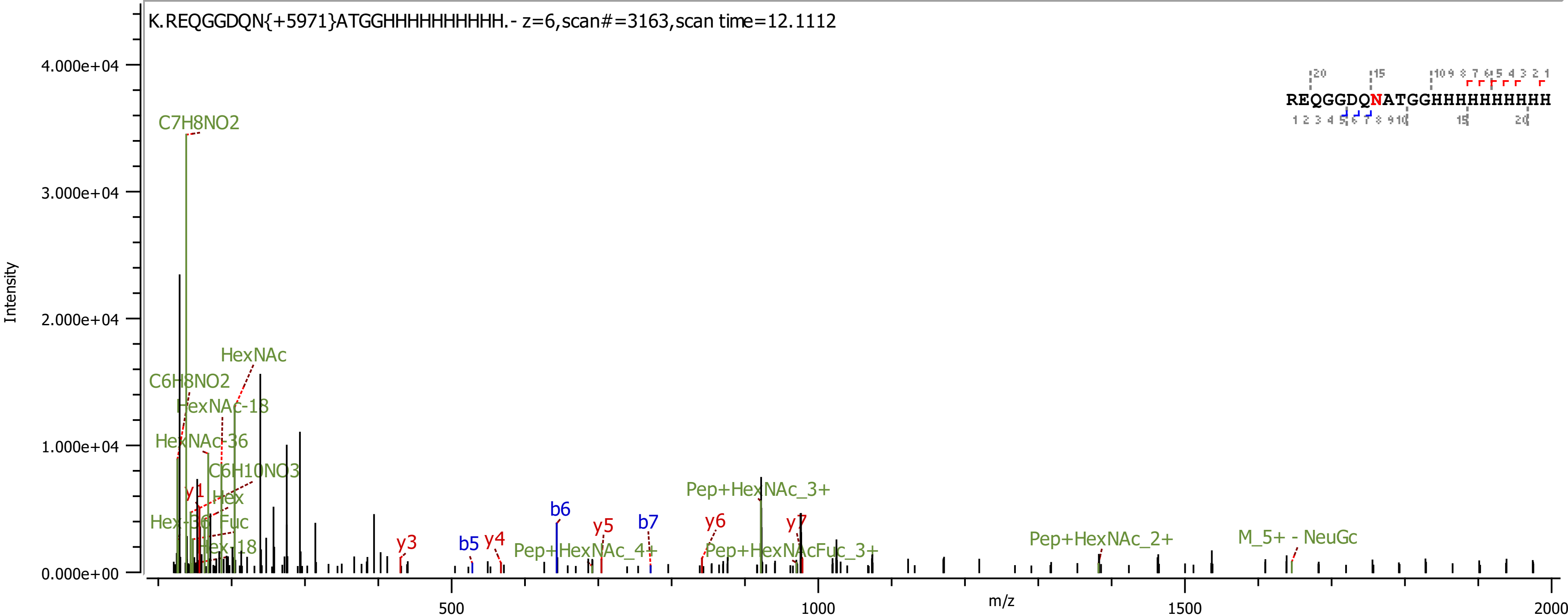
K.REQGGDQN{+5971}ATGGHHHHHHHHHH.- z=6,scan#=3163,scan time=12.1112
REQGGDQNATGGHHHHHHHHHH
Intensity
m/z
4.000e+04
3.000e+04
2.000e+04
1.000e+04
0.000e+00
500
1000
1500
2000
C7H8NO2
HexNAc
C6H8NO2
HexNAc-18
HexNAc-36
C6H10NO3
y1
Hex
Hex-36
Fuc
Hex-18
y3
b5
y4
b6
y5
b7
Pep+HexNAc_4+
y6
Pep+HexNAc_3+
y7
Pep+HexNAcFuc_3+
Pep+HexNAc_2+
M_5+ - NeuGc

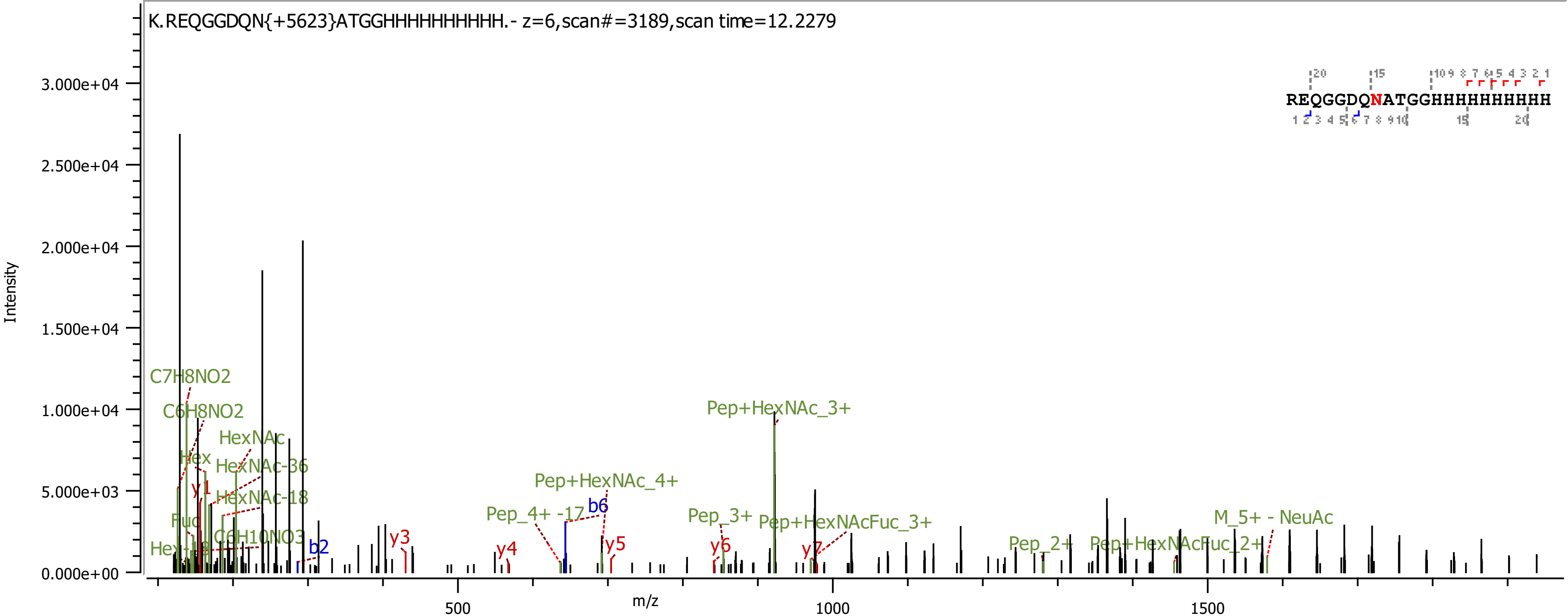

K.REQGGDQN{+5623}ATGGHHHHHHHHHH.- z=6,scan#=3189,scan time=12.2279
REQGGDQNATGGHHHHHHHHHH
Intensity
m/z
C7H8NO2
C6H8NO2
HexNAc
Hex
HexNAc-36
y1
HexNAc-18
Fuc
C6H10NO3
Hex-18
b2
y3
y4
Pep_4+ -17
b6
Pep+HexNAc_4+
y5
Pep_3+
y6
Pep+HexNAc_3+
Pep+HexNAcFuc_3+
y7
Pep_2+
Pep+HexNAcFuc_2+
M_5+ - NeuAc

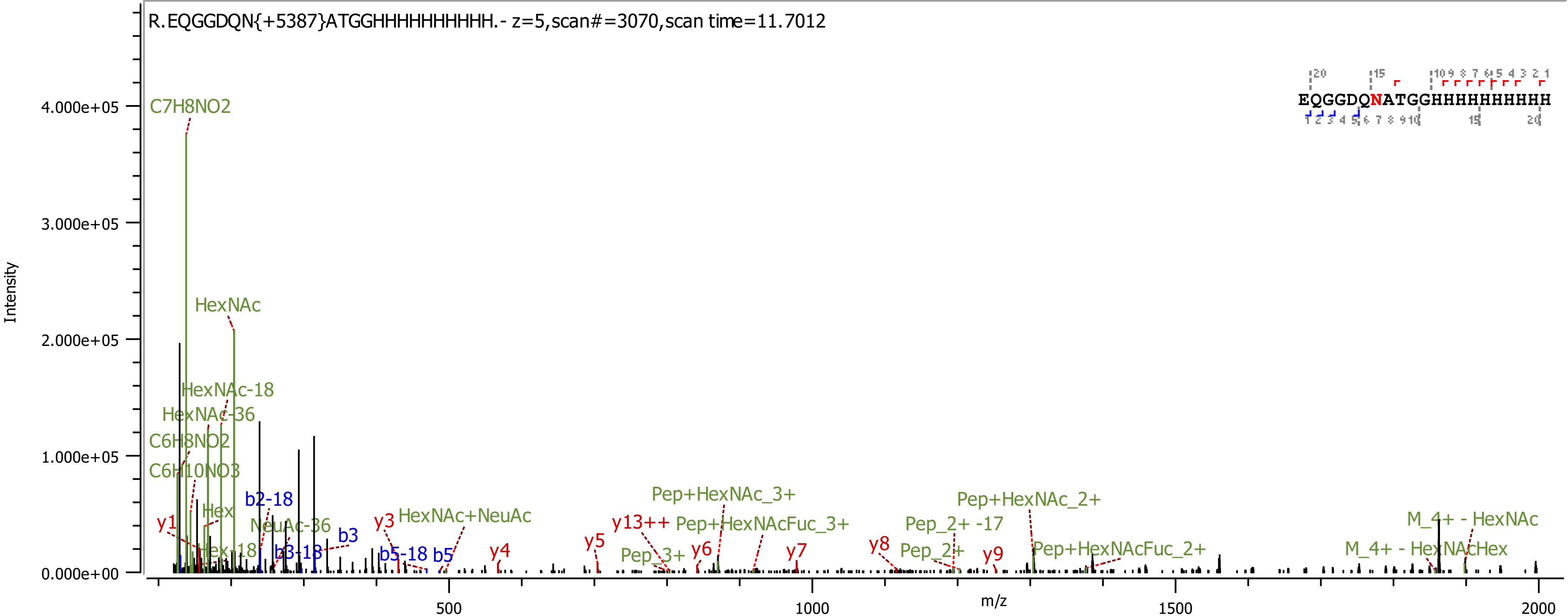
R.EQGGDQN{+5387}ATGGHHHHHHHHHH.- z=5,scan#=3070,scan time=11.7012
EQGGDQNATGGHHHHHHHHHH
Intensity
m/z
C7H8NO2
HexNAc
HexNAc-18
HexNAc-36
C6H8NO2
C6H10NO3
Hex
y1
Hex-18
b2-18
NeuAc-36
b3
b3-18
y3
HexNAc+NeuAc
b5-18
b5
y4
y5
y13++
Pep+HexNAc_3+
Pep+HexNAcFuc_3+
Pep_3+
y6
y7
y8
Pep_2+ -17
Pep_2+
y9
Pep+HexNAc_2+
Pep+HexNAcFuc_2+
M_4+ - HexNAc
M_4+ - HexNAcHex

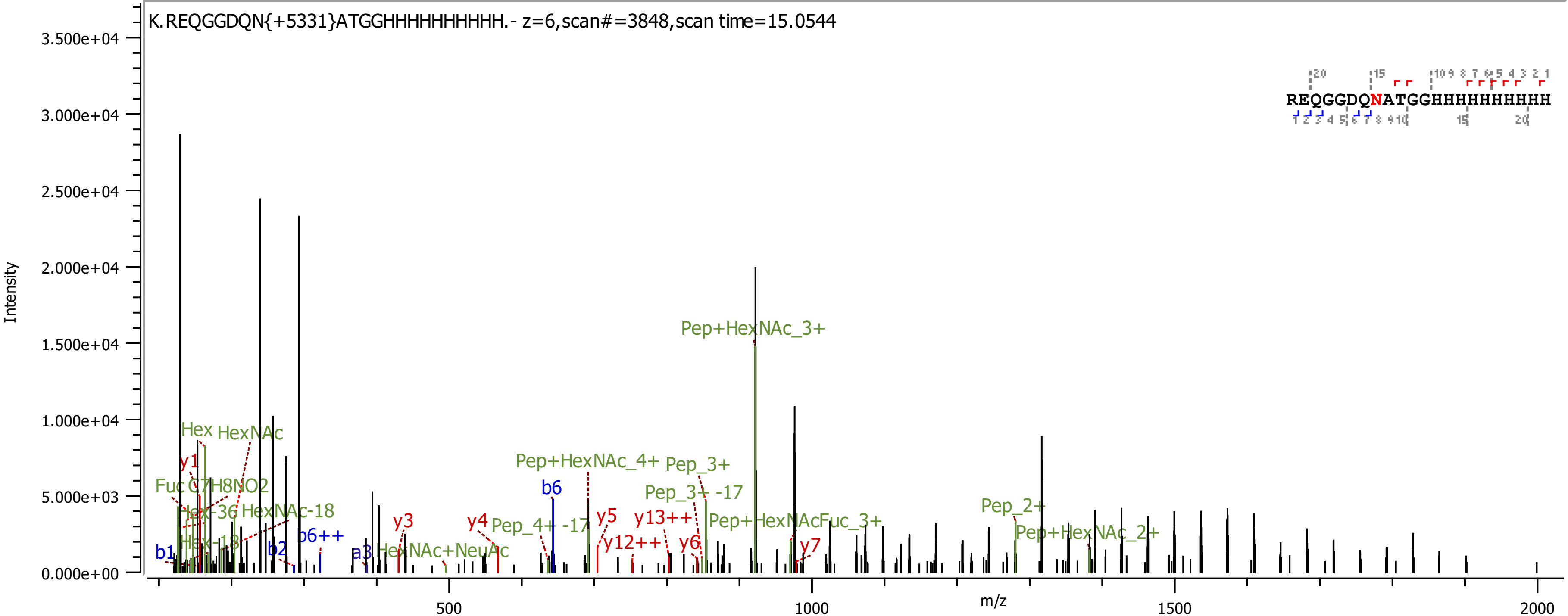

K.REQGGDQN{+5331}ATGGHHHHHHHHHH.- z=6,scan#=3848,scan time=15.0544
REQGGDQNATGGHHHHHHHHHH
Intensity
m/z
3.500e+04
3.000e+04
2.500e+04
2.000e+04
1.500e+04
1.000e+04
5.000e+03
0.000e+00
500
1000
1500
2000
Pep+HexNAc_3+
Pep+HexNAc_4+
Pep_3+
Pep_3+ -17
Pep+HexNAcFuc_3+
Pep_2+
Pep+HexNAc_2+
Pep_4+ -17
HexNAc+NeuAc
Hex
HexNAc
Fuc C7H8NO2
Hex-36
HexNAc-18
Hex-18
b1
b2
b6++
b6
a3
y1
y3
y4
y5
y6
y7
y12++
y13++

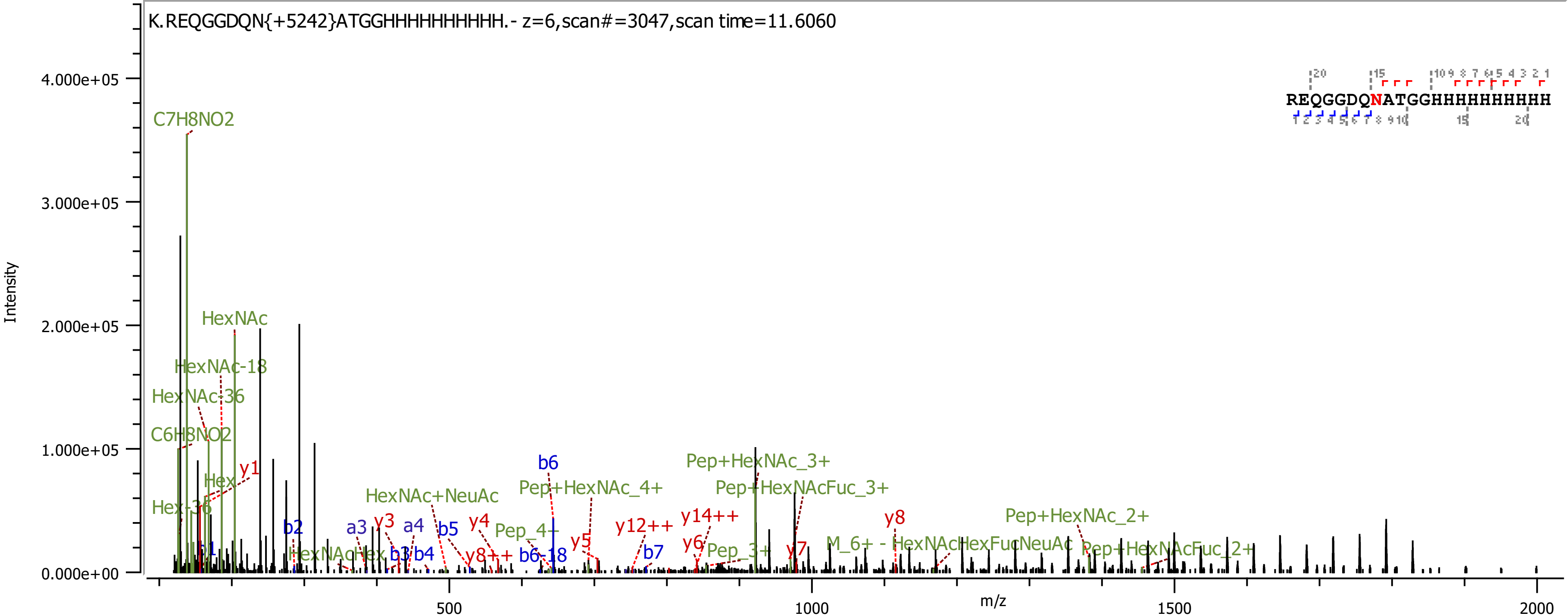
K.REQGGDQN{+5242}ATGGHHHHHHHHHH.- z=6,scan#=3047,scan time=11.6060
REQGGDQNATGGHHHHHHHHHH
Intensity
m/z
C7H8NO2
HexNAc
HexNAc-18
HexNAc-36
C6H8NO2
Hex
Hex-36
y1
b2
a3
y3
a4
b5
b3
b4
HexNAcHex
HexNAc+NeuAc
y4
y8++
b6
b6-18
Pep+HexNAc_4+
Pep_4+
y5
y12++
b7
y14++
y6
Pep_3+
Pep+HexNAc_3+
Pep+HexNAcFuc_3+
y7
y8
M_6+ - HexNAcHexFucNeuAc
Pep+HexNAc_2+
Pep+HexNAcFuc_2+

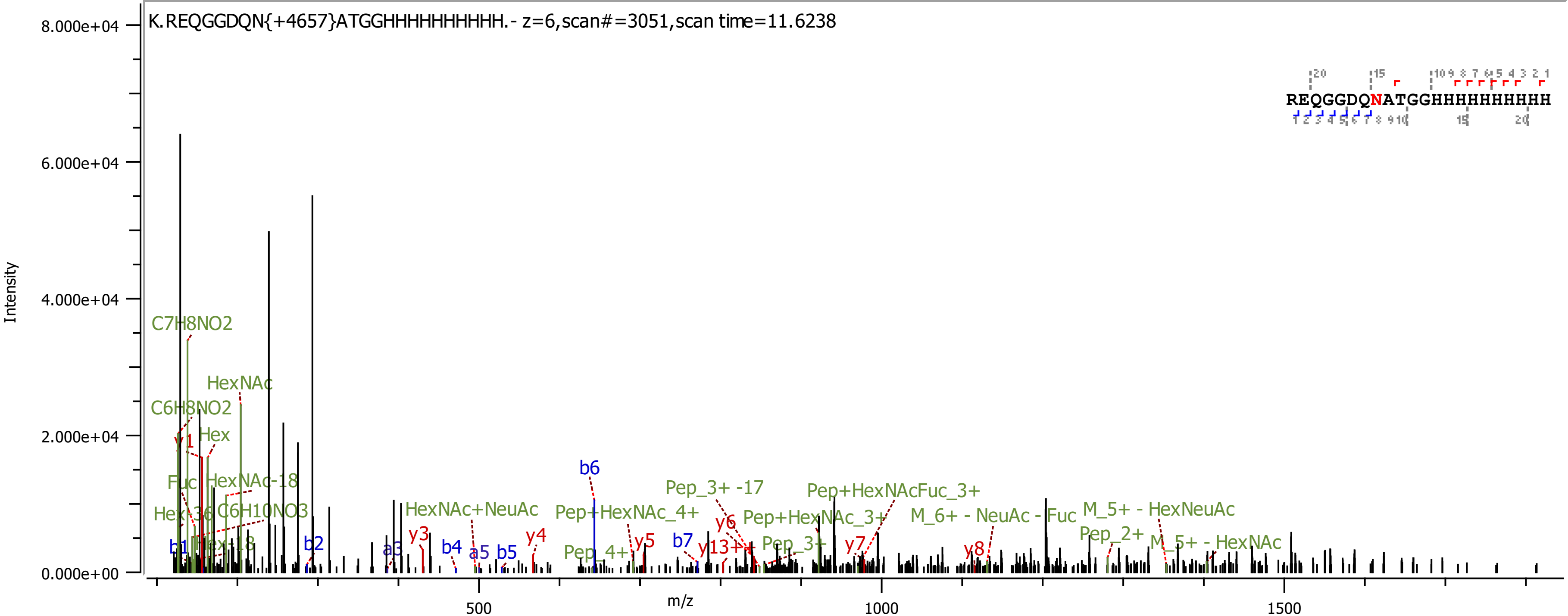

K.REQGGDQN{+4657}ATGGHHHHHHHHHH.- z=6,scan#=3051,scan time=11.6238
REQGGDQNATGGHHHHHHHHHH
Intensity
m/z
C7H8NO2
HexNAc
C6H8NO2
Hex
y1
Fuc
HexNAc-18
Hex-36
C6H10NO3
b1
Hex-18
b2
a3
y3
b4
a5
b5
y4
HexNAc+NeuAc
b6
Pep+HexNAc_4+
Pep_4+
y5
b7
Pep_3+ -17
y6
y13++
Pep+HexNAc_3+
Pep_3+
y7
Pep+HexNAcFuc_3+
M_6+ - NeuAc - Fuc
y8
M_5+ - HexNeuAc
Pep_2+
M_5+ - HexNAc

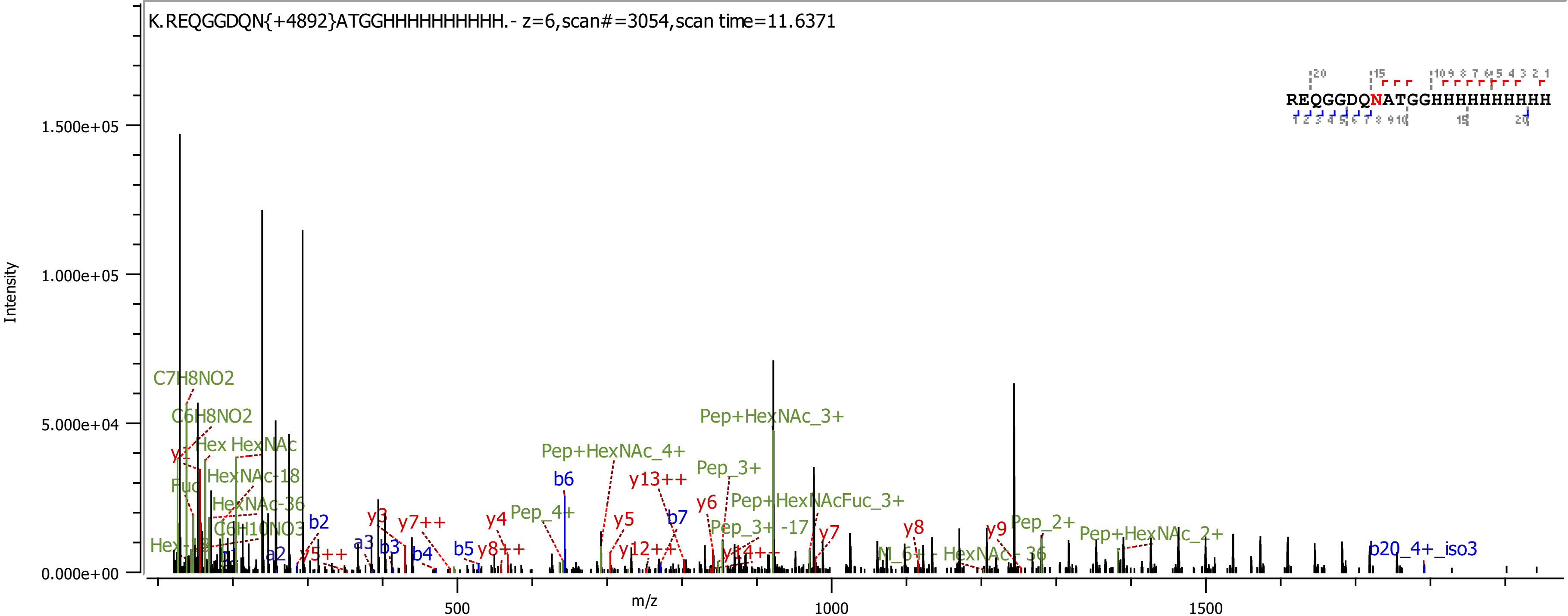
K.REQGGDQN{+4892}ATGGHHHHHHHHHH.- z=6,scan#=3054,scan time=11.6371
REQGGDQNATGGHHHHHHHHHH
Intensity
m/z
1.500e+05
1.000e+05
5.000e+04
0.000e+00
500
1000
1500
C7H8NO2
C6H8NO2
Hex HexNAc
HexNAc-18
HexNAc-36
C6H10NO3
Fuc
Hex+18
b2
b3
b4
b5
b6
b7
a2
a3
y1
y3
y4
y5
y6
y7
y8
y9
y5++
y7++
y8++
y12++
y13++
y14++
Pep_4+
Pep+HexNAc_4+
Pep_3+
Pep_3+ -17
Pep+HexNAc_3+
Pep+HexNAcFuc_3+
Pep_2+
Pep+HexNAc_2+
b20_4+_iso3

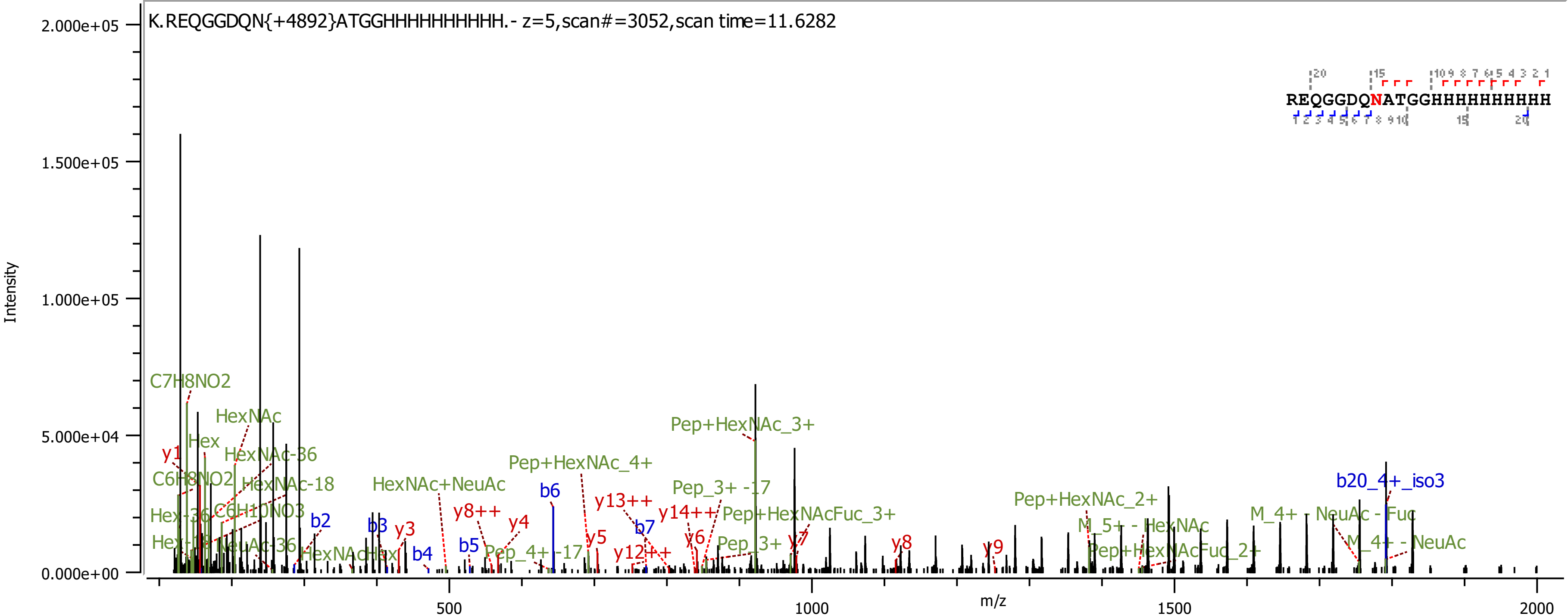
K.REQGGDQN{+4892}ATGGHHHHHHHHHH.- z=5,scan#=3052,scan time=11.6282
REQGGDQNATGGHHHHHHHHHH
Intensity
m/z
2.000e+05
1.500e+05
1.000e+05
5.000e+04
0.000e+00
500
1000
1500
2000
C7H8NO2
HexNAc
Hex
y1
HexNAc-36
C6H8NO2
HexNAc-18
Hex-36
C6H10NO3
b2
Hex+HexNAc-36
HexNAcHex
HexNAc+NeuAc
b3
y3
b4
b5
y8++
y4
Pep_4+ -17
b6
Pep+HexNAc_4+
y5
y13++
y12++
b7
y14++
y6
Pep_3+ -17
Pep+HexNAc_3+
Pep+HexNAcFuc_3+
Pep_3+
y7
y8
y9
Pep+HexNAc_2+
M_5+ - HexNAc
Pep+HexNAcFuc_2+
M_4+ - NeuAc - Fuc
M_4+ - NeuAc
b20_4+_iso3

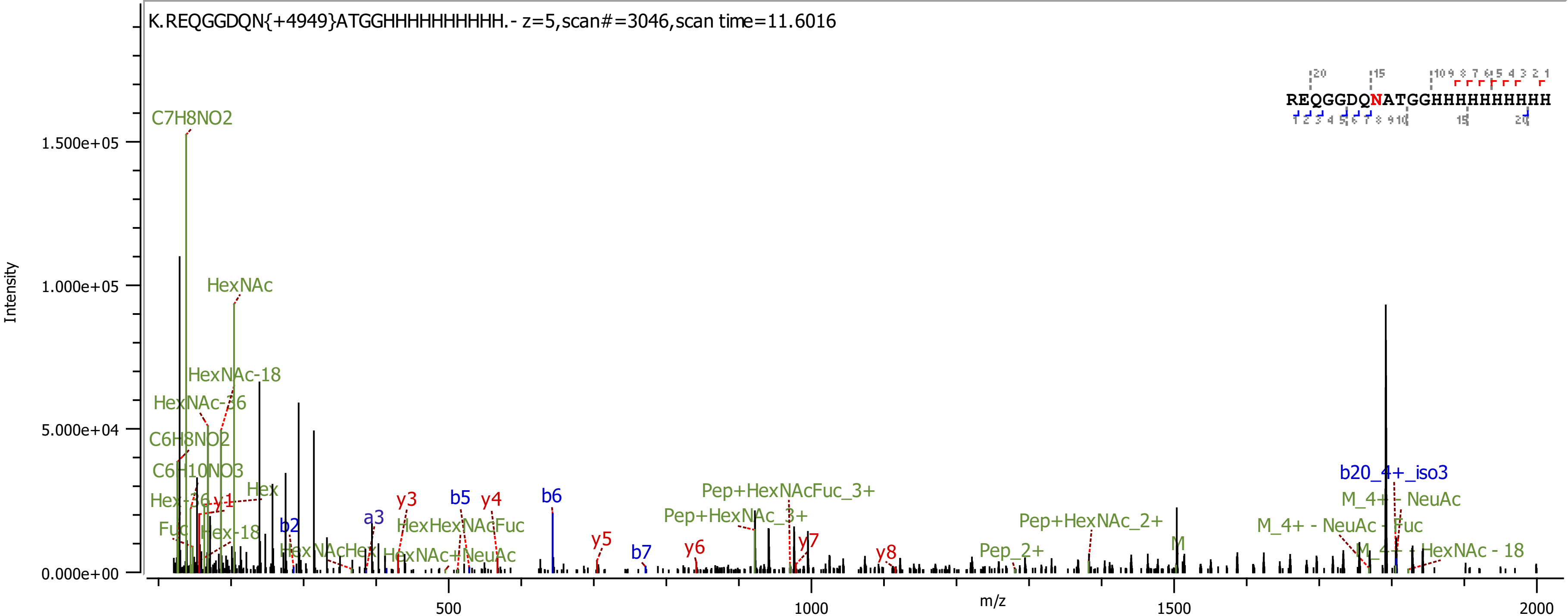

K.REQGGDQN{+4949}ATGGHHHHHHHHHH.- z=5,scan#=3046,scan time=11.6016
REQGGDQNATGGHHHHHHHHHH
Intensity
m/z
C7H8NO2
HexNAc
HexNAc-18
HexNAc-36
C6H8NO2
C6H10NO3
Hex
Hex-36
y1
Fuc
Hex-18
b2
a3
y3
b5
y4
b6
HexHexNAcFuc
HexNAcHex
HexNAc+NeuAc
y5
b7
y6
y7
y8
Pep+HexNAcFuc_3+
Pep+HexNAc_3+
Pep+HexNAc_2+
Pep_2+
M
b20_4+_iso3
M_4+ - NeuAc
M_4+ - NeuAc - Fuc
M_4+ - HexNAc - 18

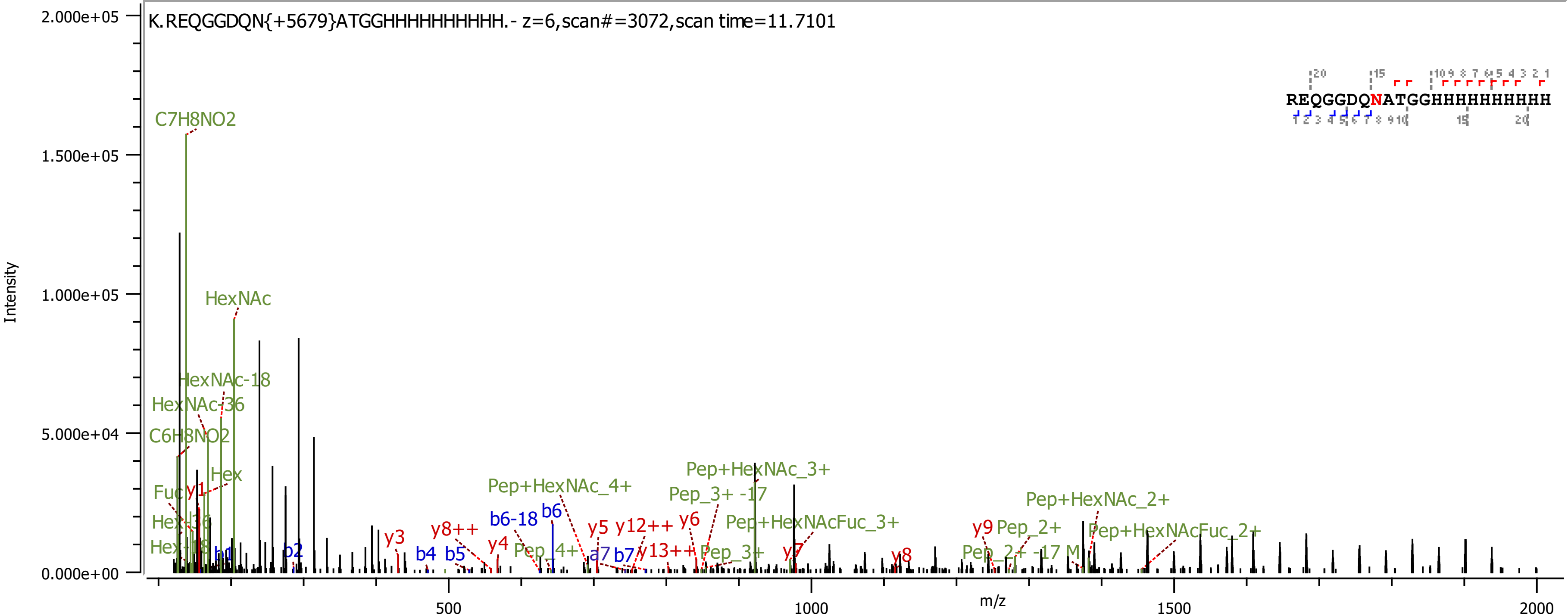
K.REQGGDQN{+5679}ATGGHHHHHHHHHH.- z=6,scan#=3072,scan time=11.7101
REQGGDQNATGGHHHHHHHHHH
Intensity
m/z
2.000e+05
1.500e+05
1.000e+05
5.000e+04
0.000e+00
500
1000
1500
2000
C7H8NO2
HexNAc
HexNAc-18
HexNAc-36
C6H8NO2
Hex
Fuc
y1
Hex-36
Hex-18
b1
b2
y3
b4
b5
y8++
y4
b6-18
b6
Pep_4+
a7
b7
Pep+HexNAc_4+
y5
y12++
y13++
y6
Pep_3+ -17
Pep+HexNAc_3+
Pep_3+
Pep+HexNAcFuc_3+
y7
y8
y9
Pep_2+
Pep_2+ -17
M
Pep+HexNAc_2+
Pep+HexNAcFuc_2+

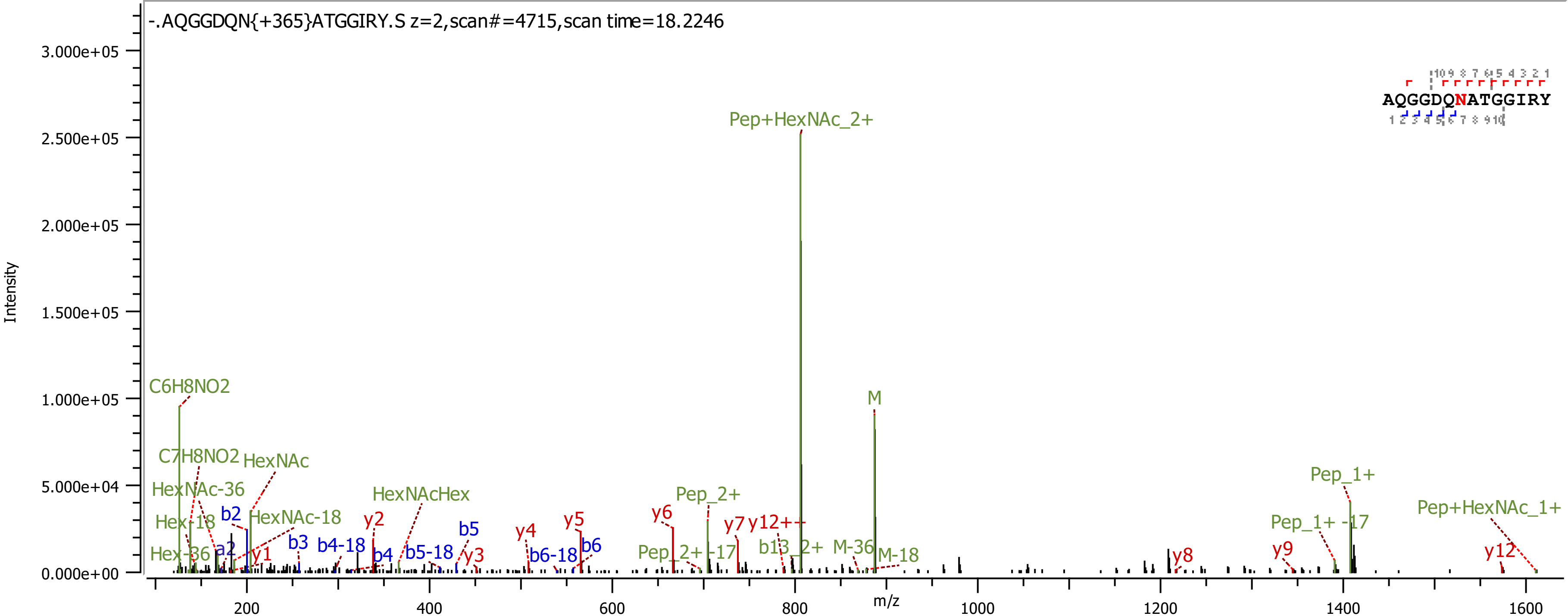
-.AQGGDQN{+365}ATGGIRY.S z=2,scan#=4715,scan time=18.2246
AQGGDQNATGGIRY
Intensity
m/z
3.000e+05
2.500e+05
2.000e+05
1.500e+05
1.000e+05
5.000e+04
0.000e+00
200
400
600
800
1000
1200
1400
1600
Pep+HexNAc_2+
C6H8NO2
C7H8NO2
HexNAc
HexNAc-36
HexNAc-18
Hex-18
Hex-36
HexNAcHex
M
M-36
M-18
Pep_2+
Pep_2+ -17
Pep_1+
Pep_1+ -17
Pep+HexNAc_1+
b13_2+
b2
b3
b4
b4-18
b5
b5-18
b6
b6-18
a2
y1
y2
y3
y4
y5
y6
y7
y8
y9
y12
y12++

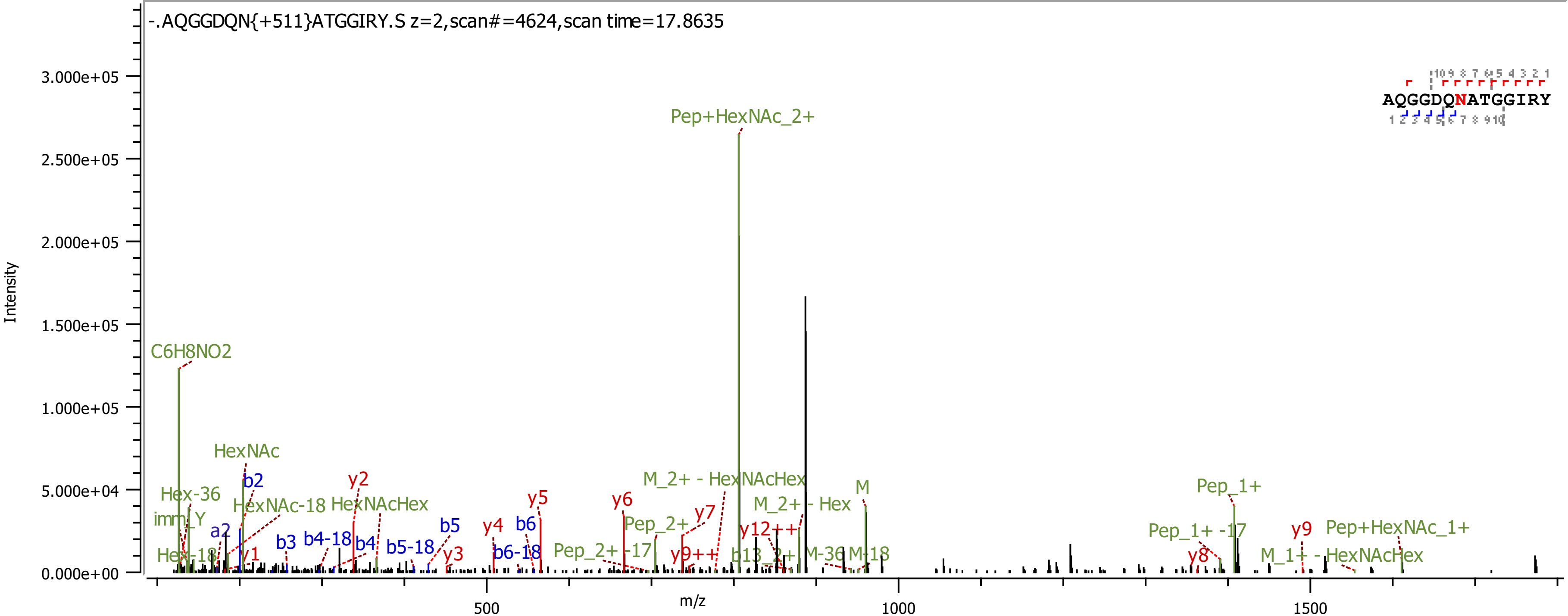

-.AQGGDQN{+511}ATGGIRY.S z=2,scan#=4624,scan time=17.8635
AQGGDQNATGGIRY
Intensity
m/z
Pep+HexNAc_2+
C6H8NO2
HexNAc
b2
Hex-36
HexNAc-18
HexNAcHex
y2
imm_Y
a2
b3
b4-18
b4
b5-18
b5
y3
y4
y5
b6
b6-18
y6
Pep_2+
Pep_2+ -17
y7
y9++
M_2+ - HexNAcHex
M_2+ - Hex
y12++
b13_2+
M-36
M-18
M
Hex-18
y1
Pep_1+
Pep_1+ -17
y8
y9
Pep+HexNAc_1+
M_1+ - HexNAcHex

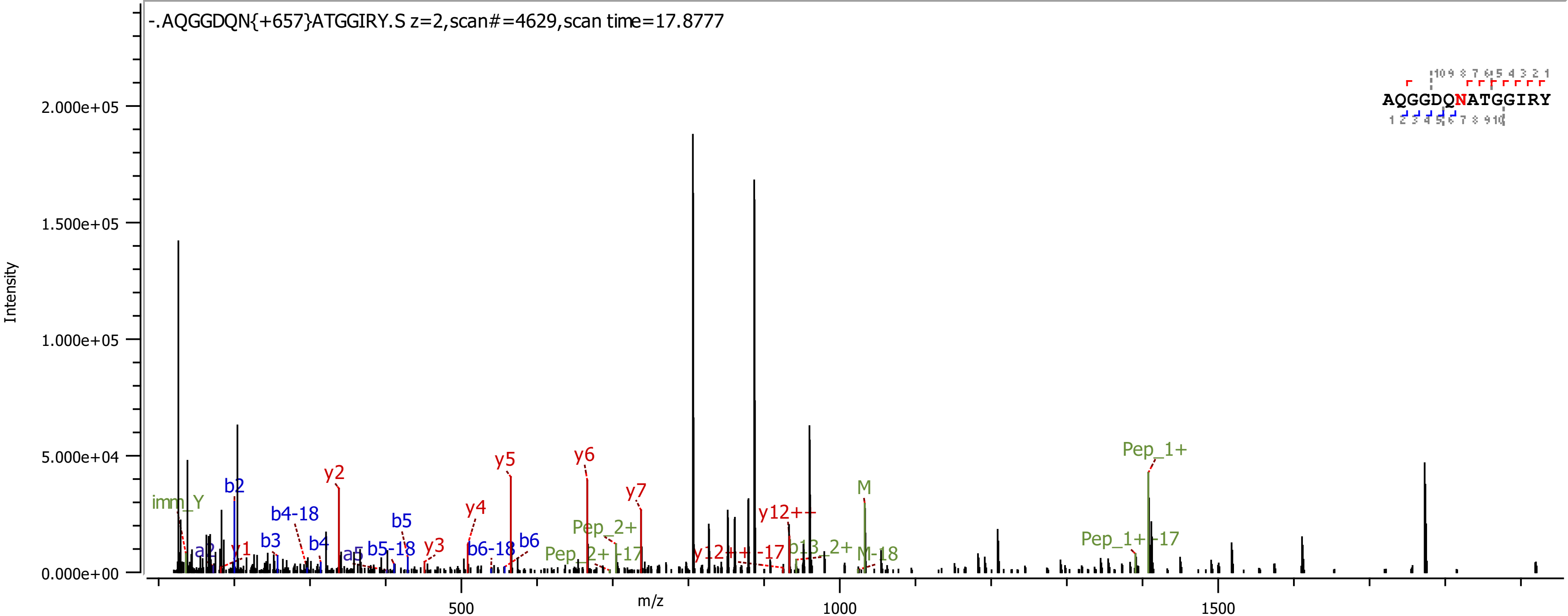

-.AQGGDQN{+657}ATGGIRY.S z=2,scan#=4629,scan time=17.8777
AQGGDQNATGGIRY
Intensity
2.000e+05
1.500e+05
1.000e+05
5.000e+04
0.000e+00
m/z
500
1000
1500
imm_Y
a2
b2
y1
b3
b4-18
b4
a5
b5-18
b5
y2
y3
y4
b6-18
y5
b6
y6
Pep_2+
Pep_2+-17
y7
y12++-17
y12++
b13_2+
M
M-18
Pep_1+
Pep_1+-17

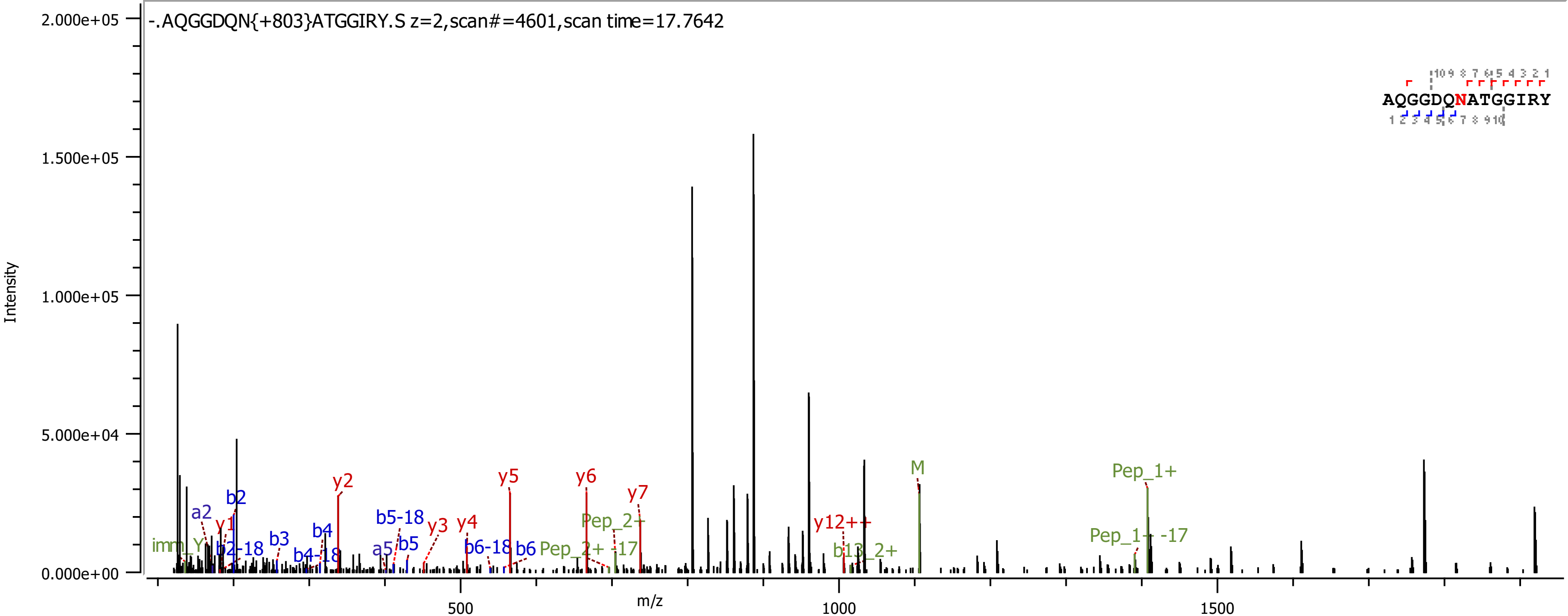
-.AQGGDQN{+803}ATGGIRY.S z=2,scan#=4601,scan time=17.7642
AQGGDQNATGGIRY
Intensity
m/z
2.000e+05
1.500e+05
1.000e+05
5.000e+04
0.000e+00
500
1000
1500
imm_Y
a2
b2
y1
b2-18
b3
b4
b4-18
y2
b5-18
a5
b5
y3
y4
y5
b6-18
b6
Pep_2+ -17
y6
Pep_2+
y7
y12++
b13_2+
M
Pep_1+
Pep_1+ -17

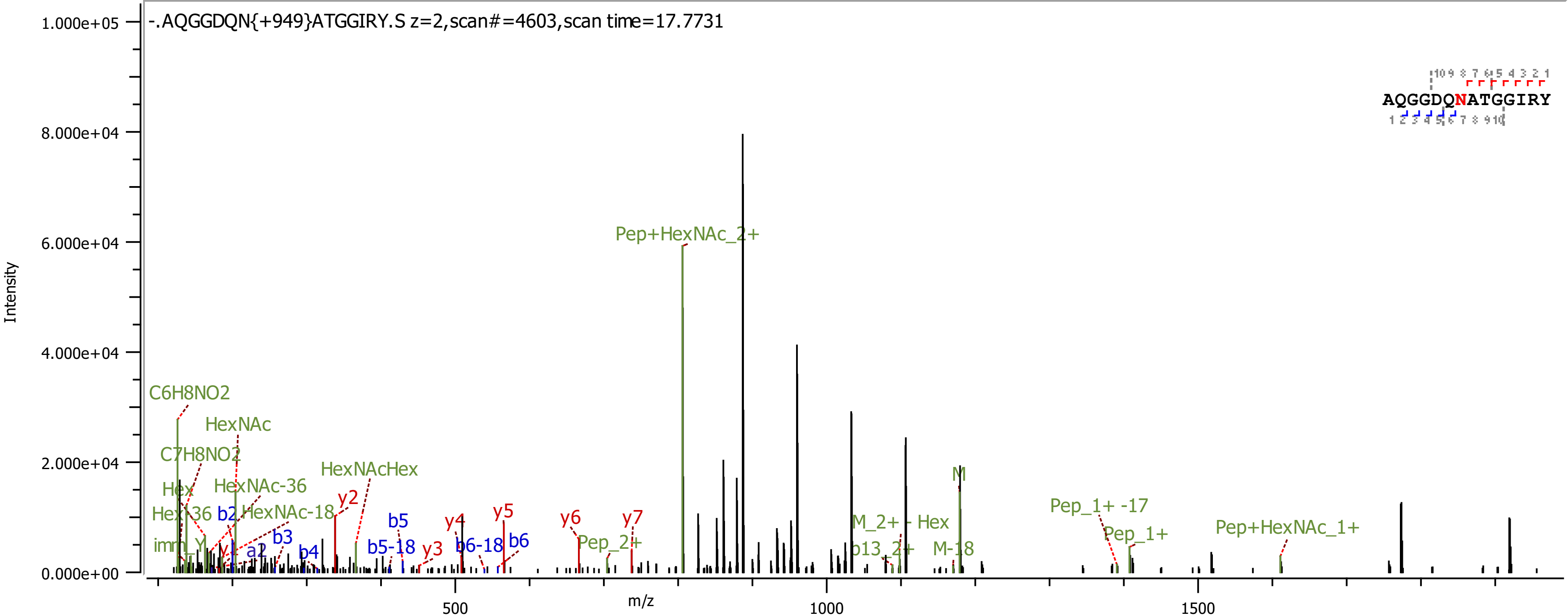

-.AQGGDQN{+949}ATGGIRY.S z=2,scan#=4603,scan time=17.7731
AQGGDQNATGGIRY
Intensity
m/z
1.000e+05
8.000e+04
6.000e+04
4.000e+04
2.000e+04
0.000e+00
500
1000
1500
Pep+HexNAc_2+
C6H8NO2
HexNAc
C7H8NO2
Hex
HexNAc-36
Hex-36
b2
HexNAc-18
HexNAcHex
y2
imm_Y
y1
a2
b3
b4
b5
b5-18
y3
y4
b6-18
y5
b6
y6
Pep_2+
y7
M_2+ - Hex
b13_2+
M
M-18
Pep_1+ -17
Pep_1+
Pep+HexNAc_1+

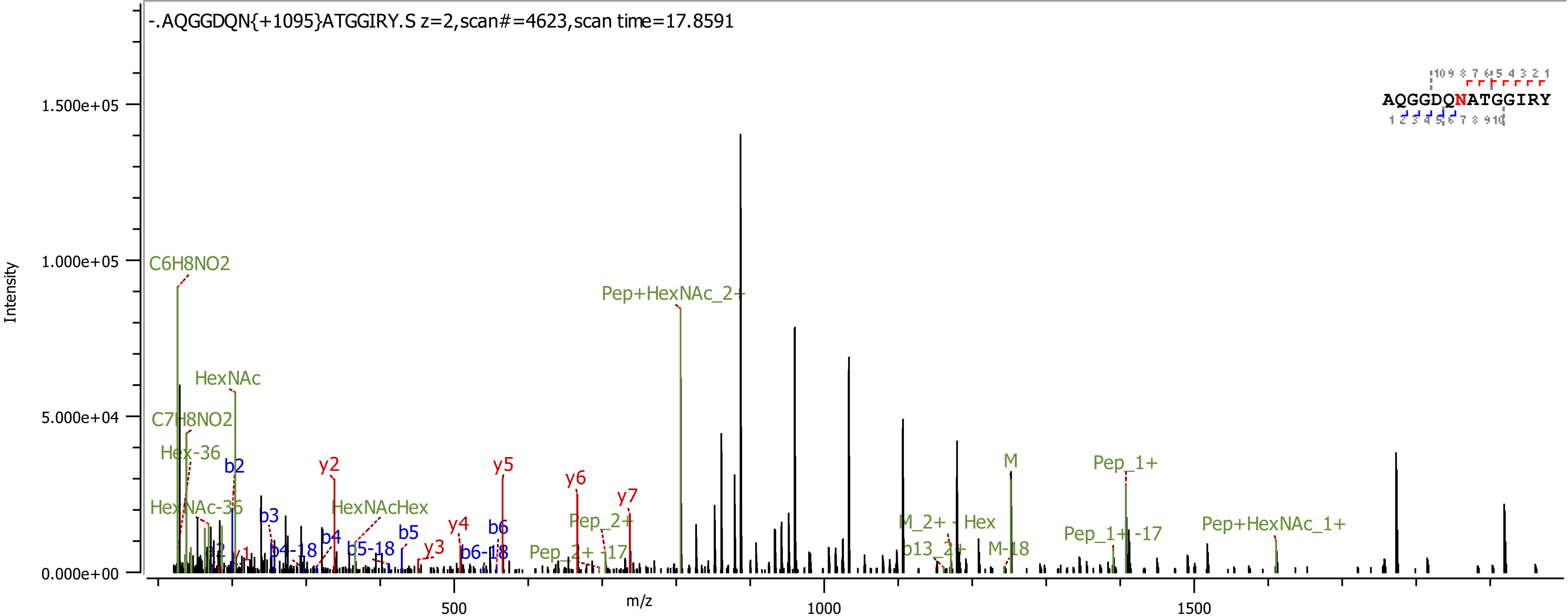

-.AQGGDQN{+1095}ATGGIRY.S z=2,scan#=4623,scan time=17.8591
AQGGDQNATGGIRY
Intensity
m/z
1.500e+05
1.000e+05
5.000e+04
0.000e+00
500
1000
1500
C6H8NO2
HexNAc
C7H8NO2
Hex-36
HexNAc-36
b2
b3
b4
b4-18
b5
b5-18
b6
b6-18
y1
y2
y3
y4
y5
y6
y7
HexNAcHex
Pep_2+
Pep_2+ -17
Pep+HexNAc_2+
M_2+ - Hex
b13_2+
M
M-18
Pep_1+
Pep_1+ -17
Pep+HexNAc_1+

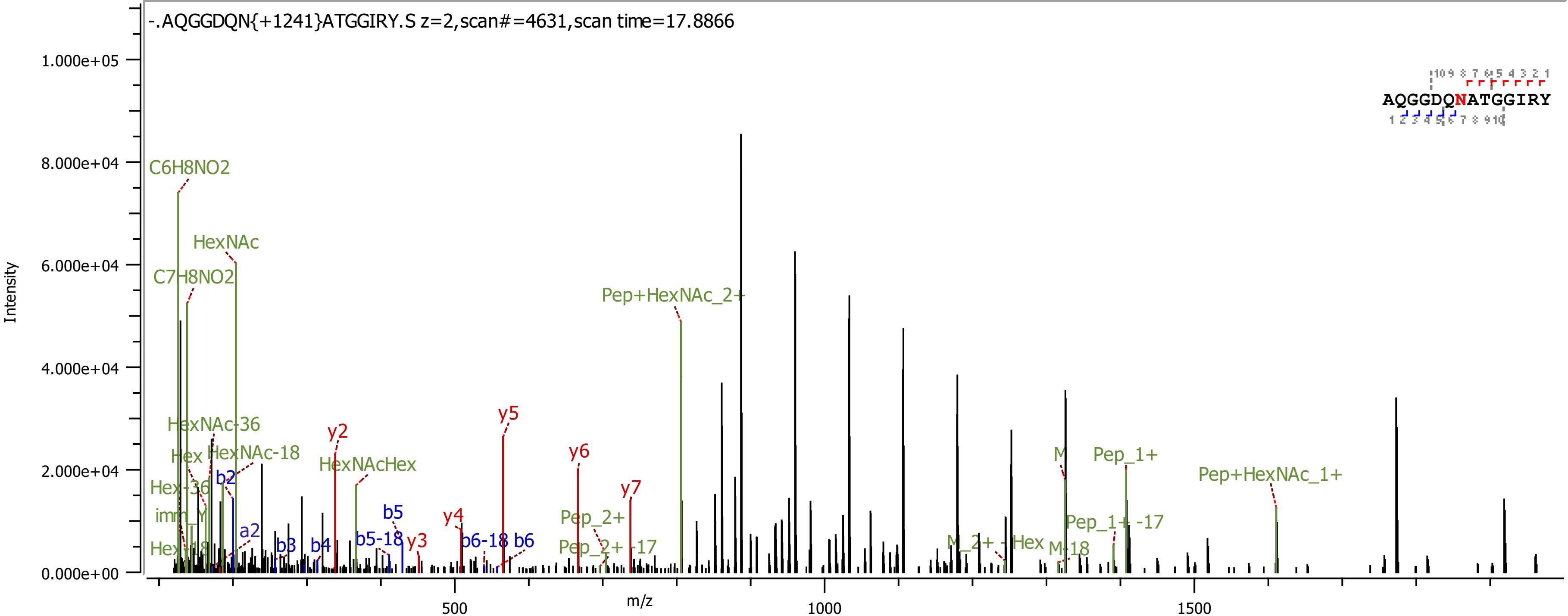

-.AQGGDQN{+1241}ATGGIRY.S z=2,scan#=4631,scan time=17.8866
Intensity
m/z
AQGGDQNATGGIRY
C6H8NO2
HexNAc
C7H8NO2
Pep+HexNAc_2+
HexNAc-36
Hex
HexNAc-18
Hex-36
imm_Y
Hex-18
b2
a2
b3
b4
y2
HexNAcHex
b5
b5-18
y3
y4
y5
b6-18
b6
y6
Pep_2+
Pep_2+ -17
y7
M_2+ - Hex
M
Pep_1+
Pep_1+ -17
M-18
Pep+HexNAc_1+

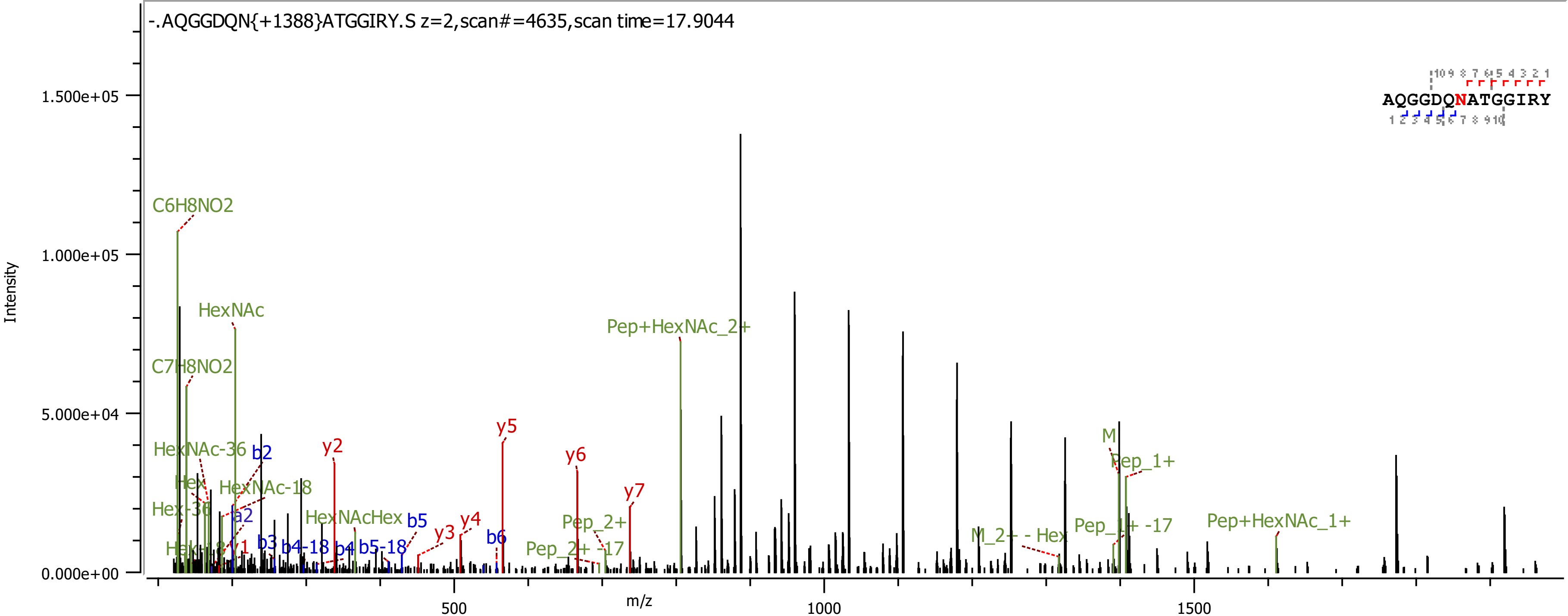

-.AQGGDQN{+1388}ATGGIRY.S z=2,scan#=4635,scan time=17.9044
AQGGDQNATGGIRY
Intensity
m/z
1.500e+05
1.000e+05
5.000e+04
0.000e+00
500
1000
1500
C6H8NO2
HexNAc
C7H8NO2
Pep+HexNAc_2+
HexNAc-36
b2
y2
y5
y6
y7
Hex
HexNAc-18
Hex-36
a2
HexNAcHex
b5
y4
y3
b6
b3
b4-18
b4
b5-18
y1
Pep_2+
Pep_2+ -17
M
Pep_1+
Pep_1+ -17
M_2+ - Hex
Pep+HexNAc_1+

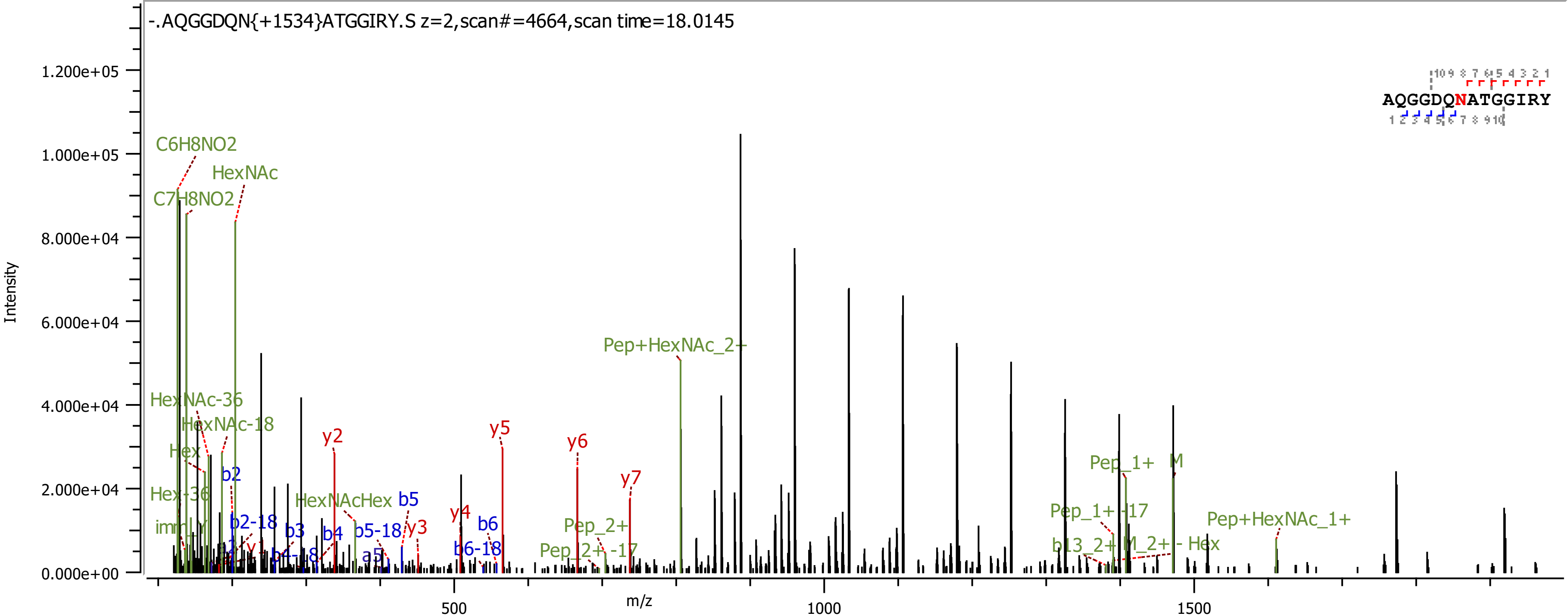
-.AQGGDQN{+1534}ATGGIRY.S z=2,scan#=4664,scan time=18.0145
AQGGDQNATGGIRY
Intensity
m/z
C6H8NO2
C7H8NO2
HexNAc
HexNAc-36
HexNAc-18
Hex
Hex-36
b2
b2-18
b3
b4
b5
b5-18
a5
y2
y3
y4
y5
y6
y7
b6
b6-18
HexNAcHex
Pep+HexNAc_2+
Pep_2+
Pep_2+-17
Pep_1+
Pep_1+-17
M
b13_2+
M_2+-Hex
Pep+HexNAc_1+
0.000e+00
2.000e+04
4.000e+04
6.000e+04
8.000e+04
1.000e+05
1.200e+05
500
1000
1500

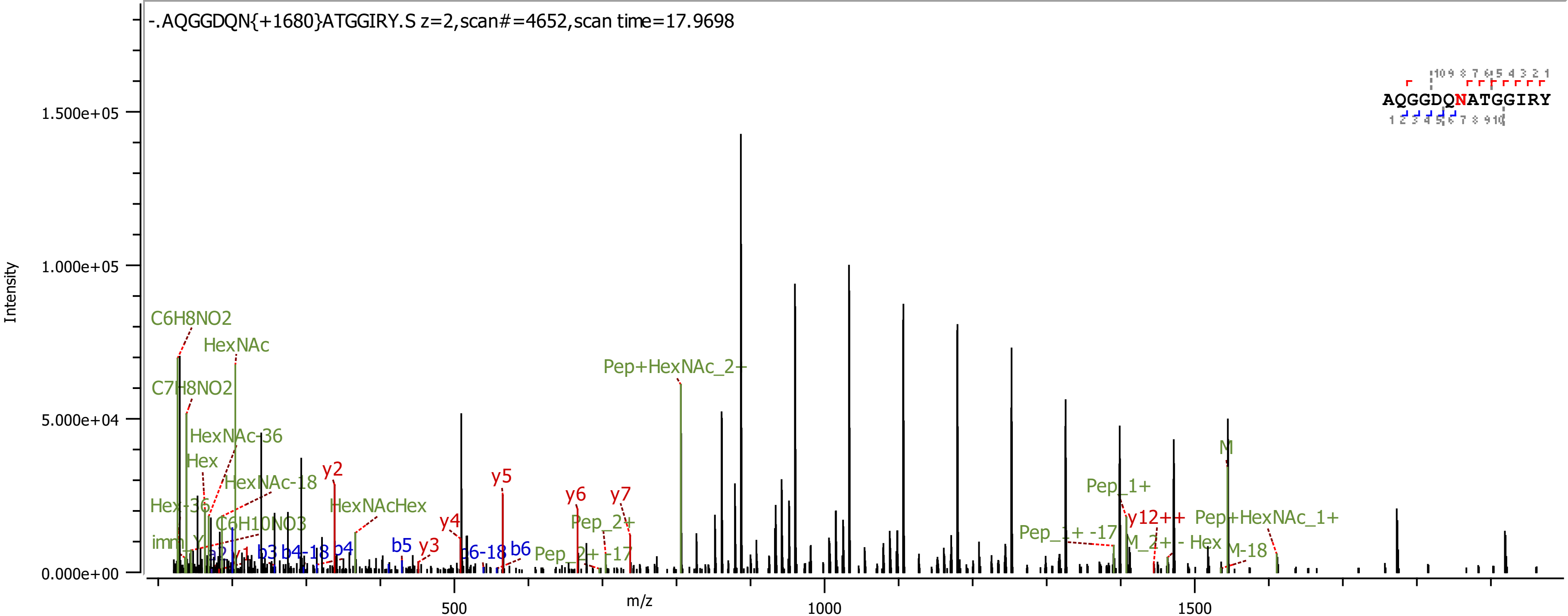
-.AQGGDQN{+1680}ATGGIRY.S z=2,scan#=4652,scan time=17.9698
AQGGDQNATGGIRY
Intensity
m/z
1.500e+05
1.000e+05
5.000e+04
0.000e+00
500
1000
1500
C6H8NO2
HexNAc
C7H8NO2
HexNAc-36
Hex
HexNAc-18
Hex-36
C6H10NO3
imm_Y
b3
b4-18
b4
b5
y2
y3
y4
y5
y6
y7
b6-18
b6
HexNAcHex
Pep+HexNAc_2+
Pep_2+
Pep_2+ -17
Pep_1+
Pep_1+ -17
y12++
Pep+HexNAc_1+
M
M-18

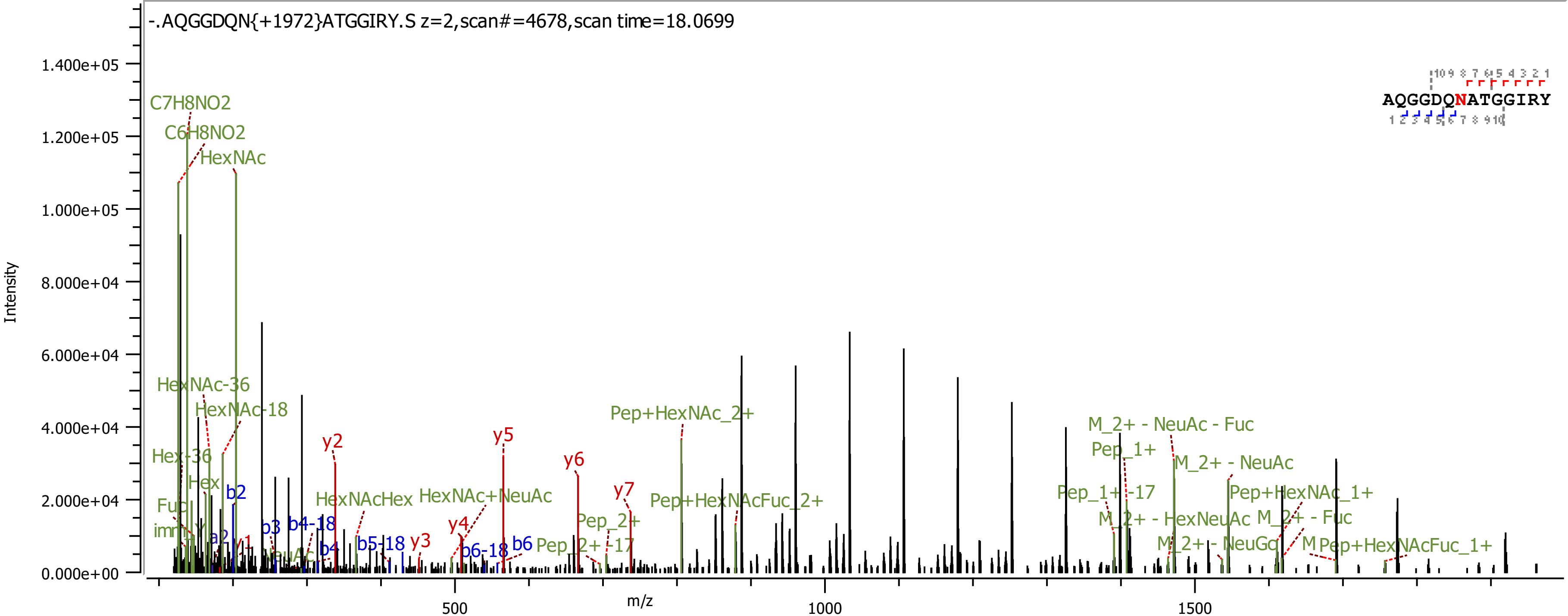
-.AQGGDQN{+1972}ATGGIRY.S z=2,scan#=4678,scan time=18.0699
AQGGDQNATGGIRY
Intensity
m/z
C7H8NO2
C6H8NO2
HexNAc
HexNAc-36
HexNAc-18
Hex-36
Hex
Fuc
b2
b3
b4-18
b4
b5-18
b6-18
b6
y2
y3
y4
y5
y6
y7
HexNAcHex
HexNAc+NeuAc
NeuAc
Pep+HexNAc_2+
Pep+HexNAcFuc_2+
Pep_2+
Pep_2+ -17
M_2+ - NeuAc - Fuc
Pep_1+
M_2+ - NeuAc
Pep_1+ -17
Pep+HexNAc_1+
M_2+ - HexNeuAc
M_2+ - Fuc
M_2+ - NeuGc
Pep+HexNAcFuc_1+
M

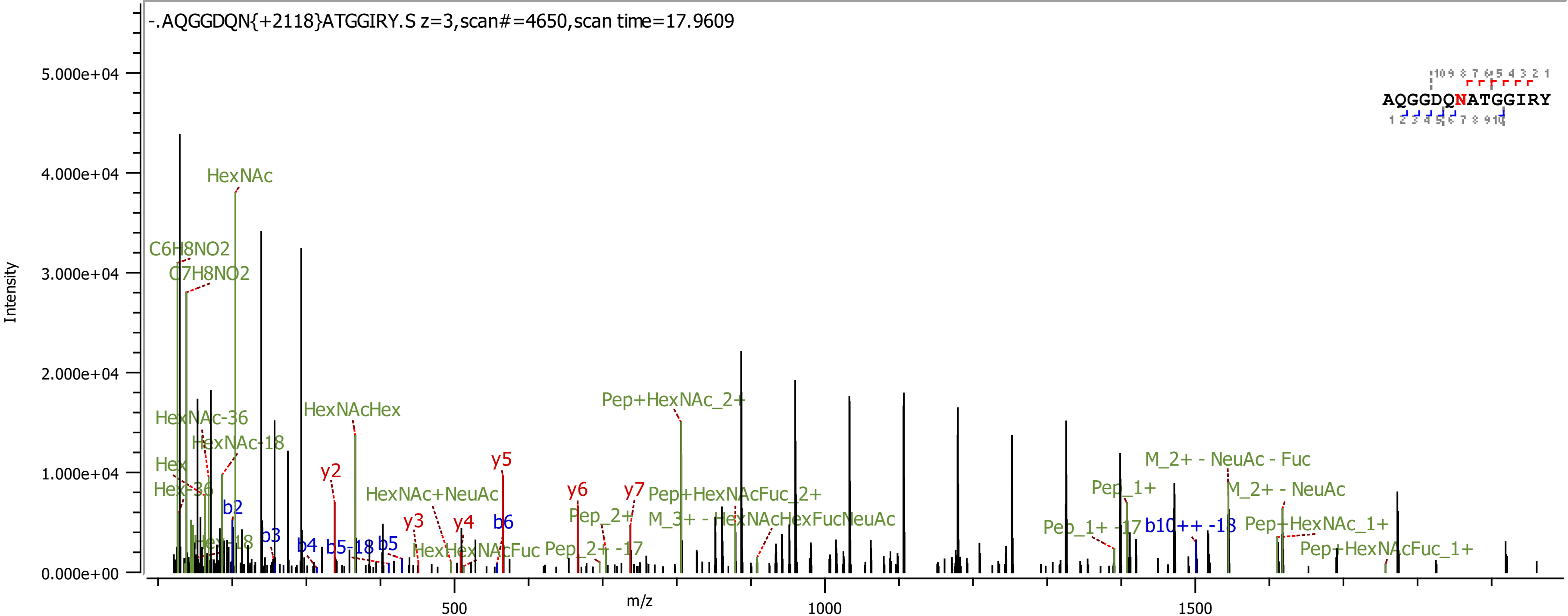

-.AQGGDQN{+2118}ATGGIRY.S z=3,scan#=4650,scan time=17.9609
Intensity
m/z
AQGGDQNATGGIRY
HexNAc
C6H8NO2
C7H8NO2
HexNAcHex
HexNAc-36
HexNAc-18
Hex
Hex-36
Hex-18
b2
b3
b4
b5-18
b5
b6
y2
y3
y4
y5
y6
y7
HexNAc+NeuAc
HexHexNAcFuc
Pep_2+
Pep_2+ -17
Pep+HexNAc_2+
Pep+HexNAcFuc_2+
M_3+ - HexNAcHexFucNeuAc
Pep_1+
Pep_1+ -17
b10++ -18
M_2+ - NeuAc - Fuc
M_2+ - NeuAc
Pep+HexNAc_1+
Pep+HexNAcFuc_1+

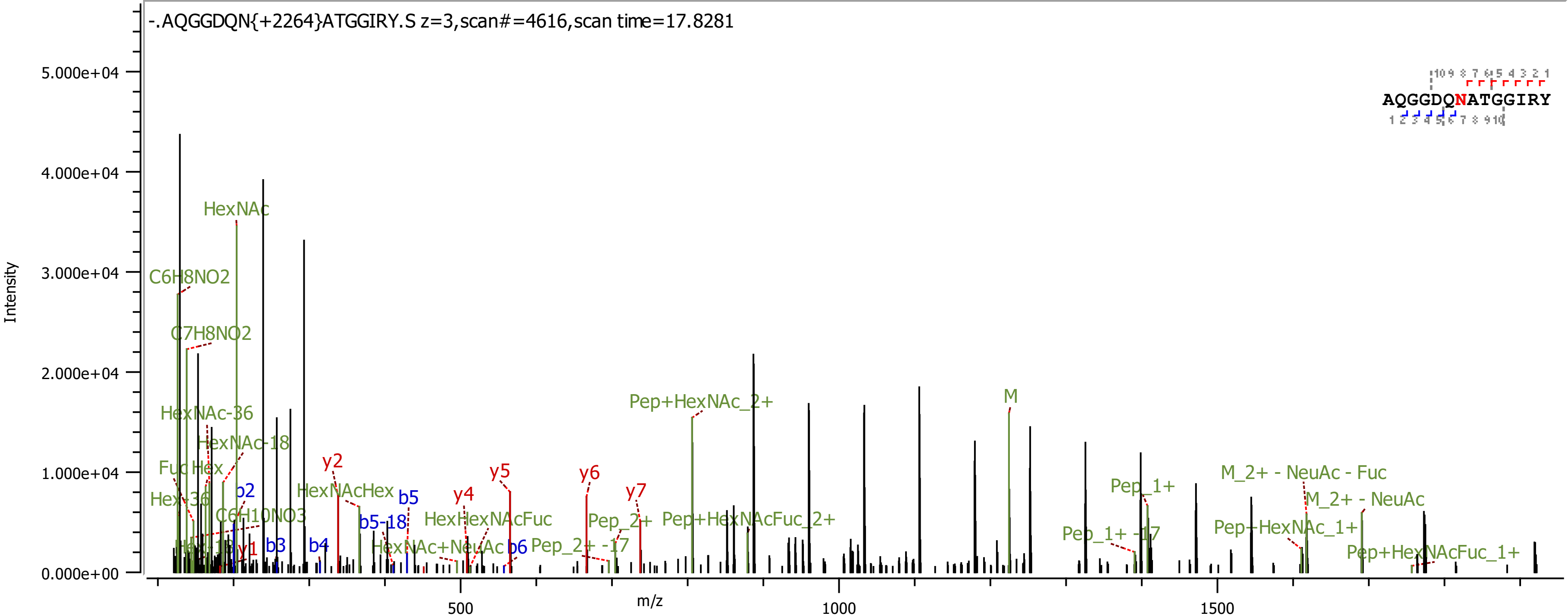

-.AQGGDQN{+2264}ATGGIRY.S z=3,scan#=4616,scan time=17.8281
AQGGDQNATGGIRY
Intensity
m/z
5.000e+04
4.000e+04
3.000e+04
2.000e+04
1.000e+04
0.000e+00
500
1000
1500
HexNAc
C6H8NO2
C7H8NO2
HexNAc-36
HexNAc-18
Fuc Hex
Hex-36
b2
C6H10NO3
y1
b3
b4
y2
HexNAcHex
b5
b5-18
y4
y5
HexHexNAcFuc
HexNAc+NeuAc
b6
y6
y7
Pep_2+
Pep_2+ -17
Pep+HexNAc_2+
Pep+HexNAcFuc_2+
M
Pep_1+
Pep_1+ -17
M_2+ - NeuAc - Fuc
M_2+ - NeuAc
Pep+HexNAc_1+
Pep+HexNAcFuc_1+

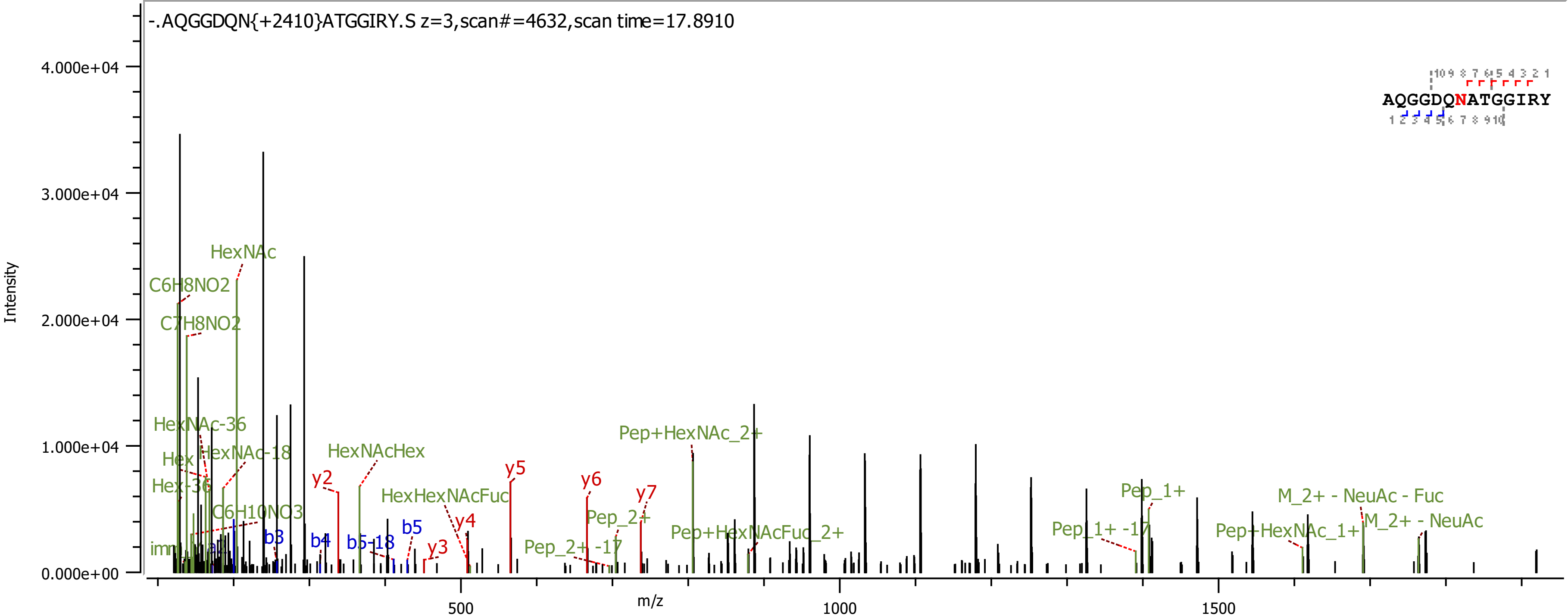
-.AQGGDQN{+2410}ATGGIRY.S z=3,scan#=4632,scan time=17.8910
Intensity
m/z
AQGGDQNATGGIRY
HexNAc
C6H8NO2
C7H8NO2
HexNAc-36
Hex
HexNAc-18
Hex-36
C6H10NO3
HexNAcHex
HexHexNAcFuc
y2
y3
y4
y5
y6
y7
b3
b4
b5-18
b5
Pep_2+
Pep_2+ -17
Pep+HexNAc_2+
Pep+HexNAcFuc_2+
Pep_1+
Pep_1+ -17
Pep+HexNAc_1+
M_2+ - NeuAc - Fuc
M_2+ - NeuAc
4.000e+04
3.000e+04
2.000e+04
1.000e+04
0.000e+00
500
1000
1500

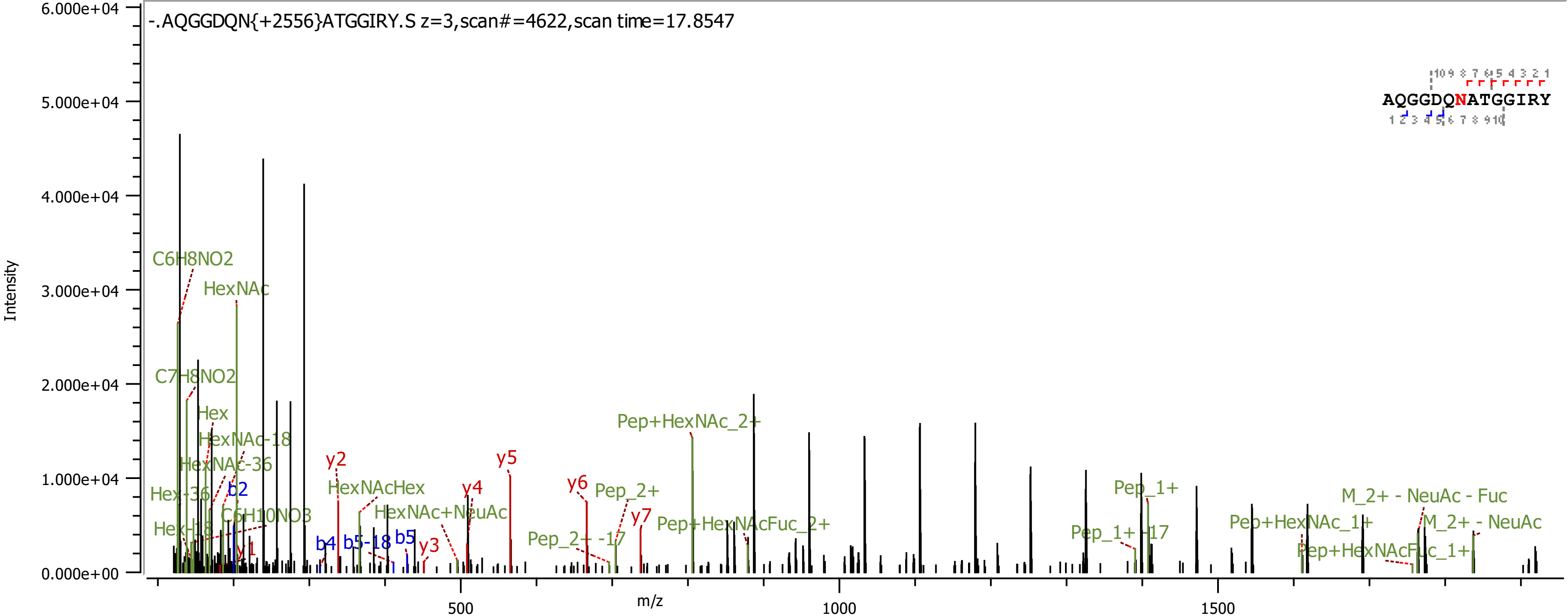

-.AQGGDQN{+2556}ATGGIRY.S z=3,scan#=4622,scan time=17.8547
AQGGDQNATGGIRY
Intensity
m/z
6.000e+04
5.000e+04
4.000e+04
3.000e+04
2.000e+04
1.000e+04
0.000e+00
500
1000
1500
C6H8NO2
HexNAc
C7H8NO2
Hex
HexNAc-18
HexNAc-36
Hex-36
b2
Hex-18
C6H10NO3
y1
b4
b5-18
b5
y3
y2
HexNAcHex
HexNAc+NeuAc
y4
y5
y6
Pep_2+
Pep_2+ -17
y7
Pep+HexNAc_2+
Pep+HexNAcFuc_2+
Pep_1+
Pep_1+ -17
Pep+HexNAc_1+
M_2+ - NeuAc - Fuc
M_2+ - NeuAc
Pep+HexNAcFuc_1+

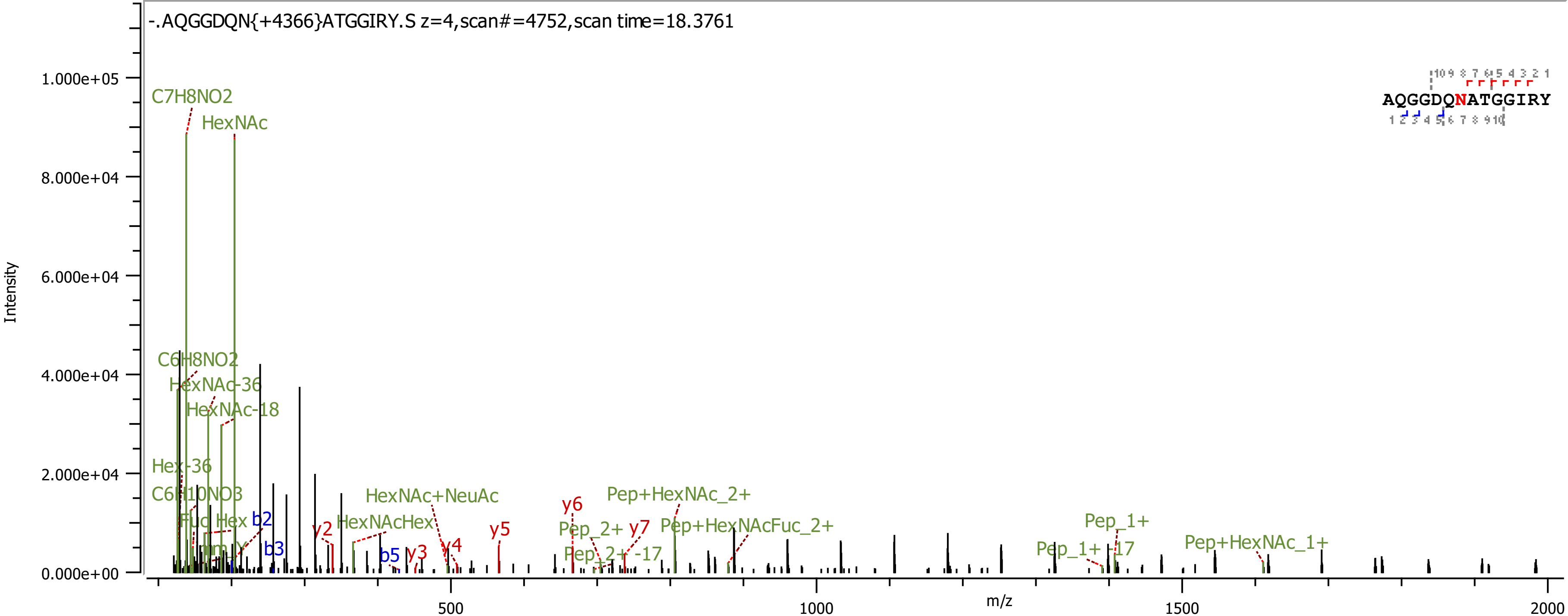
-.AQGGDQN{+4366}ATGGIRY.S z=4,scan#=4752,scan time=18.3761
Intensity
m/z
AQGGDQNATGGIRY
C7H8NO2
HexNAc
C6H8NO2
HexNAc-36
HexNAc-18
Hex-36
C6H10NO3
Fuc
Hex
b2
b3
b5
y2
y3
y4
y5
y6
y7
HexNAc+NeuAc
HexNAcHex
Pep+HexNAc_2+
Pep_2+
Pep_2+ -17
Pep+HexNAcFuc_2+
Pep_1+
Pep_1+ -17
Pep+HexNAc_1+

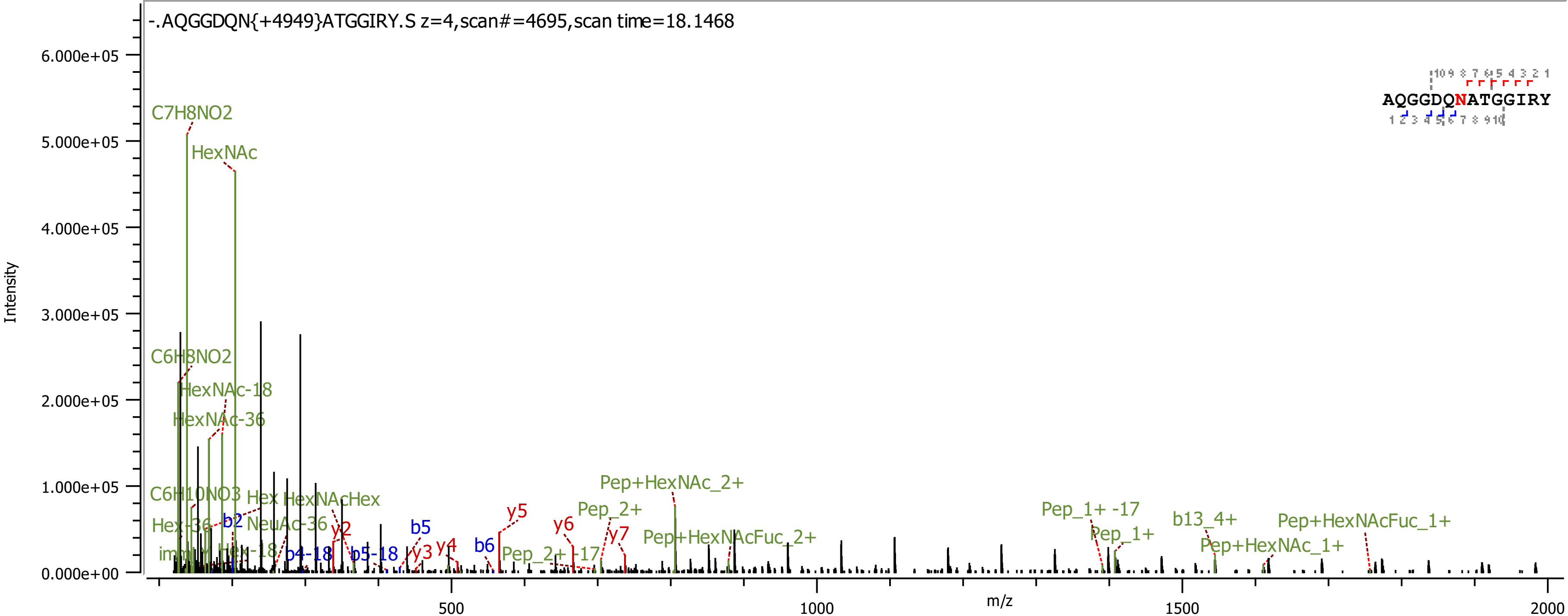
-.AQGGDQN{+4949}ATGGIRY.S z=4,scan#=4695,scan time=18.1468
Intensity
m/z
6.000e+05
5.000e+05
4.000e+05
3.000e+05
2.000e+05
1.000e+05
0.000e+00
500
1000
1500
2000
AQGGDQNATGGIRY
C7H8NO2
HexNAc
C6H8NO2
HexNAc-18
HexNAc-36
C6H10NO3
Hex
HexNAcHex
Hex-36
b2
NeuAc-36
y2
b5
y5
y6
Pep_2+
Pep+HexNAc_2+
y7
Pep+HexNAcFuc_2+
b4-18
b5-18
y3
y4
b6
Pep_2+ -17
Hex-18
Pep_1+ -17
Pep_1+
b13_4+
Pep+HexNAc_1+
Pep+HexNAcFuc_1+

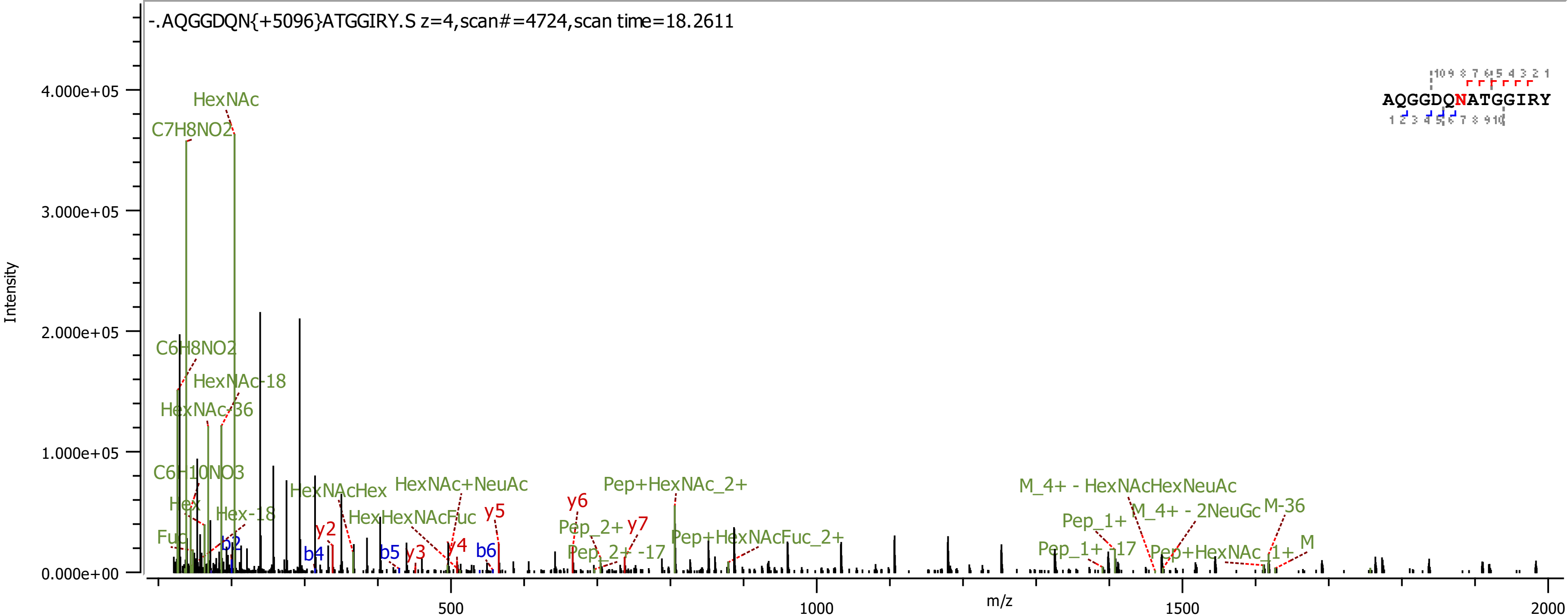
-.AQGGDQN{+5096}ATGGIRY.S z=4,scan#=4724,scan time=18.2611
Intensity
m/z
AQGGDQNATGGIRY
HexNAc
C7H8NO2
C6H8NO2
HexNAc-18
HexNAc-36
C6H10NO3
Hex
Hex-18
Fuc
HexNAcHex
HexNAc+NeuAc
HexHexNAcFuc
Pep+HexNAc_2+
Pep+HexNAcFuc_2+
Pep_2+
Pep_2+ -17
M_4+ - HexNAcHexNeuAc
M_4+ - 2NeuGc
M-36
Pep_1+
Pep_1+ -17
Pep+HexNAc_1+
M
y2
y3
y4
y5
y6
y7
b2
b4
b5
b6

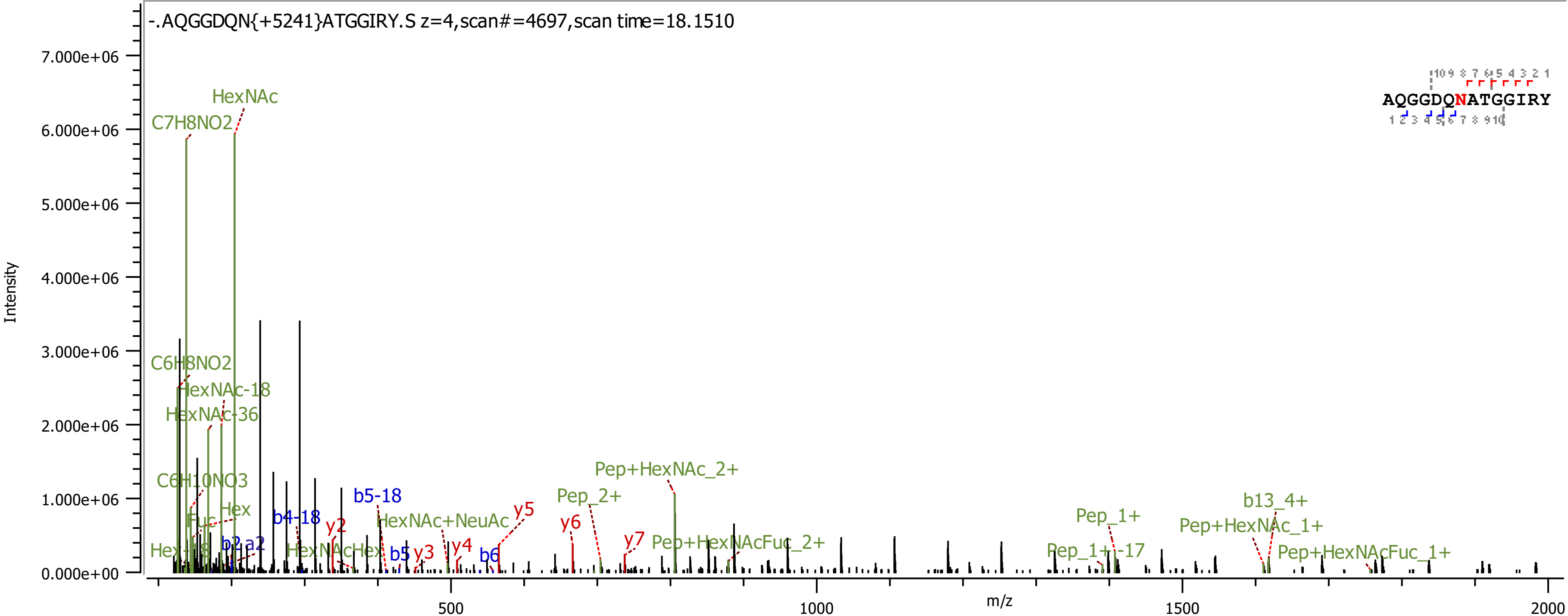
-.AQGGDQN{+5241}ATGGIRY.S z=4,scan#=4697,scan time=18.1510
Intensity
m/z
7.000e+06
6.000e+06
5.000e+06
4.000e+06
3.000e+06
2.000e+06
1.000e+06
0.000e+00
500
1000
1500
2000
AQGGDQNATGGIRY
HexNAc
C7H8NO2
C6H8NO2
HexNAc-18
HexNAc-36
C6H10NO3
Hex
Fuc
Hex-18
b2
a2
b4-18
b5-18
y2
HexNAc+NeuAc
HexNAcHex
b5
y3
y4
b6
y5
y6
Pep_2+
Pep+HexNAc_2+
y7
Pep+HexNAcFuc_2+
Pep_1+
Pep_1+-17
b13_4+
Pep+HexNAc_1+
Pep+HexNAcFuc_1+

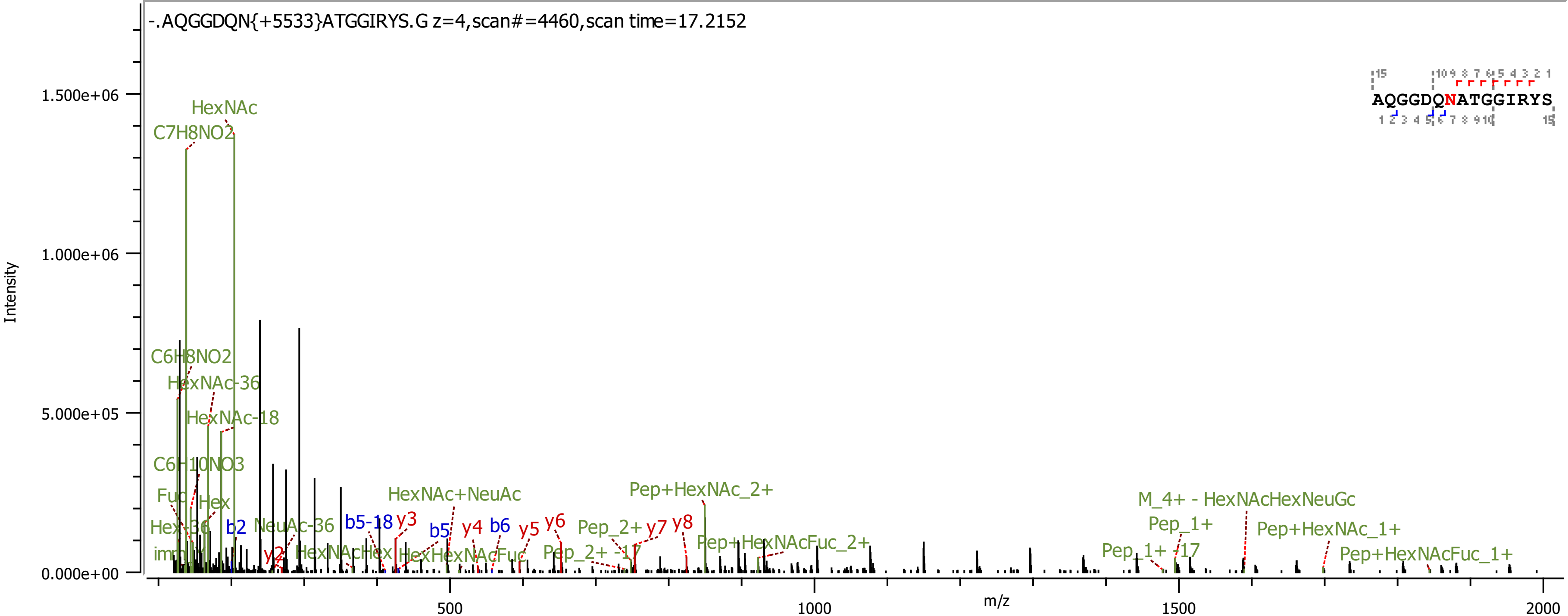

-.AQGGDQN{+5533}ATGGIRYS.G z=4,scan#=4460,scan time=17.2152
AQGGDQNATGGIRYS
Intensity
m/z
1.500e+06
1.000e+06
5.000e+05
0.000e+00
500
1000
1500
2000
HexNAc
C7H8NO2
C6H8NO2
HexNAc-36
HexNAc-18
C6H10NO3
Fuc
Hex
Hex-36
imm(Y)
b2
NeuAc-36
y2
HexNAcHex
b5-18
y3
b5
HexNAc+NeuAc
y4
b6
HexHexNAcFuc
y5
y6
Pep_2+ -17
Pep_2+
y7
y8
Pep+HexNAc_2+
Pep+HexNAcFuc_2+
M_4+ - HexNAcHexNeuGc
Pep_1+
Pep_1+ -17
Pep+HexNAc_1+
Pep+HexNAcFuc_1+

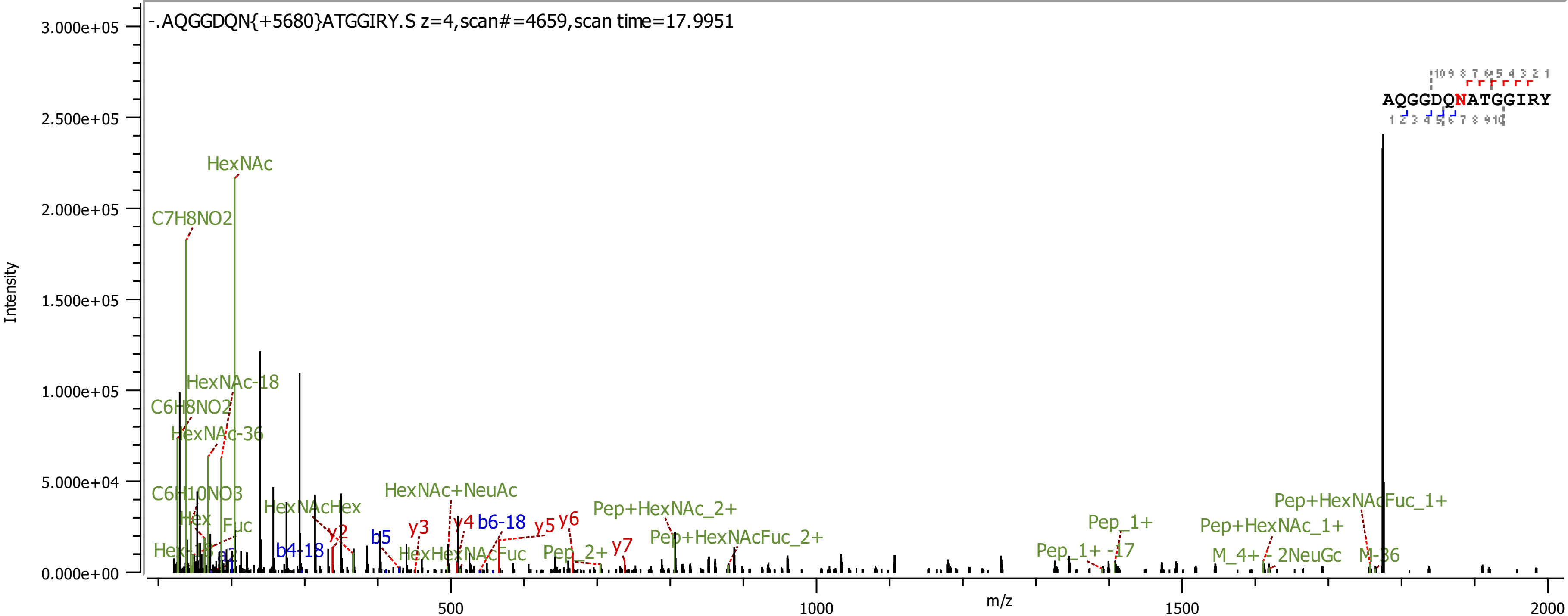

-.AQGGDQN{+5680}ATGGIRY.S z=4,scan#=4659,scan time=17.9951
AQGGDQNATGGIRY
Intensity
m/z
HexNAc
C7H8NO2
HexNAc-18
C6H8NO2
HexNAc-36
C6H10NO3
Hex
Fuc
Hex-18
b4-18
HexNAcHex
y2
b5
y3
HexNAc+NeuAc
y4
b6-18
y5
y6
HexHexNAcFuc
Pep_2+
y7
Pep+HexNAc_2+
Pep+HexNAcFuc_2+
Pep_1+
Pep_1+ -17
Pep+HexNAc_1+
M_4+ - 2NeuGc
Pep+HexNAcFuc_1+
M-36

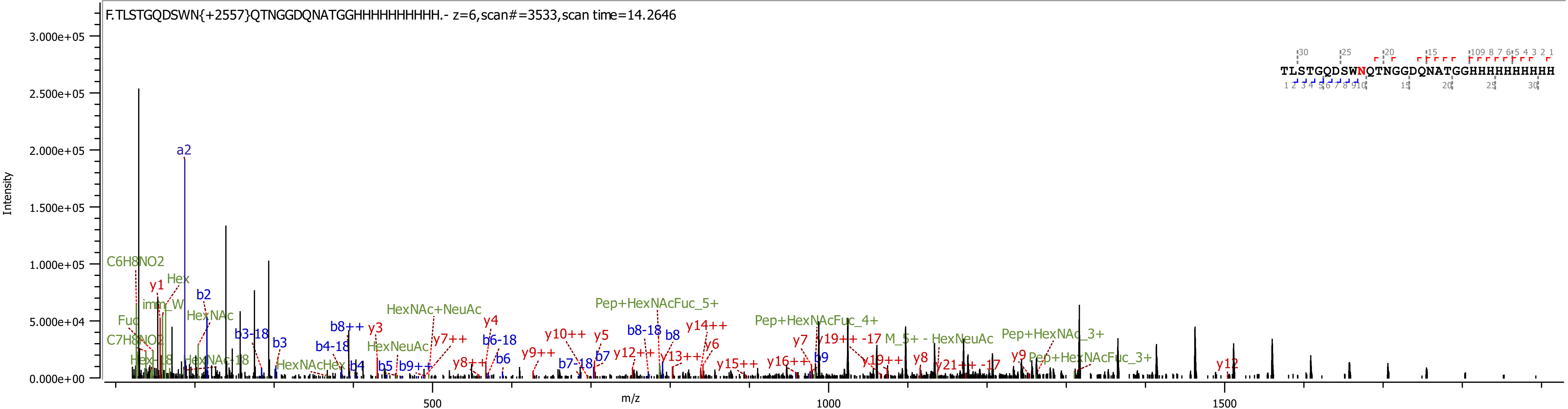

F.TLSTGQDSWN{+2557}QTNGGDQNATGGHHHHHHHHHH.- z=6,scan#=3533,scan time=14.2646
TLSTGQDSWNQTNGGDQNATGGHHHHHHHHHH
Intensity
m/z
3.000e+05
2.500e+05
2.000e+05
1.500e+05
1.000e+05
5.000e+04
0.000e+00
500
1000
1500
a2
C6H8NO2
y1
Hex
imm_W
b2
HexNAc
Fuc
C7H8NO2
Hex-18
HexNAc-18
b3-18
b3
HexNAcHex
b8++
y3
b4-18
b4
HexNAc+NeuAc
HexNeuAc
b5
b9++
y7++
y4
b6-18
b6
y8++
y9++
y10++
y5
b7-18
b7
Pep+HexNAcFuc_5+
b8-18
b8
y12++
y14++
y13++
y6
y15++
Pep+HexNAcFuc_4+
y7
y16++
b9
y19++ -17
M_5+ - HexNeuAc
y19++
y8
y21++ -17
Pep+HexNAc_3+
y9
Pep+HexNAcFuc_3+
y12

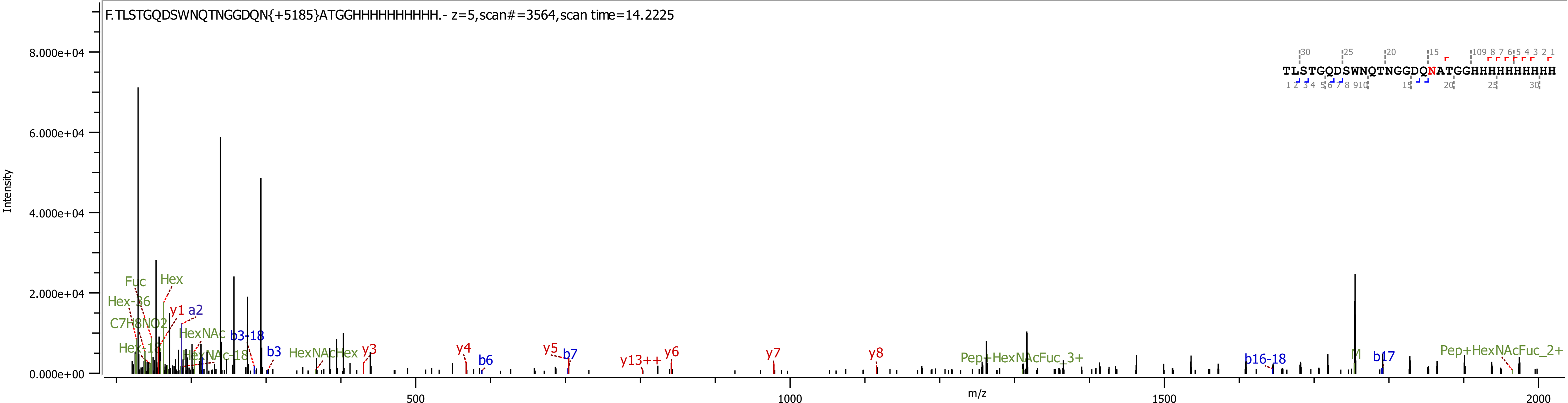
F.TLSTGQDSWNQTNGGDQN{+5185}ATGGHHHHHHHHHH.- z=5,scan#=3564,scan time=14.2225
TLSTGQDSWNQTNGGDQNATGGHHHHHHHHHH
Intensity
m/z
8.000e+04
6.000e+04
4.000e+04
2.000e+04
0.000e+00
500
1000
1500
2000
Fuc
Hex
Hex-36
C7H8NO2
y1
a2
HexNAc
b3-18
b3
HexNAc-18
HexNAc-Hex
y3
y4
b6
y5
b7
y13++
y6
y7
y8
Pep+HexNAcFuc_3+
b16-18
M
b17
Pep+HexNAcFuc_2+

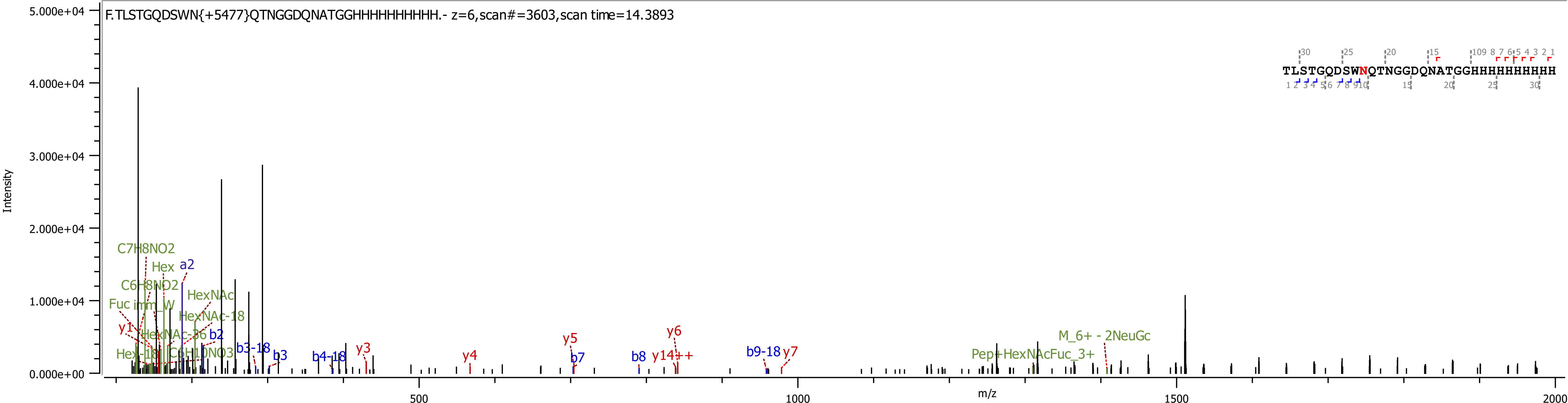

F.TLSTGQDSWN{+5477}QTNGGDQNATGGHHHHHHHHHH.- z=6,scan#=3603,scan time=14.3893
5.000e+04
4.000e+04
3.000e+04
2.000e+04
1.000e+04
0.000e+00
Intensity
m/z
500
1000
1500
2000
TLSTGQDSWNQTNGGDQNATGGHHHHHHHHHH
C7H8NO2
Hex
a2
C6H8NO2
HexNAc
Fuc imm_W
HexNAc-18
y1
HexNAc-36
b2
Hex-18
C6H10NO3
b3-18
b3
b4-18
y3
y4
y5
b7
b8
y6
y14++
b9-18
y7
Pep+HexNAcFuc_3+
M_6+ - 2NeuGc

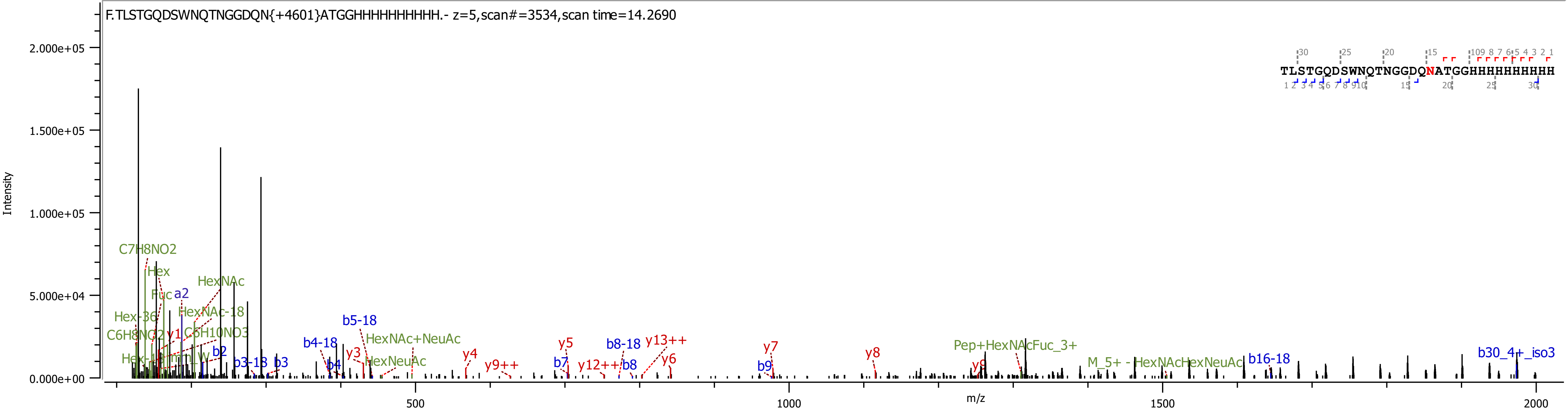
F.TLSTGQDSWNQTNGGDQN{+4601}ATGGHHHHHHHHHHH.- z=5,scan#=3534,scan time=14.2690
Intensity
m/z
TLSTGQDSWNQTNGGDQNATGGHHHHHHHHHHH
C7H8NO2
Hex
Fuc
a2
HexNAc
Hex-36
HexNAc-18
C6H8NO2
y1
C6H10NO3
b2
b3-18
b3
b4-18
b4
b5-18
y3
HexNAc+NeuAc
HexNeuAc
y4
y9++
y5
b7
y12++
b8-18
b8
y13++
y6
y7
b9
y8
Pep+HexNAcFuc_3+
M_5+ - HexNAcHexNeuAc
b16-18
b30_4+_iso3

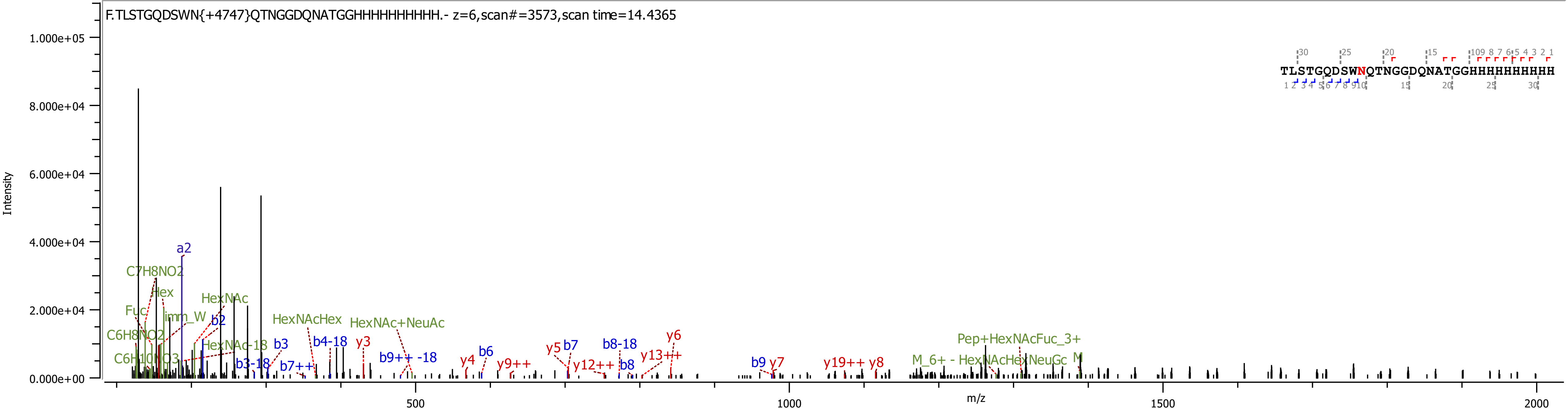

F.TLSTGQDSWN{+4747}QTNGGDQNATGGHHHHHHHHHH.- z=6,scan#=3573,scan time=14.4365
Intensity
m/z
TLSTGQDSWNQTNGGDQNATGGHHHHHHHHHH
a2
C7H8NO2
Hex
HexNAc
Fuc
imm_W
b2
C6H8NO2
HexNAc-18
C6H10NO3
b3
b3-18
b4-18
b7++
HexNAcHex
HexNAc+NeuAc
y3
b9++ -18
y4
b6
y9++
y5
b7
y12++
b8-18
b8
y6
y13++
b9
y7
y19++
y8
Pep+HexNAcFuc_3+
M_6+ - HexNAcHexNeuGc
M

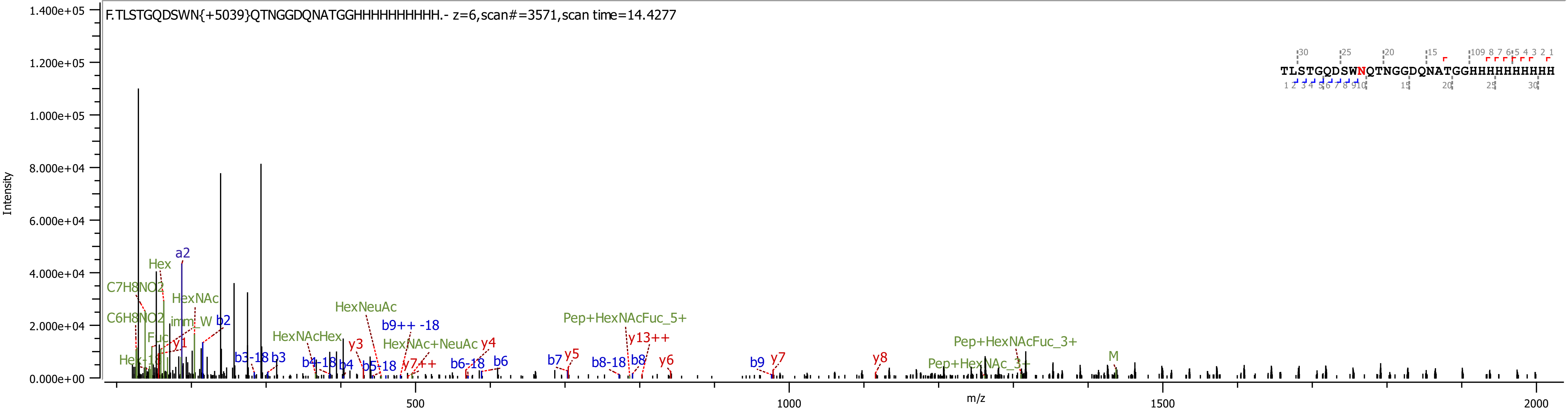
F.TLSTGQDSWN{+5039}QTNGGDQNATGGHHHHHHHHHH.- z=6,scan#=3571,scan time=14.4277
TLSTGQDSWNQTNGGDQNATGGHHHHHHHHHH
Intensity
m/z
1.400e+05
1.200e+05
1.000e+05
8.000e+04
6.000e+04
4.000e+04
2.000e+04
0.000e+00
500
1000
1500
2000
a2
Hex
C7H8NO2
HexNAc
C6H8NO2
imm_W
b2
Fuc
y1
Hex-18
b3-18
b3
HexNAcHex
HexNeuAc
b9++ -18
y3
b4-18
b4
b5-18
HexNAc+NeuAc
y4
y7++
b6-18
b6
b7
y5
Pep+HexNAcFuc_5+
y13++
b8-18
b8
y6
b9
y7
y8
Pep+HexNAcFuc_3+
Pep+HexNAc_3+
M

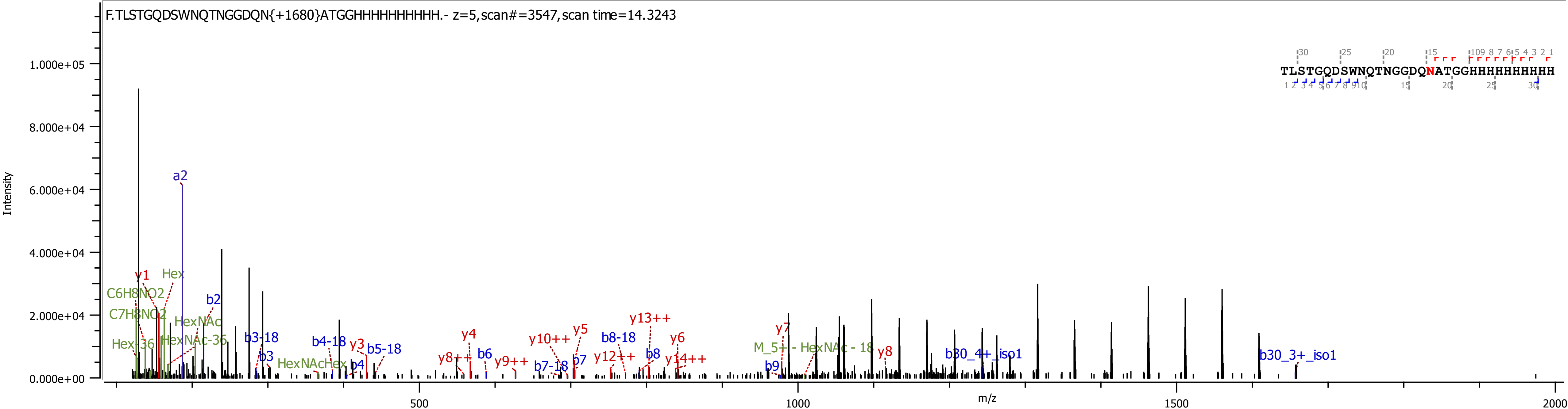
F.TLSTGQDSWNQTNGGDQN{+1680}ATGGHHHHHHHHHH.- z=5,scan#=3547,scan time=14.3243
TLSTGQDSWNQTNGGDQNATGGHHHHHHHHHH
Intensity
m/z
0.000e+00
2.000e+04
4.000e+04
6.000e+04
8.000e+04
1.000e+05
500
1000
1500
2000
a2
y1
Hex
C6H8NO2
C7H8NO2
HexNAc
HexNAc-36
Hex-36
b2
b3-18
b3
HexNAcHex
b4-18
b4
y3
b5-18
y4
y8++
b6
y9++
y10++
y5
b7-18
b7
y12++
b8-18
b8
y13++
y6
y14++
y7
M_5+ - HexNAc - 18
y8
b9
b30_4+_iso1
b30_3+_iso1

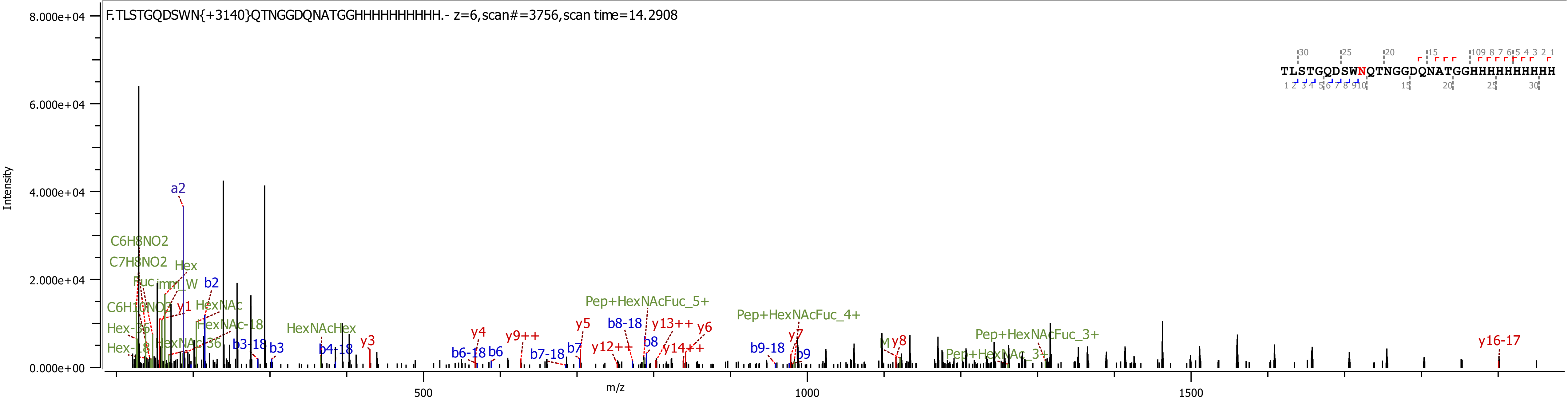

F.TLSTGQDSWN{+3140}QTNGGDQNATGGHHHHHHHHHH.- z=6,scan#=3756,scan time=14.2908
TLSTGQDSWNQTNGGDQNATGGHHHHHHHHHH
Intensity
m/z
8.000e+04
6.000e+04
4.000e+04
2.000e+04
0.000e+00
500
1000
1500
a2
C6H8NO2
C7H8NO2
Hex
Fuc
imm_W
b2
C6H10NO3
y1
HexNAc
Hex-36
HexNAc-18
Hex-18
HexNAc-36
b3-18
b3
HexNAcHex
b4-18
y3
y4
b6-18
b6
y9++
b7-18
b7
y5
y12++
b8-18
b8
y13++
y6
y14++
Pep+HexNAcFuc_5+
Pep+HexNAcFuc_4+
b9-18
y7
b9
M
y8
Pep+HexNAcFuc_3+
Pep+HexNAc_3+
y16-17

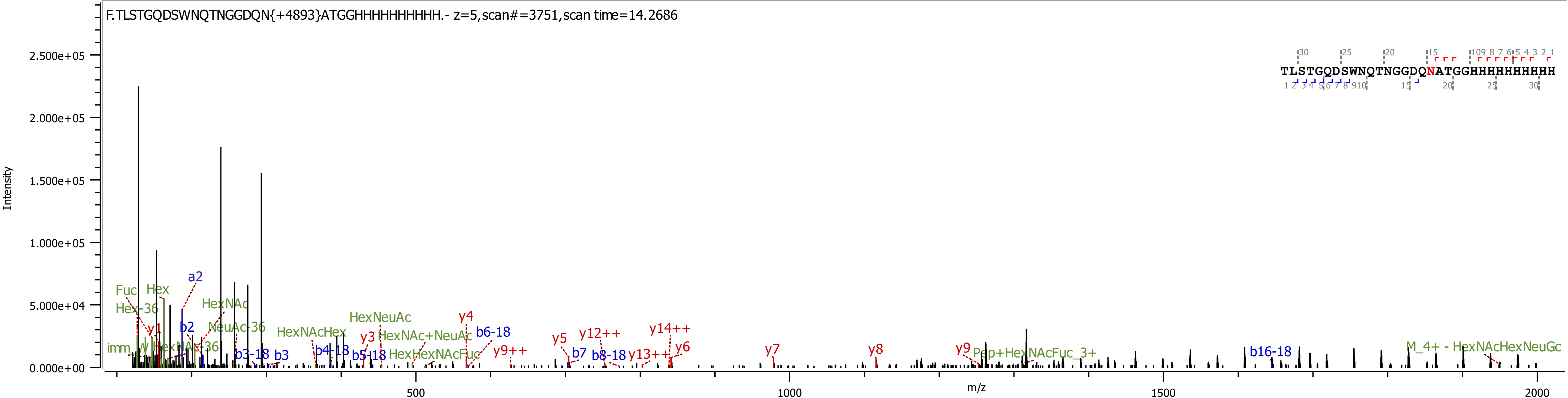

F.TLSTGQDSWNQTNGGDQN{+4893}ATGGHHHHHHHHHH.- z=5,scan#=3751,scan time=14.2686
TLSTGQDSWNQTNGGDQNATGGHHHHHHHHHH
Intensity
m/z
2.500e+05
2.000e+05
1.500e+05
1.000e+05
5.000e+04
0.000e+00
500
1000
1500
2000
Fuc
Hex
Hex-36
a2
HexNAc
b2
y1
NeuAc-36
HexNAcHex
HexNeuAc
y4
y3
HexNAc+NeuAc
b6-18
y5
y12++
y14++
y9++
y6
b7
b8-18
y13++
HexHexNAcFuc
b3-18
b3
b4-18
b5-18
y7
y8
y9
Pep+HexNAcFuc_3+
b16-18
M_4+ - HexNAcHexNeuGc

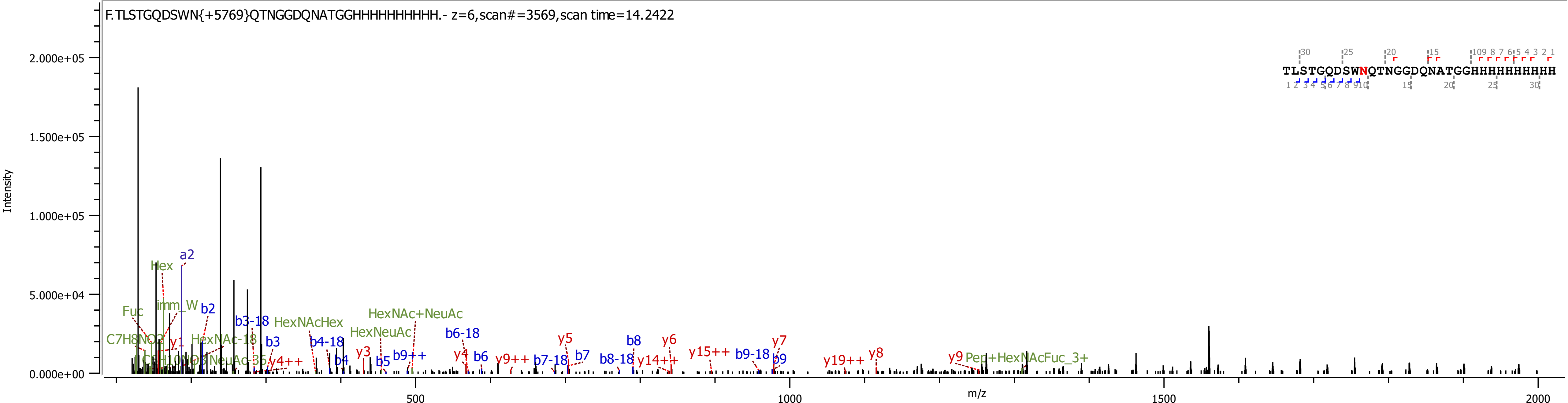
F.TLSTGQDSWN{+5769}QTNGGDQNATGGHHHHHHHHHH.- z=6,scan#=3569,scan time=14.2422
TLSTGQDSWNQTNGGDQNATGGHHHHHHHHHH
Intensity
m/z
Fuc
Hex
a2
imm_W
b2
b3-18
b3
HexNAcHex
HexNAc-18
C7H8NO2
y1
y4++
b4-18
b4
HexNAc+NeuAc
HexNeuAc
b5
b9++
y3
b6-18
y4
b6
y9++
b7-18
b7
y5
b8-18
b8
y6
y14++
y15++
b9-18
b9
y7
y19++
y8
y9
Pep+HexNAcFuc_3+

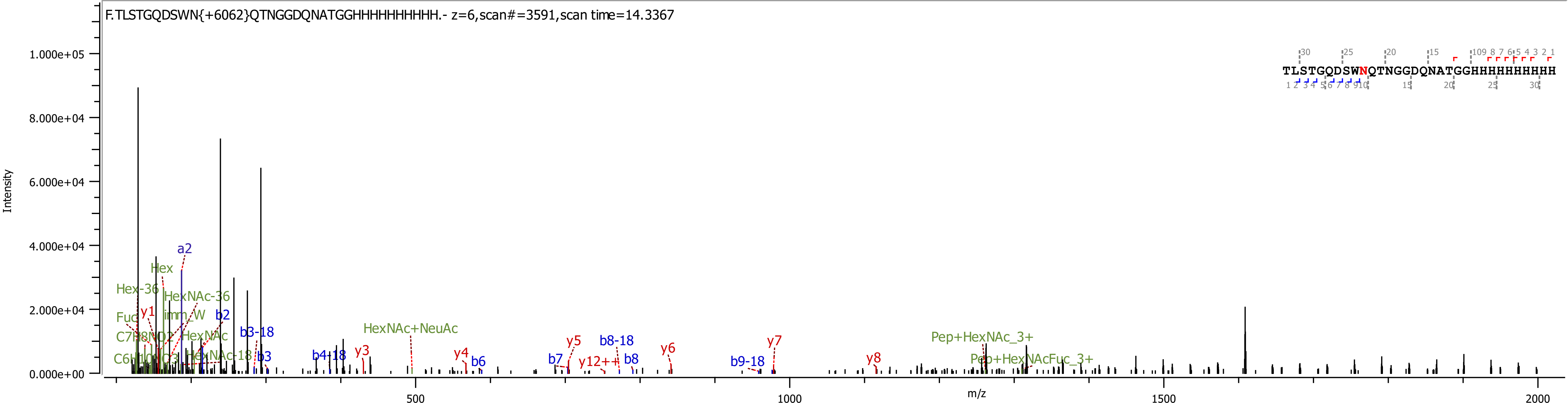
F.TLSTGQDSWN{+6062}QTNGGDQNATGGHHHHHHHHHH.- z=6,scan#=3591,scan time=14.3367
TLSTGQDSWNQTNGGDQNATGGHHHHHHHHHH
Intensity
m/z
1.000e+05
8.000e+04
6.000e+04
4.000e+04
2.000e+04
0.000e+00
500
1000
1500
2000
a2
Hex
Hex-36
HexNAc-36
Fuc
y1
imm_W
b2
C7H8NO
HexNAc
b3-18
HexNAc-18
b3
b4-18
HexNAc+NeuAc
y3
y4
b6
b7
y5
b8-18
y12++
b8
y6
b9-18
y7
y8
Pep+HexNAc_3+
Pep+HexNAcFuc_3+

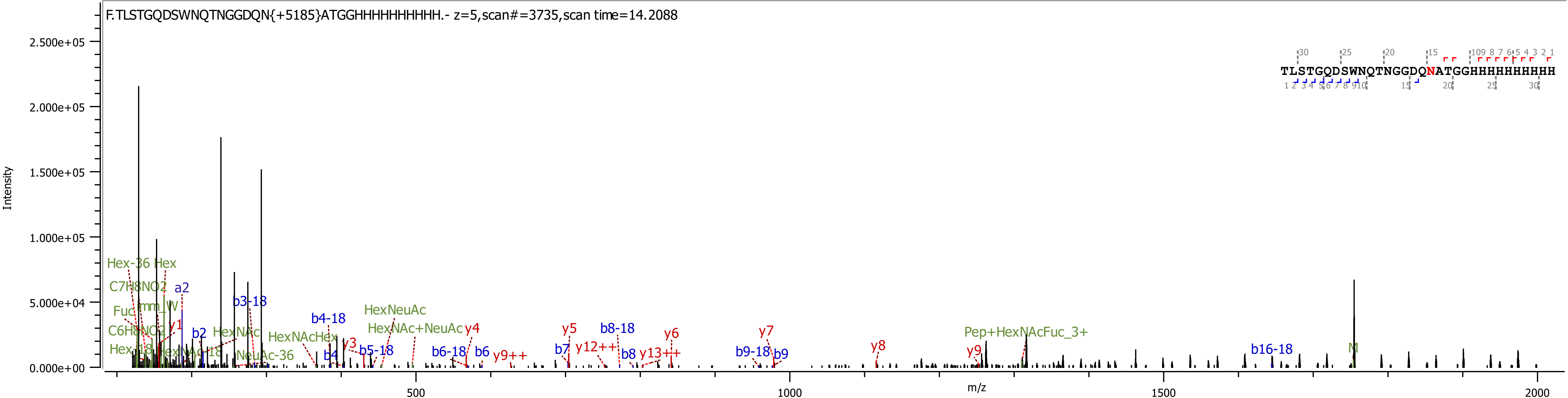

F.TLSTGQDSWNQTNGGDQN{+5185}ATGGHHHHHHHHHH.- z=5,scan#=3735,scan time=14.2088
Intensity
m/z
TLSTGQDSWNQTNGGDQNATGGHHHHHHHHHH
Hex-36 Hex
C7H8NO2
a2
Fuc
Imm_W
y1
C6H8NO2
b2
HexNAc
Hex-18
HexNAc-18
NeuAc-36
b3-18
b4-18
HexNAcHex
b4
y3
b5-18
HexNeuAc
HexNAc+NeuAc
y4
b6-18
b6
y9++
y5
b7
y12++
b8-18
b8
y13++
y6
y7
b9-18
b9
y8
y9
Pep+HexNAcFuc_3+
b16-18
M

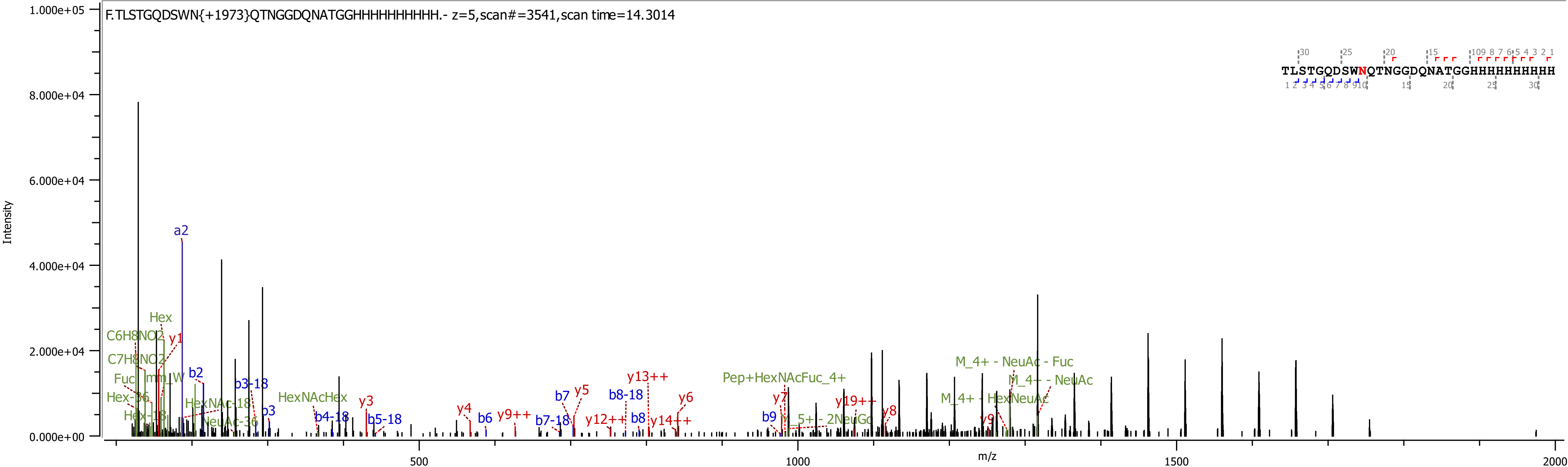
F.TLSTGQDSWN{+1973}QTNGGDQNATGGHHHHHHHHHH.- z=5,scan#=3541,scan time=14.3014
TLSTGQDSWNQTNGGDQNATGGHHHHHHHHHH
Intensity
m/z
1.000e+05
8.000e+04
6.000e+04
4.000e+04
2.000e+04
0.000e+00
500
1000
1500
2000
a2
Hex
C6H8NO2
y1
C7H8NO2
b2
Fuc
Imm_W
Hex-36
Hex-18
HexNAc-18
NeuAc-36
b3-18
b3
HexNAcHex
b4-18
b5-18
y3
y4
b6
y9++
b7
y5
b7-18
b8-18
y12++
b8
y13++
y14++
y6
Pep+HexNAcFuc_4+
y7
b9
Y_5+ - 2NeuGc
y19++
y8
y9
M_4+ - NeuAc - Fuc
M_4+ - NeuAc
M_4+ - HexNeuAc

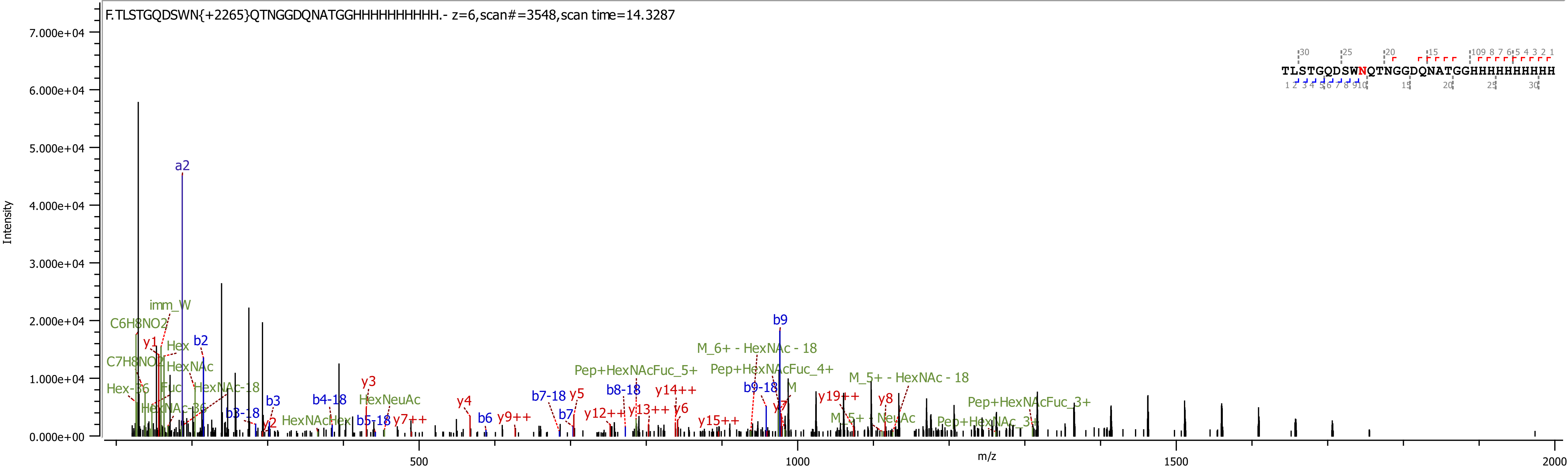
F.TLSTGQDSWN{+2265}QTNGGDQNATGGHHHHHHHHHH.- z=6,scan#=3548,scan time=14.3287
TLSTGQDSWNQTNGGDQNATGGHHHHHHHHHH
Intensity
7.000e+04
6.000e+04
5.000e+04
4.000e+04
3.000e+04
2.000e+04
1.000e+04
0.000e+00
m/z
500
1000
1500
2000
a2
imm_W
C6H8NO2
y1
Hex
b2
C7H8NO2
HexNAc
Hex-36
Fuc
HexNAc-18
HexNAc-36
b3
b3-18
y2
HexNAcHex
b4-18
b5-18
y3
HexNeuAc
y7++
y4
b6
y9++
b7-18
b7
y5
b8-18
y12++
y13++
y14++
y6
y15++
Pep+HexNAcFuc_5+
Pep+HexNAcFuc_4+
M_6+ - HexNAc - 18
b9-18
b9
M
y7
y19++
M_5+ - HexNAc - 18
y8
M_5+ - NeuAc
Pep+HexNAcFuc_3+
Pep+HexNAc_3+

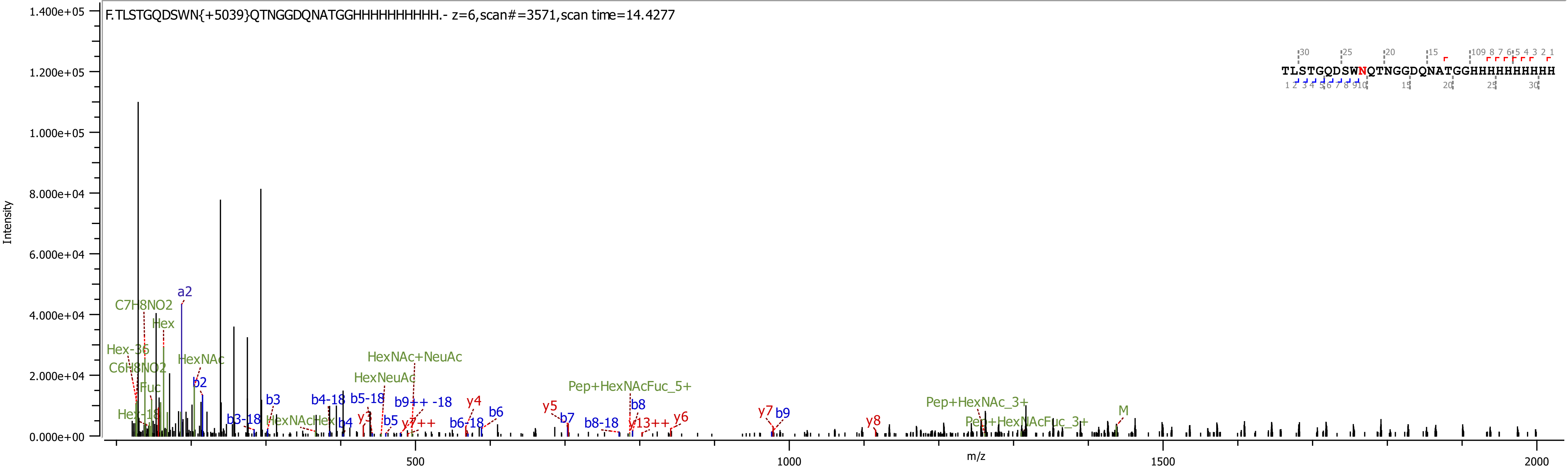

F.TLSTGQDSWN{+5039}QTNGGDQNATGGHHHHHHHHHH.- z=6,scan#=3571,scan time=14.4277
TLSTGQDSWNQTNGGDQNATGGHHHHHHHHHH
Intensity
m/z
C7H8NO2
Hex
Hex-36
C6H8NO2
HexNAc
Fuc
Hex-18
a2
b2
b3
b3-18
HexNAcHex
b4-18
b4
b5-18
b5
HexNAc+NeuAc
HexNeuAc
b9++ -18
y3
y7++
y4
b6-18
b6
y5
b7
Pep+HexNAcFuc_5+
b8
b8-18
y13++
y6
y7
b9
y8
Pep+HexNAc_3+
Pep+HexNAcFuc_3+
M

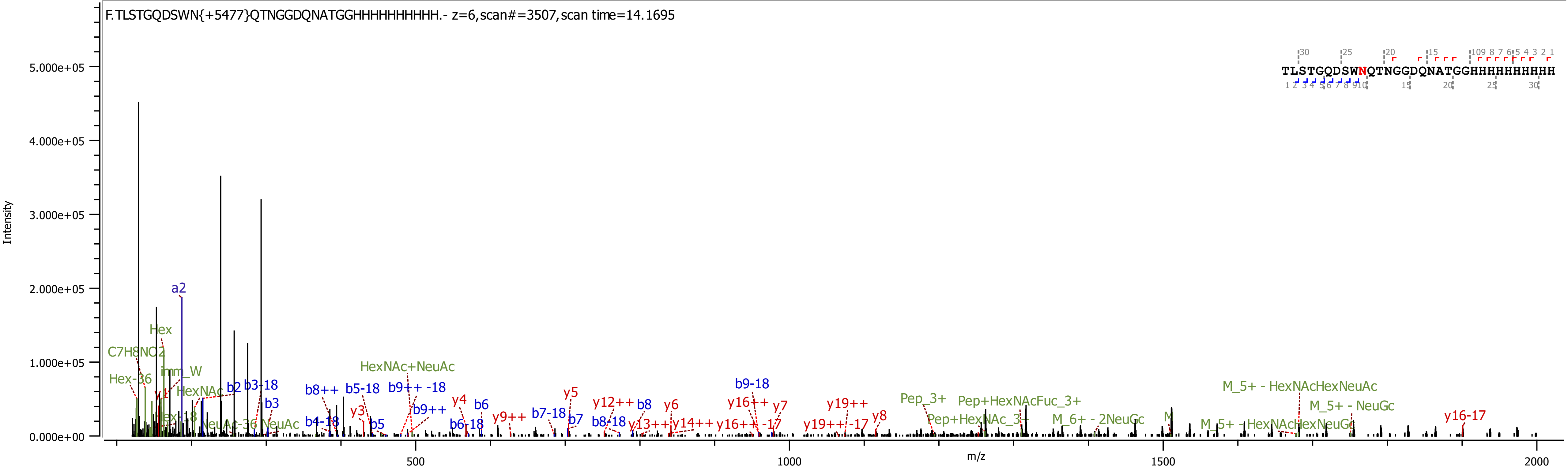
F.TLSTGQDSWN{+5477}QTNGGDQNATGGHHHHHHHHHH.- z=6,scan#=3507,scan time=14.1695
TLSTGQDSWNQTNGGDQNATGGHHHHHHHHHH
Intensity
m/z
0.000e+00
1.000e+05
2.000e+05
3.000e+05
4.000e+05
5.000e+05
500
1000
1500
2000
a2
Hex
C7H8NO2
Hex-36
imm_W
HexNAc
b2
b3-18
b3
NeuAc-36
NeuAc
HexNAc+NeuAc
b8++
b5-18
b9++ -18
b9++
b4-18
y3
b5
y4
b6
b6-18
y9++
b7-18
b7
y5
y12++
b8
b8-18
y13++
y6
y14++
b9-18
y16++
y16++ -17
y7
y19++
y19++ -17
y8
Pep_3+
Pep+HexNAcFuc_3+
Pep+HexNAc_3+
M_6+ - 2NeuGc
M_5+ - HexNAcHexNeuAc
M_5+ - NeuGc
M_5+ - HexNAcHexNeuGc
y16-17
